# Proteomic Analyses Provide Novel Insights into Plant Growth and Ginsenoside Biosynthesis in Forest Cultivated *Panax ginseng* (F. Ginseng)

**DOI:** 10.3389/fpls.2016.00001

**Published:** 2016-01-26

**Authors:** Rui Ma, Liwei Sun, Xuenan Chen, Bing Mei, Guijuan Chang, Manying Wang, Daqing Zhao

**Affiliations:** ^1^Jilin Technology Innovation Center for Chinese Medicine Biotechnology, College of Chemistry and Biology, Beihua UniversityJilin, China; ^2^Ginseng Research Center, Changchun University of Chinese MedicineChangchun, China; ^3^The first affiliated hospital to Changchun University of Chinese MedicineChangchun, China

**Keywords:** *Panax ginseng*, energy metabolism, ginsenosides biosynthesis, growth, proteomic analysis

## Abstract

F. Ginseng (*Panax ginseng*) is planted in the forest to enhance the natural ginseng resources, which have an immense medicinal and economic value. The morphology of the cultivated plants becomes similar to that of wild growing ginseng (W. Ginseng) over the years. So far, there have been no studies highlighting the physiological or functional changes in F. Ginseng and its wild counterparts. In the present study, we used proteomic technologies (2DE and iTRAQ) coupled to mass spectrometry to compare W. Ginseng and F. Ginseng at various growth stages. Hierarchical cluster analysis based on protein abundance revealed that the protein expression profile of 25-year-old F. Ginseng was more like W. Ginseng than less 20-year-old F. Ginseng. We identified 192 differentially expressed protein spots in F. Ginseng. These protein spots increased with increase in growth years of F. Ginseng and were associated with proteins involved in energy metabolism, ginsenosides biosynthesis, and stress response. The mRNA, physiological, and metabolic analysis showed that the external morphology, protein expression profile, and ginsenoside synthesis ability of the F. Ginseng increased just like that of W. Ginseng with the increase in age. Our study represents the first characterization of the proteome of F. Ginseng during development and provides new insights into the metabolism and accumulation of ginsenosides.

## Introduction

Ginseng (*Panax ginseng* C. A. Meyer) is a perennial plant that has long been used in Chinese herbal medicine. The main part of ginseng that has therapeutic effects is the root. Ginseng root has clinical and pharmacological effects such as anti-aging activity, anticancer activity, protection against circulatory shock effects; it also promotes immune function in human beings (Wang et al., 2005; Li et al., 2012; Kim and Cho, 2013; Bae et al., 2014). However, wild-type ginseng (W. Ginseng) is very scarce and unsustainable. Ginseng growing in forests (F. Ginseng) is a type of ginseng cultivar, which is planted to alleviate the resource scarcity and ensure the external morphology.

F. Ginseng requires 15–20 years or even longer to attain the medicinal properties with efficacy similar to that of W. Ginseng (Li, 2008). F. Ginseng undergoes dramatic changes in size, weight, composition, and accumulating ginsenosides in the root over time (Deng et al., 2013). Folklore suggests that a change in the morphology of F. Ginseng is a good indicator of its growth period, and helps to assess its effectiveness and differences as compared with W. Ginseng. Moreover, recent studies the content of ginsenosides-the main active substances-increases every improve its medicinal value (Soldati and Tanaka, 1984; Chan et al., 2000; Lee et al., 2001; Lin et al., 2010). Thus, the medicinal value of F. Ginseng is positively correlated to its age. The longer the growth time, the more similar F. Ginseng is to W. Ginseng. Therefore, comparative studies on the growth and development of F. Ginseng with W. Ginseng will provide deeper insights into the molecular mechanism of ginsenoside formation.

In fact, the plant growth is the summation of biochemical and physiological changes. These changes include synthesis of sugars, alterations in secondary metabolite biosynthesis, response to stress, and accumulation of antioxidant compounds (Giovannoni, 2004). Differential proteomic approaches enable the identification of protein species with changes in abundance levels during the process of growth. This allows the identification of proteins that are specifically relevant to the control of the metabolic pathways responsible for plant growth and development (Andrade et al., 2012). Major protein variations that occur during plant development, ripening and response to stress are well-studied in many commercially important plants including the model plant *Arabidopsis thaliana* (Lee et al., 2012), rice (Nozu et al., 2006), tomato (Rocco et al., 2006; Faurobert et al., 2007), and orange (Bianco et al., 2009). Previously, studies on ginseng focused on differences between cultivars (Lum et al., 2002; Nagappan et al., 2012). The protein changes that occur in ginsengs during growth and ginsenosides biosynthesis are not well-studied.

Traditionally, two-dimensional polyacrylamide gel electrophoresis (2DE) has been the gold standard for proteomic analysis. However, this platform is limited by protein identification and quantification capabilities. For instance, the low-abundance proteins, such as membrane proteins and hydrophobic proteins are difficult to detect on 2D gel-electrophoresis (Zieske, 2006). To overcome the disadvantage of this technique, non-gel-based quantitative proteomic methods have been developed in recent years. Isobaric mass tagging (e.g., iTRAQ) is a precise and sensitive multiplexed peptide/protein quantification technique in mass spectrometry (Ghosh et al., 2013), which has been extensively used for revealing the differentially expressed proteins under any given conditions including plant growth and development (Fukao et al., 2011), and biotic and abiotic stresses (Wang et al., 2014; Li et al., 2015).

In the present study, we used a proteomic approach involving 2DE and iTRAQ to investigate the differentially expressed proteins during F. Ginseng root growth, analyze the changes on metabolic process related to growth, and investigate the relationship between F. Ginseng and W. Ginseng. Our findings will help in understanding the molecular mechanism of ginsenoside biosynthesis. Moreover, our results can also be extrapolated to studying the medicinal use of F. Ginseng.

## Materials and methods

### Plant materials

Up to 80% of the world's total supply of F. Ginseng roots comes from the major ginseng farming region in the Jilin Province, China. Therefore, F. Ginseng roots were collected from August in 2014 from the FuSong County in Jilin Province. This area is located at 127 degrees, 46 min east longitude and 42°, 48 min north latitude and is situated in a humid mountainous climate at an altitude of 520 m. F. Ginseng samples were collected during four kinds of growth years: 10, 15, 20, and 25 years. Fifteen samples/years were taken and frozen in liquid nitrogen. W. Ginseng roots were provided by Wujie wild ginseng planting base located in the FuSong county. Ten W. Ginseng roots samples were 40 years old, collected at 127° 30–50 min east longitude and 35–58 min north latitude. The dissected samples were immediately frozen in liquid nitrogen.

### Estimation of growth parameters and phenotypic plasticity indexes

**Figure 1:**
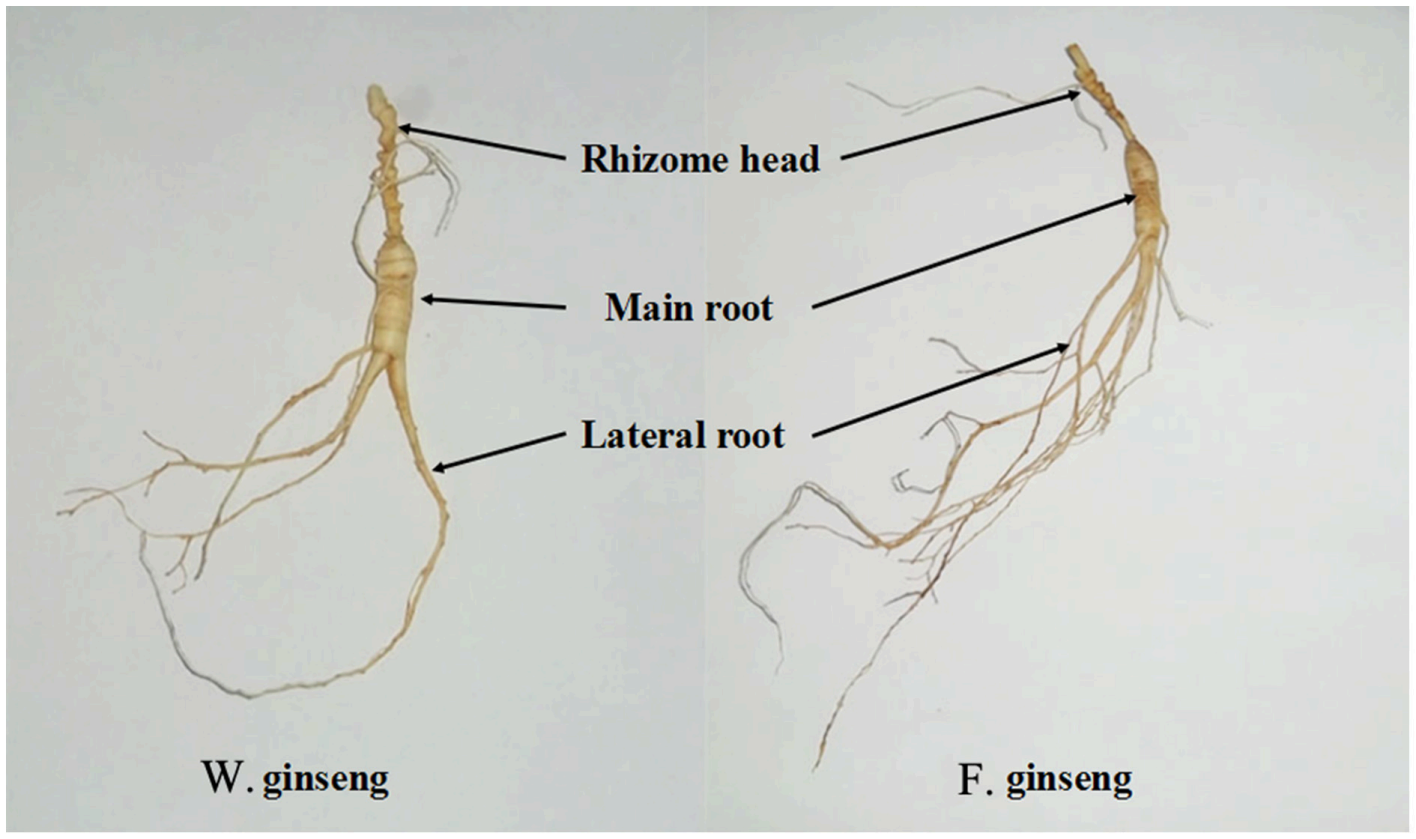
**Different parts of W. Ginseng and F. Ginseng**.

As described previously (Lum et al., 2002), we divided ginseng into its main root (MR), lateral root (LR) and rhizome head (RH) (Figure 1). Different parts were weighed on an electronic scale (0.01 g), and the root mass ratio (RMR) was calculated (g·g^−1^) (Gregory et al., 1995). We placed the plant parts on a glass board covered with graph paper to measure their length (0.1 mm) and calculated the specific root length (SRL) (cm·g^−1^) (Ostonen et al., 2007). The total length and biomass were determined as the sum of every part. Average values were calculated from 25 samples per developmental stage of F. Ginseng. Relative growth rate (RGR) was measured as the increase in mass per biomass per year and was calculated using the following equation: RGR = (ln W2 − ln W1) / (t2 − t1); where ln = natural logarithm, t1 = time one (in years), t2 = time two (in years), W1 = weight of plant at time one (in grams), W2 = weight of plant at time two (in grams). The phenotypic plasticity index [PPI, (F. Ginseng mean − W. Ginseng mean)/W. Ginseng mean] was calculated for each trait (Caplan and Yeakley, 2013), which was used to evaluate the morphological difference between F. Ginsengs (in different growth time) and W. Ginseng.

### Protein extraction

The ginseng root proteins were extracted using a phenol procedure (Wang et al., 2009). Ground tissue was precipitated with cold acetone with 0.07% b-mercaptoethanol (at least three times) and resuspended in lysis buffer [7 M urea, 2 M thiourea, 2% (w/v) CHPAS, 1% (w/v) plant protease inhibitor]. Then, an equal volume of tris-saturated phenol was added and centrifuged at 10600 g/min at 4°C for 15 min, and the water phase was discarded. The phenol phase was washed with methanol containing 0.1 M ammonium acetate and acetone two and three times, respectively. After the complete evaporation of acetate, the proteins were dissolved in the appropriate volume of rehydration solution [5 M urea, 2 M thiourea, 2% (w/v) CHAPS, 2% (w/v) N-decyl-N,Ndimethyl-3-ammonio-1-propane-sulfonate (SB3-10)] (Chinnasamy and Rampitsch, 2006). The protein concentrations were measured using Bradford's method.

### 2DE and image analysis

The protein samples were first separated by isoelectric focusing using linear precast IPG strips (24 cm, 3–10 linear pH gradients, GE Healthcare, UK). IPG strips with 1.2 mg of protein were rehydrated for 12 h. and focused at 72,000 Vhs, as described previously (Sun et al., 2011). Focusing was performed under the following conditions: a rapid gradient increase from 30 to 200 V for 1 h, 200 to 500 V for 1 h and then a linear increase from 500 to 1000 V for 2 h, 8000 V for 4 h, and, at last, a rapid gradient of 8000 V until 60,000 V.h. After IEF, first-dimension strips were equilibrated immediately. Second-dimension SDS–PAGE was performed using 12.5% polyacrylamide gels at 2 W per gel for 30 min and 15 W per gel for 5–6 h in six EttanDalt systems (GE Healthcare, UK). Finally, the gels were stained in the staining solution (0.05% CBBR in 25% isopropanol and 10% acetic acid) for 6 h or overnight and destained by 10% (v/v) ethanol and 10% (v/v) acetic acid until the background was clear (Borejdo and Flynn, 1984; Westermeier, 2006; Yang et al., 2016). The stained gels were scanned by a 600 dpi Image Scanner (GE Healthcare, UK). All spots were matched by gel-to-gel comparison using Image Master 2D Platinum Software Version 6.0 (GE Healthcare, UK). The spots with statistically significant (Student's *t*-test with a *P* < 0.05) and reproducible changes (quantitative changes > 1.5-fold in abundance) in abundance were considered to be differentially expressed protein spots.

Significant differences were analyzed using a two-way hierarchical clustering methodology using the PermutMatrix software (Meunier et al., 2007). For this purpose, the data produced by the analysis of 2DE gels were converted into a binary matrix, and the missing values were replaced with zeros (Negri et al., 2008). The row-by-row normalization of data was performed using the classical zero-mean and unit-standard deviation technique. Pearson's distance and Ward's algorithm were used for the analysis.

### Two-dimensional gel excision, tryptic digestion, and desalting

Protein extracts were separated on preparative gels and proteins of interest were recovered from the gels for identification. Proteins from the different years of F. Ginseng samples were resolved on separate preparative polyacrylamide gels and were visualized by staining with a Coomassie blue staining method compatible with subsequent mass-spectrometric analysis. All of the differentially expressed spots were selected and excised manually from the four preparative gels. Protein spots of interest were cut from the preparative gels de-stained for 20 min in 25 mM NH_4_HCO_3_/50% acetonitrile and washed with Milli-Q water until the gels were de-stained. The spots were incubated in 0.2 M NH_4_HCO_3_ for 20 min and then lyophilized. Each spot was digested overnight in 12.5 ng/μl trypsin in 25 mM NH_4_HCO_3_. The peptides were extracted three times with 60% acetonitrile (ACN)/0.1% trifluoroacetic acid (TFA). The extracts were pooled and dried completely using a vacuum centrifuge.

### Protein identification by MALDI-TOF/TOF MS

MS and MS/MS data for protein identification were obtained by using a MALDI-TOF-TOF instrument (4800 proteomics analyzer; Applied Biosystems). Instrument parameters were set using the 4000 Series Explorer software (Applied Biosystems). The MS spectra were recorded in reflector mode in a mass range from 800 to 4000 with a focus mass of 2000. MS was used a CalMix5 standard to calibrate the instrument (ABI 4700 Calibration Mixture). For one main MS spectrum, 25 sub-spectra with 125 shots per sub-spectrum were accumulated using a random search pattern. For MS calibration, autolysis peaks of trypsin ([M+H]+842.5100 and 2211.1046) were used as internal calibrates, and up to 10 of the most intense ion signals were selected as precursors for MS/MS acquisition, excluding the trypsin autolysis peaks and the matrix ion signals. In MS/MS positive ion mode, for one main MS spectrum, 50 sub-spectra with 50 shots per sub-spectrum were accumulated using a random search pattern. The collision energy was 2 kV, collision gas was air, and default calibration was set by using the Glu1-Fibrino-peptide B ([M+H]^+^ 1570.6696) spotted onto Cal 7 positions of the MALDI target. Combined peptide mass fingerprinting PMF and MS/MS queries were performed by using the MASCOT search engine 2.2 (Matrix Science, Ltd.), embedded into GPS-Explorer Software 3.6 (Applied Biosystems) on the NCBI viridiplantae database with the following parameter settings: 100 ppm mass accuracy, trypsin cleavage one missed cleavage allowed, carbamidomethylation set as fixed modification, oxidation of methionine was allowed as variable modification, MS/MS fragment tolerance was set to 0.4 Da. A GPS Explorer protein confidence index ≥95% were used for further manual validation.

### iTRAQ analysis

Protein (100 mg) was reduced by adding dithiothreitol to a final concentration of 10 mM and incubated for 1 h at room temperature. Subsequently, iodoacetamide was added to a final concentration of 40 mM, and the mixture was incubated for 1 h at room temperature in the dark. Then, dithiothreitol (10 mM) was added to the mixture to consume any free iodoacetamide by incubating the mixture for 1 h at room temperature in the dark. Proteins were then diluted in 50 mM triethylammonium bicarbonate and 1 mM CaCl_2_ to reduce the urea concentration to less than 0.6 M and digested with 40 mg of modified trypsin at 37°C overnight. The resulting peptide solution was acidified with 10% trifluoroacetic acid and desalted on a C18 solid-phase extraction cartridge.

Desalted peptides were then labeled with iTRAQ reagents (Applied Biosystems) according to the manufacturer's instructions. Ten-year-old ginsengs were labeled with reagent 114; 15-year-old ginsengs were labeled with reagent 115; 20-year-old ginsengs were labeled with reagent 116, and 25-year-old ginsengs were labeled with reagent 117. The reaction was allowed to proceed for 1 h at room temperature.

Subsequently, Nano-HPLC-MS/MS analysis was performed on a nanoAcquity system (Waters) connected to an LTQ-Orbitrap XL hybrid mass spectrometer (Thermo Electron) equipped with a PicoView nanospray interface (New Objective). Peptide mixtures were loaded onto a 75-mm i.d., 25-cm length C18 BEH column (Waters) packed with 1.7-mm particles with a pore size of 130 A°. They were separated using a segmented gradient in 90 min from 5 to 40% solvent B (acetonitrile with 0.1% formic acid) at a flow rate of 300 nL /min and a column temperature of 35°C. Solvent A was 0.1% formic acid in water. The LTQ-Orbitrap XL hybrid mass spectrometer was operated in positive ionization mode. The MS survey scan for all experiments was performed in the Fourier transform cell recording a window between 350 and 1600 mass to charge ratio (m/z). The resolution was set to 60,000 at m/z 400, and the automatic gain control was set to 500,000 ions. The m/z values triggering MS/MS were put on an exclusion list for 90 s. The minimum MS signal for triggering MS/MS was set to 5000. In all cases, one microscan was recorded. For high-energy collision dissociation, the applied acquisition method consisted of a survey scan to detect the peptide ions followed by a maximum of three MS/MS experiments of the three most intense signals exceeding a minimum signal of 5000 in survey scans. For MS/MS, we used a resolution of 7500, an isolation window of 2 m/z, and a target value of 100,000 ions, with maximum accumulation times of 400 ms. Fragmentation was performed with a normalized collision energy of 50% and an activation time of 30 ms. We performed three technical replications for each experiment.

### Database search and quantification

The 2.3.02 version of the Mascot software (Matrix Science) was used to identify and quantify proteins simultaneously. In this version, unique peptides used for protein quantification can be chosen, which is more precise to quantify proteins. Searches were made against the green plants protein database (TAIR9_pep_20090619, 33,410 sequences; ftp://ftp.arabidopsis.org/home/tair/Sequences/blast_datasets/TAIR9_blastsets/) concatenated with a decoy database containing the randomized sequences of the original database. For each technical repeat, spectra from the 20 fractions were combined into one MGF (Mascot generic format) file after loading the raw data, and the MGF file were searched. For biological repeats, spectra from the three technical repeats were combined into one file and searched. The search parameters were as follows: trypsin/P was chosen as the enzyme with two missed cleavages allowed; fixed modifications of carbamidomethylation at Cys, variable modifications of oxidation at Met and iTRAQ 4plex at Tyr; peptide tolerance was set at 10 ppm, and MS/MS tolerance was set at 0.6 D. Peptide charge was set Mr, and monoisotopic mass was chosen. iTRAQ 4plex was chosen for quantification during the search simultaneously. The search results were passed through additional filters before exporting the data. For protein identification, the filters were set as follows: Significance threshold P, 0.05 (with 95% confidence) and ion score or expected cutoff less than 0.05 (with 95% confidence). For protein quantitation, the filters were set as follows: “Weighted” was chosen for protein ratio type (http://mascot-pc/mascot/help/quant_config_help.html); minimum precursor charge was set to 1, and minimum peptides were set to 2; unique peptides were used to quantify proteins. Summed intensities were set as normalization, and outliers were removed automatically. The peptide threshold was set as above for homology.

### Enzymatic activity analysis

Amylase (AMY, EC 3.1.1.2) activity was detected by the 3,5-dinitrosalicylic acid colorimetric method (Hao et al., 2007). Malate dehydrogenase (MDH, EC 1.1.1.37) activity was examined as described by Husted and Schjoerring (1995), with some modifications. Ten microliter samples were added to a 3 ml reaction mixture containing 0.17 mM oxalacetic acid and 0.094 mM β-NADH disodium salt in 0.1 M Tris buffer, pH 7.5. The reaction was measured by the decrease in absorbance at 340 nm for 180s in a spectrophotometer (Hitachi U-2001 Japan), the same reaction system only with sample buffer added in was used as a blank. Superoxide dismutase (SOD, EC 1.14.1.1) activity was measured according to the method of Zhang and Kirkham (1996), and Xu and Huang (2004). One unit of SOD activity is defined as the amount of SOD required to cause 50% inhibition of nitroblue tetrazolium (NBT) reduction at 560 nm min-1. Catalase (CAT, EC 1.11.1.6) and peroxidase (POD, EC.1.11.1.7) activity were determined based on the method of Chance and Maehly (1955) as described in detail for creeping bentgrass in Xu and Huang (2004). Enzyme activities were based on the absorbance change of the reaction solution per minute at a given wavelength for each enzyme: CAT at 240 nm and POD at 470 nm.

The activities of farnesyl diphosphate synthase (FDPS, EC. 2.5.1.10), cycloartenol synthase (CAS, EC. 5.4.99.8), squalene epoxidases (SE, EC:1.14.13.132), and squalene synthase (SS, EC. 2.5.1.21) involved in ginsenosides biosynthesis, were quantified by an indirect competitive enzyme-linked immunosorbent assay (ELISA). The optical density (OD) values of each sample were read by a BioTek ELx800 microplate reader at 450 nm. The primary concentration of each test sample was calculated from the linear regression equation based on the OD values of the standards.

### Metabolite content analyses

Starch was measured via an enzyme hydrolysis method. Starch was hydrolyzed into dual sugars by amylase, hydrolyzed into monosaccharides by hydrochloric acid, and finally determined by reducing sugar, which is converted to starch (Rose et al., 1991).

The contents of pyruvate in the sample were determined according to the methods of Lin et al. (1995). Protein was removed from the samples by TCA precipitation, and in the resulting sample, pyruvate reacted with 2,4-nitrophenylhydrazine. The product turned red in the presence of an alkali solution, and the intensity of the color change was measured by a spectrophotometer. A standard curve for calibration was obtained using sodium pyruvate as a reagent with a gradient of concentrations of pyruvic acid. Absorbance values were obtained to generate a standard curve to calculate the pyruvate concentration.

For glutathione (GSH), roots were ground in liquid nitrogen and homogenized in 1 mL 5% (w/v) m-phosphoric acid containing 1 mM diethylene triamine pentaacetic acid (DTPA) and 6.7% (w/v) sulfosalicylic acid. Root extracts were centrifuged at 12,000 × g for 15 min at 4°C. GSH contents were determined according to the methods of Kortt and Liu (1973) and Ellman (1959) with some modifications.

The ascorbic acid (AsA) content was determined according to Egea et al. (2007) with slight modifications. Ginseng roots were ground in an ice bath with 10 mL 5% metaphosphoric acid stored at 4°C, and then the final mix was homogenized by vortex. The final solution was maintained on the ice bath, in darkness, for 30 min and then centrifuged at 20,000 × g for 25 min at 4°C. Ascorbate was spectrophotometrically detected by measuring absorbance at 254 nm with a UV detector. For quantification of the compound, a calibration curve in the range of 10–100 mg kg^−1^ was prepared from standard ascorbic acid. Results were expressed as mg 100 g^−1^ FW.

Root extracts were centrifuged at 12,000 × g for 15 min at 4°C. The extraction and determination of ginsenosides was performed following the method of Yu et al. (2002).

### Semi-quantitative RT-PCR analysis

Total RNA was extracted from various P. ginseng samples—collected over various growth years using RNeasy mini kit (Takara Bio, China). RT-PCR was conducted using 200 ng of total RNA as a template for reverse transcription using oligo(dT)15 primer (0.2 mM) and AMV Reverse Transcriptase (10 U/μl) (Takara Bio, China) according to the manufacturer's instructions. RT-PCR was performed using a 1-μl aliquot of the first strand cDNA in a final volume of 20 μl reaction volume. Five pmol of specific primers for pathogenesis-related protein gene (PR5) and glutaredoxin gene (Grx) were used for performing PCR. The actin gene (ACT) primers were used as internal control (Table S1). The thermal cycler conditions recommended by the manufacturer were used as follow: an initial denaturation for 10 min at 94°C, 35 amplification cycles (30 s at 94°C, 30 s at 58°C, and 30 s at 72°C), followed by a final elongation for 10 min at 72°C. Ten microliters of the reaction mixture were analyzed on a 1% (W/V) agarose gel in 1 × TAE buffer and then photographed for expression analysis.

Images of the RT-PCR ethidium bromide-stained agarose gels were acquired with a Cohu High Performance CCD camera (Cohu Inc. San Diego, CA). The quantification of bands was performed by Phoretix 1 D (Phoretix International Ltd., Newcastle upon Tyne, UK). Band intensity was expressed as relative absorbance units. The ratio between the sample RNA to be determined and actin was calculated to normalize for initial variations in sample concentration and as a control for reaction efficiency. Means and standard deviations of all experiments performed were calculated after normalization to actin.

### Statistical analysis

Values in figures and tables are expressed as the mean ± SD. Statistical analysis was carried out with three biological replicates for proteomic and biochemical analyses. The results of the spot intensities and physiological data were statistically analyzed by a One-way ANOVA and the Duncan's new multiple range test (DMRT) to determine the significance of differences between group means. *P* ≤ 0.05 was considered statistically significant (SPSS for Windows, version 12.0).

## Results

### Morphological changes in F. Ginseng growth

**Figure 2:**
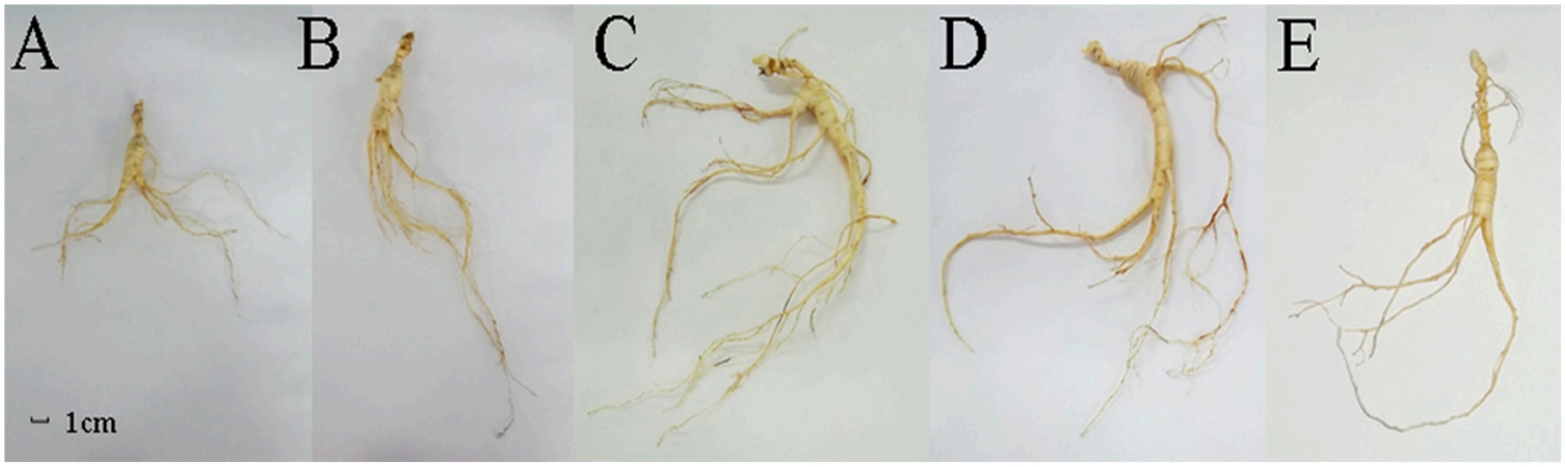
**Morphological features of F. Ginseng in different growth years: 10 years old (A), 15 years old (B), 20 years old (C) and 25 years old (D), and W. Ginseng (E)**.

In order to analyze the changes in physiological shape along with the growth years, we tested the growth parameters of F. Ginseng root (Table 1). The morphological characteristics of F. Ginseng showed that the total length (TL) increased with years of growth while specific root length (SRL), specific main root length (SMRL), specific lateral root length (SLRL) and specific rhizome head length (SHRL) demonstrated an opposite trend. The SRL of young F. Ginseng (Figures 2A,B) was greater than that of older F. Ginseng (Figures 2C,D), which decreased significantly due to yearly increases in weight and diameter to guarantee root absorption during the period of vigorous growth (Maurice et al., 2010), similar to W. Ginseng (Figure 2E).

**Table 1:** **Growth parameters and phenotypic plasticity indexes of F. Ginsengs during different growth years and W. Ginseng**.

	**F. Ginseng**				**W. Ginseng**	**Phenotypic plasticity index, PPI**			
	**Year 10**	**Year 15**	**Year 20**	**Year 25**		**10 years F. Ginseng/W. Ginseng**	**15 years F. Ginseng/W. Ginseng**	**20 years F. Ginseng/W. Ginseng**	**25 years F. Ginseng/W. Ginseng**
**MORPHOLOGICAL TRAITS**									
Total length, TL, cm	22.63	24.92	26.53	30.04	29.64	0.24	0.16	0.10	0.01
Specific root length, SRL, cm/g	5.74	2.81	2.06	1.80	2.00	1.87	0.41	0.03	0.10
Specific main root length, SMRL, cm/g	1.46	0.85	0.69	0.65	0.68	1.15	0.24	0.02	0.04
Specific lateral root length, SLRL, cm/g	10.30	9.53	6.95	5.83	5.25	0.96	0.82	0.32	0.11
Specific rhizome head length, SRHL, cm/g	9.17	3.21	1.98	2.10	1.69	4.42	0.90	0.17	0.24
**ALLOCATION TRAITS**									
Total biomass, TB, g	3.94	8.86	12.87	16.66	14.81	0.73	0.40	0.13	0.12
Main root mass ratio, MRMR, g/g	0.51	0.70	0.69	0.69	0.57	0.11	0.22	0.20	0.21
Lateral root mass ratio, LRMR, g/g	0.43	0.20	0.19	0.19	0.25	0.73	0.21	0.22	0.25
Rhizome head mass ratio, RRMR, g/g	0.06	0.11	0.12	0.12	0.18	0.66	0.41	0.33	0.34
**GROWTH TRAITS**									
Relative growth rate, RGR	—	0.16	0.07	0.05	—	—	—	—	—

Biomass is based on energy and the accumulation of nutrients (Guo et al., 2002). The increase in total biomass (TB) of F. Ginseng was mainly allocated to the main root and rhizome head, and the lateral root mass ratio (LRMR) decreased year by year (Table 1). This was mainly due to the degeneration of most of the tiny lateral roots and the survival of several strong roots (Figure 2), which enabled the plant to adapt to the environment by reducing the risk of root damage.

The RGR can capture dynamic changes in the physiology and morphology of F. Ginseng and is used to quantify the speed of plant growth (Useche and Shipley, 2010). The results showed that the RGR decreases over time as the biomass of F. Ginseng increases and then becomes stable in mature ginseng, consistent with the other growth parameters (Table 1).

The year-to-year variation of SLRL and SHRL in F. Ginseng growing is closer to W. Ginseng. The phenotypic plasticity index (PPI) between 25-year-old F. Ginseng and W. Ginseng was minimum, indicating that F. Ginseng mainly adapts to changes in growth by regulating morphology and allocating biomass (Table 1). Our results are in agreement with those of Huang et al. (2012).

### Differences between relative abundance of F. Ginseng and W. Ginseng

Proteins, the expression of which could explain the life characteristics of organisms with a specified status, ultimately control biological processes. There is a negative correlation between the abundance of protein and the rate of evolution (Wolf et al., 2010). The proteins with high and intermediate abundance with central importance in cells may reflect the genetic relationships between different species to some extent (Beck et al., 2011; Zhong et al., 2012). We compared the proteomic profiles of F. Ginseng and W. Ginseng to explore similar characteristics between the two ginseng types (Figure S1). The experimental workflow is shown in Figure S2.

**Figure 3:**
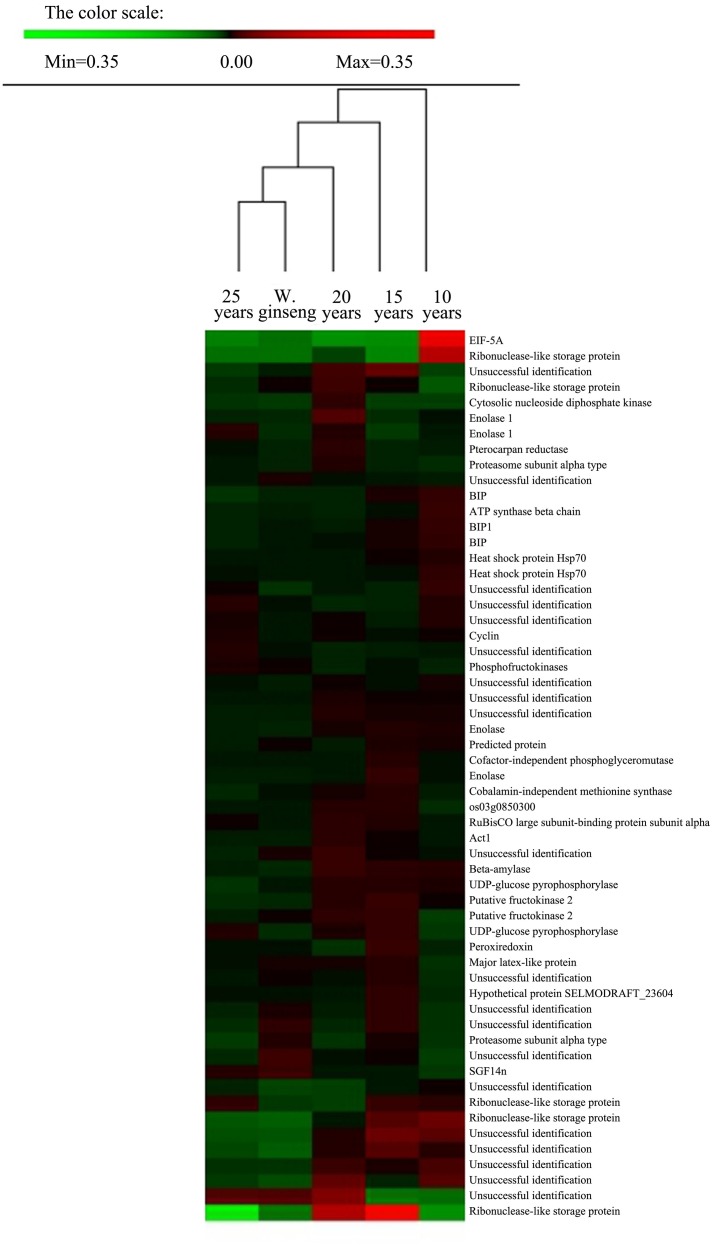
**Cluster analysis of spots showing differences in relative volumes among F. Ginseng in different growth years and W. Ginseng**. Two-way hierarchical clustering analysis of the 57 spots showed at least a 1.5-fold change in the relative spot volumes (ANOVA, *P* < 0.01) among the five samples. The clustering analysis was performed with PermutMatrix graphical interface after Z-score normalization of the averages of relative spot values (*n* = 6). Pearson's distance and Ward's algorithm were used for the analysis.

We analyzed the proteins present at different levels between F. Ginseng and W. Ginseng by hierarchical clustering analysis using PermutMatrix software. We compared the protein levels in the five samples including 10-, 15-, 20-, and 25-year-old of F. Ginseng and W. Ginseng (Figure 3). The clustering of the differentially accumulated proteins revealed two major clusters as seen from the dates of the columns. The 10- and 15-year-old F. Ginsengs clustered into one group preferentially, with the next level clustering with the 20-year-old ginseng. These findings indicate that the 10-, 15-, and 20-year-old ginseng plants are more closely related on the protein level than the other plants tested here. Notably, 25-year-old ginseng and wild ginseng clustered into one group. This finding shows that the morphological and physiological parameters of F. Ginseng and W. Ginseng are reshaped over the years of growth, and these changes are regulated by protein abundance. On the protein level, this result validates the traditional view that the morphology of older F. Ginseng becomes closer to W. Ginseng.

### 2DE analyses of differential expression protein

**Figure 4:**
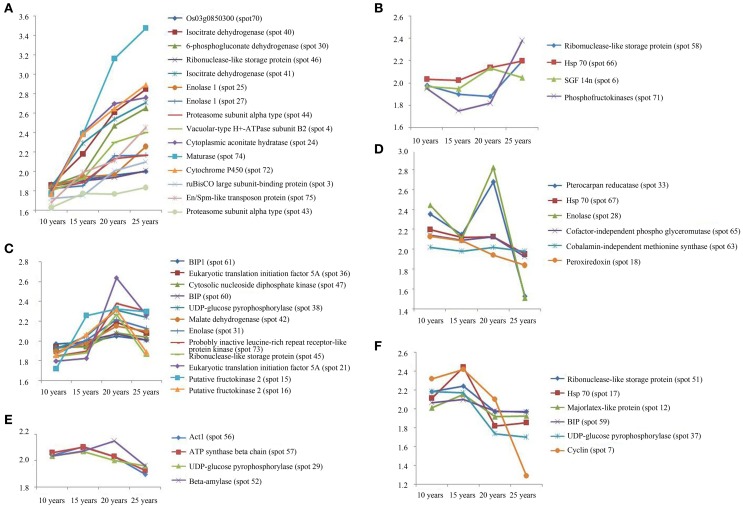
**The clusters of the abundance of different expressed proteins from F. Ginseng in different growth years by MALDI-TOF/TOF-MS/MS**. The up-regulated proteins in cluster **(A–C)**; The down-regulated proteins in cluster **(D–F)**.

The protein expression patterns of F. Ginseng in different growth years were analyzed by 2DE. We identified 47 proteins as continuously changing based on their accumulation patterns (Figure S1D). Among these proteins, 31 were up-regulated, and 16 were down-regulated. We divided the mass spectral data into six groups with clustering analysis (Figure 4, Table S2). We further grouped the up-regulated proteins into three clusters. Proteins expressed at continuously increasing levels with years of growth of F. Ginseng were grouped in cluster A. Cluster B proteins were reduced in abundance during the 15th and 20th year of growth. Proteins that first increased in abundance and then decreased were placed in cluster C. A total of 16 proteins decreased in abundance during the 25th year of F. Ginseng growth compared with other growth periods. We defined these as growth-related proteins because they either re-accumulated in 20-year-old F. Ginseng (after first decreasing in abundance in 15-year-old plants, cluster D) or increased in abundance in 15-year-old plants (clusters E and F).

### iTRAQ analyses of differentially expressed protein

**Figure 5:**
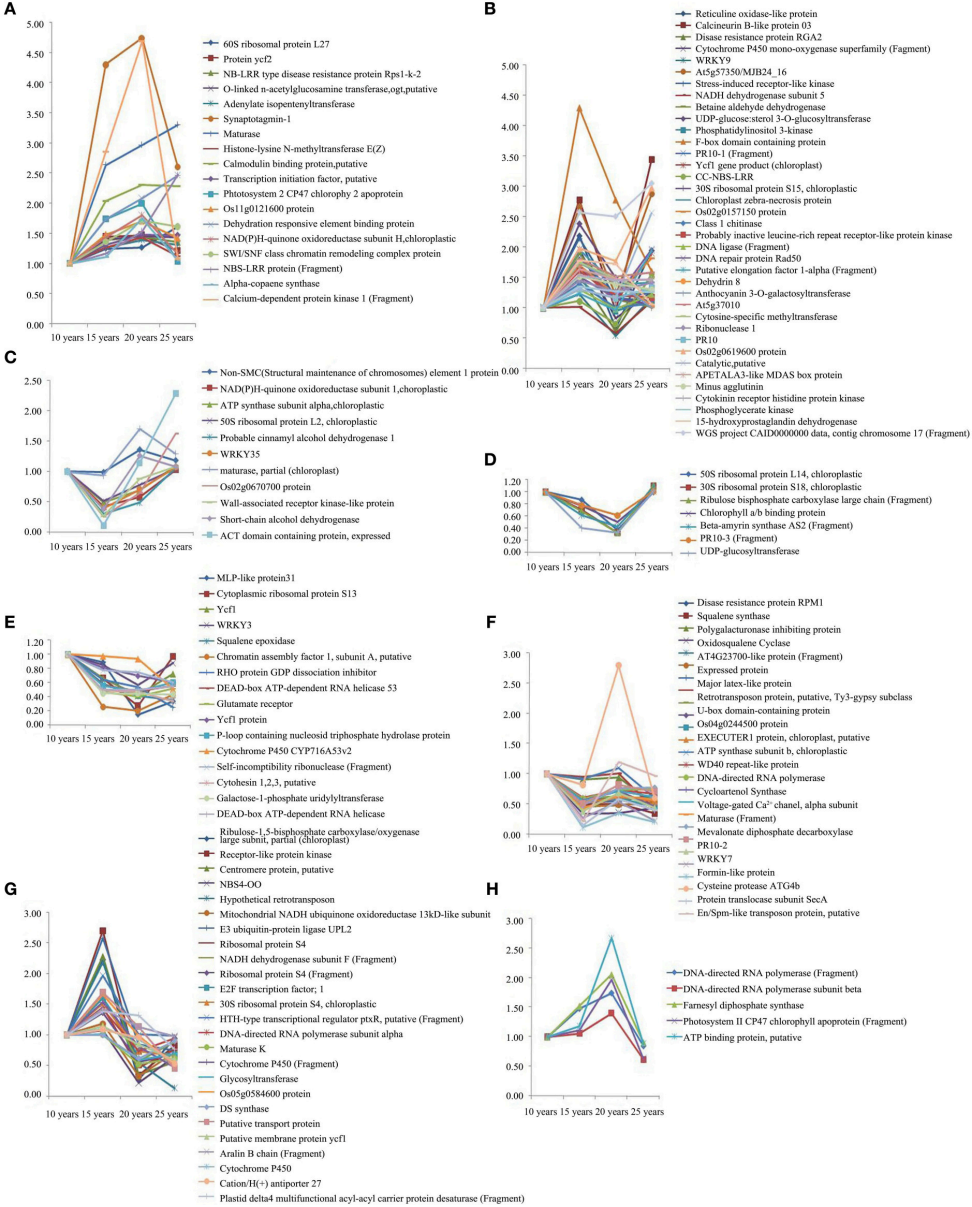
**The clusters of abundance of different expressed proteins from F. Ginseng in different growth years by iTRAQ**. The up-regulated proteins in cluster **(A–D)**; The down-regulated proteins in cluster **(E–H)**.

We used 2DE to analyze the proteins with high and intermediate abundance. However, the low-abundance proteins were difficult to detect; therefore, we used iTRAQ for the quantitative analysis of the differentially expressed proteins of F. Ginseng in different growth years to corroborate and supplement the 2DE analysis data. We used three biological replicates for each condition to identify 145 differentially expressed proteins. This number excluded the unknown and predicted proteins. Our results revealed a spectrum of different temporal expression patterns (Figure 5, Table S3), ranging from proteins up-regulated primarily during the latter stages of the time course (clusters A–D) to proteins down-regulated primarily during the latter phases of the time course (clusters E–H). Proteins in the growth process category were statistically over-represented in clusters A–D and under-represented in clusters E–H. The proteins increased in abundance in 15 years old in clusters B and G, and in 20 years old in clusters A, F, and H in the growth process category. The abundance of proteins in clusters D and E exhibited no significant changes and returned to control levels in 25-year-old plants. Clusters A, B, and G also included most of the highly induced proteins (>2-fold increase compared with the 15-year-old plants). These changes in protein abundance demonstrate that the growth and development of F. Ginseng could be controlled by changes in the expression of these differentially expressed proteins (Ma et al., 2013).

### Functional analysis of identified proteins

**Figure 6:**
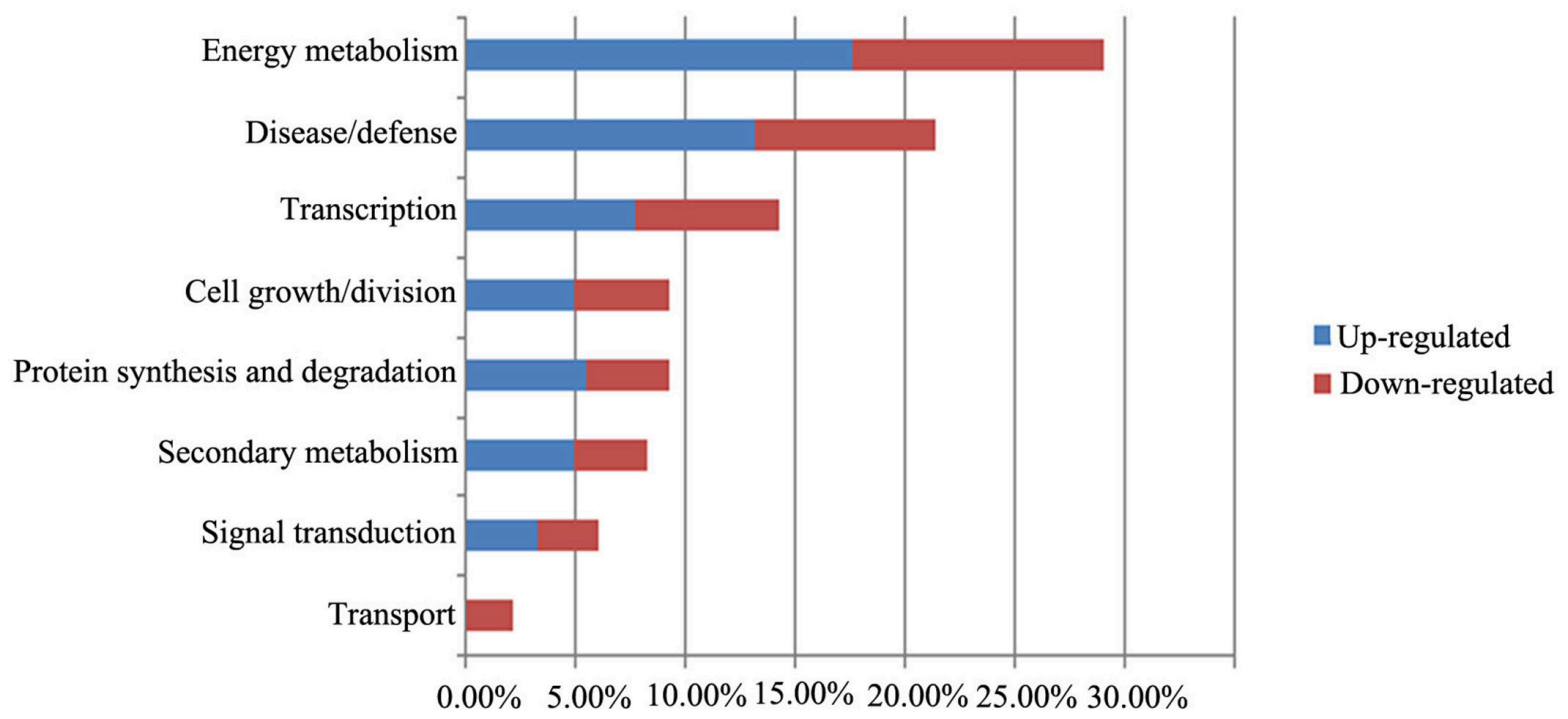
**Functional analyses of differentially expressed proteins in the biological process**. Percentage distributions of the GO terms were calculated by iProClass GO tool in PIR database.

We grouped the proteins identified by 2DE and iTRAQ according to their biological functions (Figure 6), which were determined using the iProClass Gene Ontology (GO) analysis tool in the Protein Information Resource (PIR) database (http://pir.georgetown.edu/). The GO classification will help to identify the metabolic events associated with the growth of F. Ginseng.

The main biological functions were: energy metabolism (29%), disease/defense (21%), transcription related(14%), cell growth/division (9%), protein synthesis and degradation (9%), and secondary metabolism (8%). In addition, some proteins were categorized in unknown biological processes.

### Analysis of differentially expressed enzymes activity

**Figure 7:**
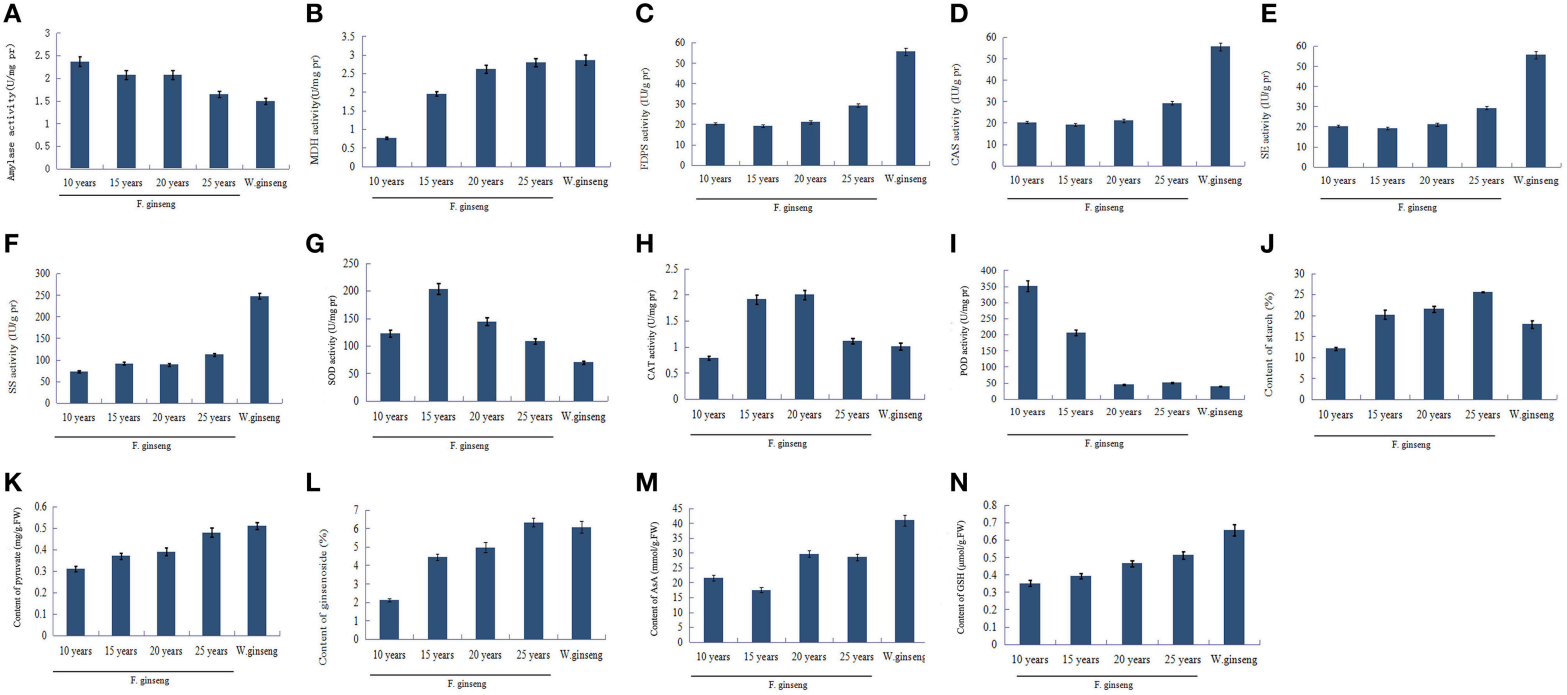
**The key enzyme activities and metabolite contents of F. Ginseng in different growth years**. Bars indicate ±SD. **(A)**, Amylase activity; **(B)**, MDH activity; **(C)**, FDPS activity; **(D)**, CAS activity; **(E)**, SE activity; **(F)**, SS activity; **(G)**, SOD activity; **(H)**, CAT activity; **(I)**, POD activity; **(J)**, Starch contents; **(K)**, Pyruvic acid contents; **(L)**, Saponin content; **(M)**, AsA contents; **(N)**, GSH contents.

The level of activity is positively correlated with the enzyme protein abundance (Yang et al., 2013). To validate the proteomics data, two enzymes involved in glycometabolism, four enzymes involved in ginsenosides biosynthesis, and three enzymes involved in ROS scavenging between F. Ginseng and W. Ginseng were selected for activity analysis (Figure 7). The activities of AMY (Figure 7A) were lower, whereas the activities of MDH (Figure 7B) were higher in older F. Ginseng. The activities of FDPS (Figure 7C), CAS (Figure 7D), SE (Figure 7E), and SS (Figure 7F) increased with growth years of F. Ginseng. The activities of SOD (Figure 7G) and CAT (Figure 7H) changed dynamically in a parabolic pattern, and POD (Figure 7I) showed a downward trend over the course of F. Ginseng growth. These results concur with the protein profiles of the 2DE and iTRAQ analysis. Moreover, the changes in enzyme activity of F. Ginseng (with growth time) should be close to that of W. Ginseng.

### Changes in metabolite(s) content

Metabolite synthesis is influenced by the activities of the enzymes that regulate the metabolic pathways. In this study, we found that the starch (Figure 7J) content of F. Ginseng increased with increase in growth years, which showed a negative correlation with the activity of amylase. Pyruvate (Figure 7K) is an intermediate metabolite of glucose metabolism and its content increased with growth years indicating a gradually active glucose metabolism. The content of ginsenoside (Figure 7L), as well as the activities of the enzymes in ginsenoside synthesis also increased with growth years. Moreover, antioxidants such as AsA (Figure 7M) and GSH (Figure 7N) accumulated to enhance the antioxidant capacity of F. Ginseng. The above results indicate that F. Ginseng becomes increasingly similar to wild ginseng as growth years increase.

### The expression analysis of Grx and PR5 at the mRNA level

**Figure 8:**
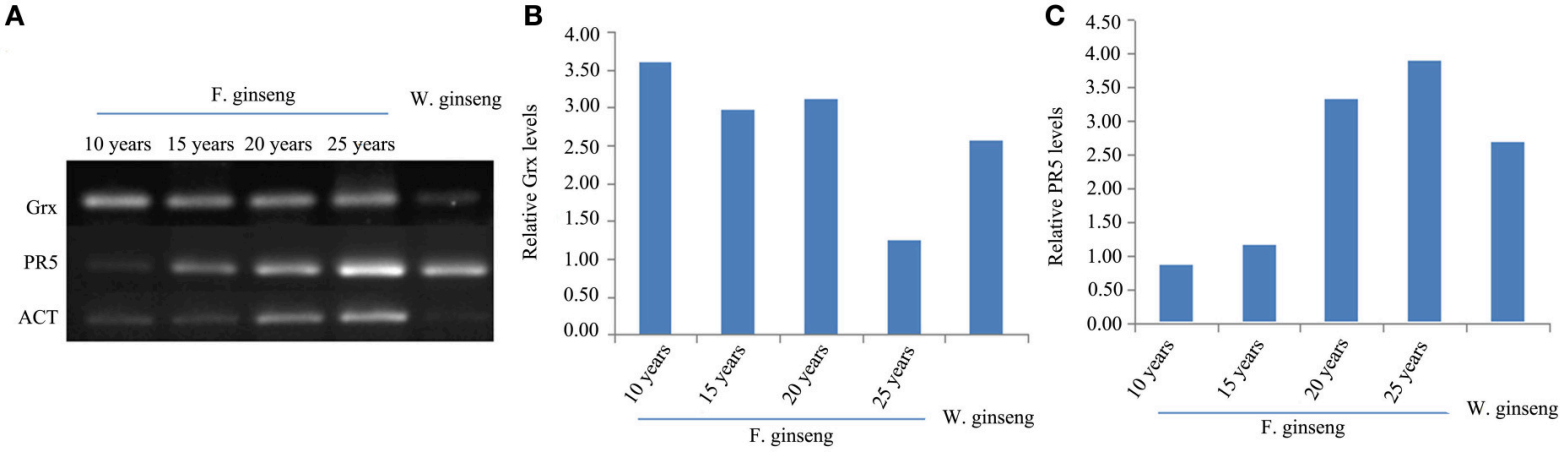
**Expressions (A) and Relative levels of Grx gene (B) and PR5 gene (C) of F. Ginsengs in different growth years and W. Ginseng**. Actin gene from ginseng was used as a control.

In order to assess the correlation of expression levels between mRNA and protein, we did a semi-quantitative PCR for the defense genes, Grx and PR5 (Figure 8). The expression of Grx gene was down-regulated in response to stress year after year. Conversely, the expression of PR5 was up-regulated year after year. Therefore, the expression of these proteins, in response to stress, is regulated at the transcriptional level in growing F. Ginseng.

## Discussion

### Active energy metabolism for growth and development

The growth and development of F. Ginseng require active energy metabolism to produce the necessary material and energy. Our data shows that the largest category of proteins in F. Ginseng is associated with energy metabolism. This category primarily includes proteins involved in starch metabolism, glycolysis, the pentose phosphate pathway, and the tricarboxylic acid cycle.

Fructokinases—that induce starch synthesis—were overexpressed over the time course of F. Ginseng growth (spot 15, 16) (Figure 4 clusters C). Amylase (spot 58) (Figure 4 clusters B), which reduce starch degradation, were down-regulated over time. Starch content (Figure 7J) in F. Ginseng was positively correlated with the fructokinase expression and negatively correlated with the amylase activity (Figure 7A); thus, corroborating the study of Yang et al. (2001). F. Ginseng consumes many nutrients in the early growth period to provide enough material and energy for morphogenesis and development. Starch accumulates as F. Ginseng matures, and the same trend is observed in tomato, maize endosperm, and Arabidopsis seeds (Schaffer and Petreikov, 1997; Angeles-Núñez and Tiessen, 2011; Spielbauer et al., 2013). The starch accumulation of W. Ginseng less than F. Ginseng probably could due to a lack of nutritional growth environment.

Glycolysis is active as the downstream pathway of starch metabolism. Enolase (spot 25, 27, 31) (Figure 4 clusters A and C), Glucose-6-phosphate dehydrogenase (6PGDH, spot 30) (Figure 4 clusters A), and glyceric acid phosphate mutase, which is involved in glycolysis, was up-regulated in mature F. Ginseng, providing energy for the growth and development of F. Ginseng. Pyruvic acid, as the end product of glycolysis, increased with an increase in the growth of F. Ginseng (Figure 7K). Thereafter, the intermediate products of active glycolysis promote other energy metabolic pathways (Gómez et al., 2012). MDH (spot 42) (Figure 4 clusters C. Figure 7B), aconitic acid hydratase and isocitrate dehydrogenase (spots 40 and 41) (Figure 4 clusters A) related to Krebs cycle and pentose phosphate pathway accumulated from year to year. Energy metabolism of the older F. Ginseng was active to provide a large number of ATPs for the growth and metabolism, and this trend approached that of W. Ginseng year after year.

In addition to the energy metabolism pathways discussed above, we also observed an increase in the expression of related proteins in F. Ginseng. This suggests that the energy metabolism increases as F. Ginseng matures, providing energy to support its morphological changes. This result was consistent with garden ginseng growth, wherein active energy metabolism promotes root enlargement and weight gain (Ma et al., 2013). Moreover, large amounts of precursor substances are synthesized to promote effective substance biosynthesis to improve the quality of F. Ginseng medicinal components.

### Ginsenosides biosynthesis promotes the medicinal properties of F. Ginseng

Ginsenoside—active ingredients of ginseng—are a class of natural product steroid glycosides and triterpene saponins. Ginsenosides have a long history of use in traditional medicine such as anti-tumor and neurotrophic activity, and enhancement of immunity (Rudakewich et al., 2001; Zhang et al., 2008; Song et al., 2010). Studies have shown that the ginsenoside content continues to increase along with the age of ginseng (Soldati and Tanaka, 1984). An increase in the content of ginsenosides is directly related to an increase in their pharmacological effect (Lin et al., 2010). The synthesis and effects of ginsenosides present positive correlation in ginseng growth.

We identified enzymes involved in the biosynthesis of ginsenosides in our study. These enzymes (proteins) were differentially expressed in F. Ginseng in different growth years. FDPS (Figure 5 clusters H, Figure 7C), CAS (Figure 5 clusters F, Figure 7D), SE (Figure 5 clusters E, Figure 7E), and SS (Figure 5 clusters F, Figure 7F) were up-regulated to increase the synthesis ability of the F. Ginseng ginsenoside. Mature F. Ginseng plants had higher ginsenoside content (Figure 7L) as compared with their younger counterparts. Our results suggest F. Ginseng that ginsenoside synthesis in F. Ginseng increases every year to enhance its medicinal properties and become more like W. Ginseng.

### Scavenging ROS to maintain redox balance

Plants are vulnerable to growth environmental stresses, which result in excessive accumulation of ROS, causing cell damage. ROS scavenging enzymes such as peroxiredoxin (spot 18) (Figure 4 clusters D), SOD (Figure 7G), CAT (Figure 7H), and Grx gene (Figures 8A,B; Ding et al., 2010) were up-regulated in the early growth stage and down-regulated in the mature stage. Thus, F. Ginseng activated the antioxidant defense system to cope with the oxidative stress by maintaining a relatively stable level of ROS (Arbona and Gómez-Cadenas, 2012).

A key enzyme 6PGDH of the pentose phosphate pathway, is expressed at high levels in response to drought, low temperature and high salt stress (Airaki et al., 2012; Signorelli et al., 2013; Wang et al., 2013). Our results show up-regulated 6PGDH possibly to activate the pentose phosphate pathway and produce large amounts of reducing substances (NADPH), which remove active oxygen via the AsA-GSH cycle. We tested the AsA (Figure 7M) and GSH levels (Figure 7N), which increased with F. Ginseng growth. Our results showed that older F. Ginseng exhibitsW. Ginseng enhanced stress tolerance (similar to W. Ginseng) to maintain redox homeostasis within the cells.

### Enhanced expression of resistance genes and proteins to ensure health

Plant disease resistance is accomplished by temporal and spatial changes in gene expression that cause changes in the physiological and biochemical reactions to aid in resistance to pathogens. The NBS-LRR region is an important component of the disease resistance genes in the plant immune system (Nimchuk et al., 2003; Belkhadir et al., 2004), exists in DNA fragments of certain conservative domains in plant genomes, such as mosaic virus resistance genes (N gene) in Soybean and potato (Bakker et al., 2011; Zhang et al., 2012). We identified NBS-LRR (Figure 5 clusters A) and CC-NBS-LRR (Figure 5 clusters B) homologs in F. Ginseng, which were up-regulated over the growth period, and could improve the disease resistance and maintain the health of F. Ginseng. Meanwhile, the NBS (nucleotide binding site) participates in hydrolyzing ATP and releasing signals (Ade et al., 2007) such as ATPase (Figure 4 clusters E) and RHO protein GDP dissociation inhibitor (Rho GDI) (Figure 5 clusters E), which are involved in signal transduction of stress response (Wong et al., 2004).

Furthermore, the expression of resistance genes can result in the production and accumulation of the pathogenesis-related protein (PR), which can be induced by components of the plant's self-defense mechanism. We identified PR10 (Figure 5 clusters D and F) by iTRAQ and PR5 gene (Figures 8A,C) was assayed by semi-quantitative RT-PCR. They were up-regulated in response to stress year after year. We observed the expression of several of these proteins, including major latex-like protein (Figure 4 clusters F and Figure 5 clusters F), ribonuclease (Figure 5 clusters B), and POD. The up-regulation of these pathogenesis-related proteins could increase the adaptive ability of roots to adverse growth environments, which may be part of the defense mechanism of F. Ginseng (Sun et al., 2010).

### Transcriptional regulation for growth

DNA methylation, histone modification, and chromatin reorganization are some of the mechanisms that regulate transcription (Turner, 2009). We observed upregulated expression of cytosine-specific methyltransferase (Figure 5 clusters B) and histone-lysine N-methyltransferase (Figure 5 clusters A), which participate in DNA methylation and histone methylation. Both of these enzymes have a synergistic effect (Fuks et al., 2003) to regulate the response of F. Ginseng to various environmental stresses (Sharma et al., 2009; Bilichak et al., 2012; Chervona and Costa, 2012). Moreover, transposons and retrotransposons have DNA methylation sites, which are methylated and silenced to protect the host genome (Cam et al., 2008). The upregulated retrotransposon (Figure 5 clusters F and G) and En/Spm-like transposon protein (Figure 4 clusters A and Figure 5 clusters F) indicate their active involvement in inhibiting improper transcription, insertion mutations, and recombination in the F. Ginseng genome.

SWI/SNF (SWItch/Sucrose NonFermentable) is a chromatin remodeling complex that helps in DNA packaging. In our study, both chromatin remodeling complex protein (Figure 5 clusters A; Reyes, 2014) and chromatin assembly factor (Figure 5 clusters E; Margueron and Reinberg, 2010) involved in SWI/SNF were differentially expressed over the course of F. Ginseng growth. These proteins maintain the right chromatin structure to promote the appropriate combination of transcription factors and basic transcription elements that help in the expression of resistance genes. We also identified some transcription factors that are overexpressed in the growth process. Eukaryotic translation initiation factor 5A (spots 21, 36; Figure 4 clusters C) involved in resistance to oxidative and osmotic stress (Xu et al., 2011). WRKYs (Figure 5 clusters B, C, E and F) participate in the regulation of related gene expression in response to changes in the growth environment (Mondini et al., 2012). These changes on transcription regulation in different growth stages of F. Ginseng provide a mechanism to adapt to the rapidly changing growth environment as well as to W. Ginseng.

## Conclusion

**Figure 9:**
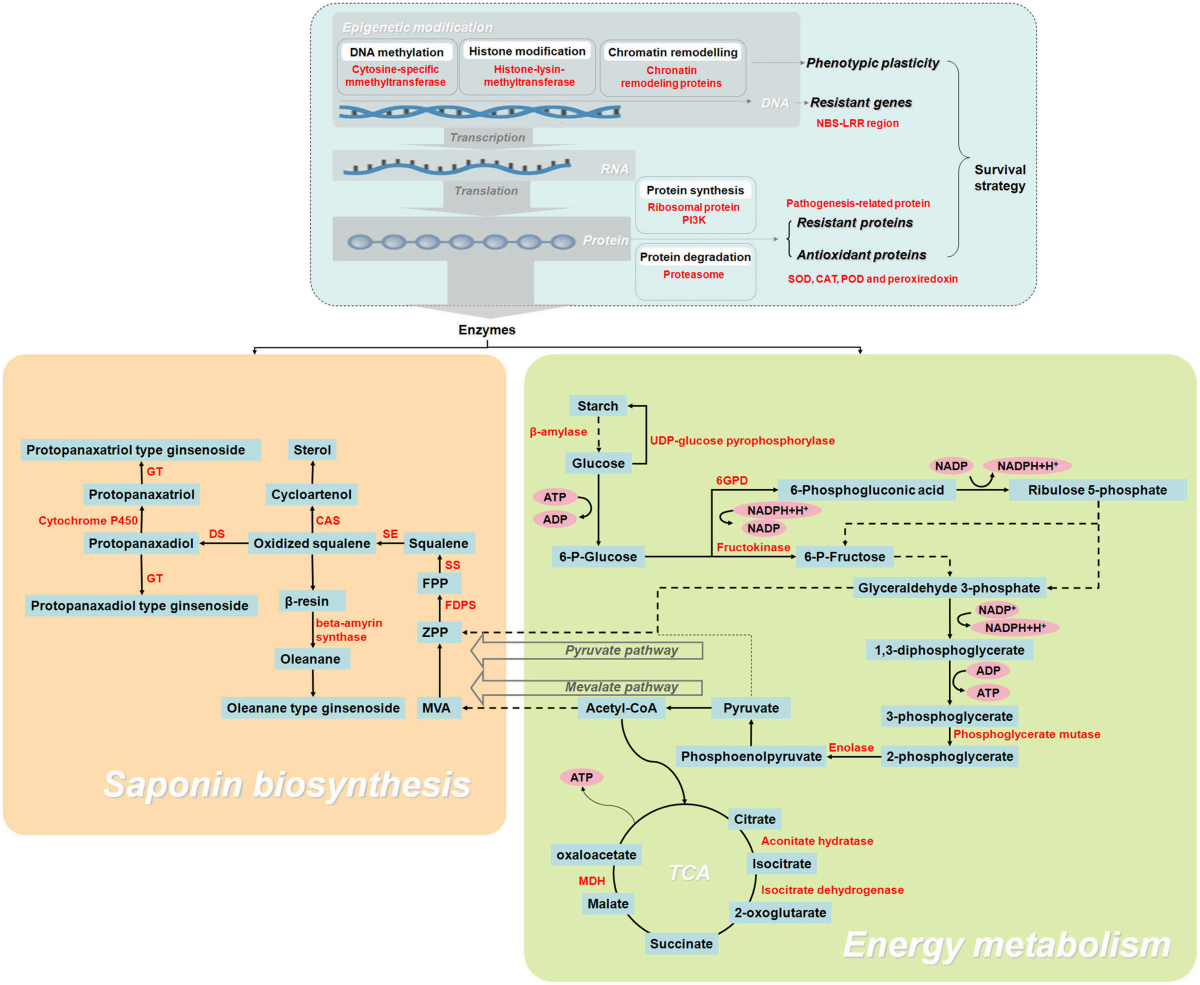
**Schematic overview of the enzymes and metabolites involved in the growth and development of F. Ginseng, reveals its growth and ginsenoside biosynthesis**.

To our knowledge, this work is the first large-scale proteomic investigation F. Ginsengof F. Ginseng growth. As F. Ginsengs grow, their morphological characteristics and metabolism become comparable to that of W. Ginseng. The changes in protein abundance revealed that 25-year-old F. Ginseng is the most similar to W. Ginseng. Proteins identified from 192 spots and discussed in relation to F. Ginseng highlighted the metabolic changes occurring during the growth, with particular regard to proteins associated with energy metabolism, ginsenosides biosynthesis, and antioxidation (Figure 9). Therefore, 25-year-old F. Ginseng plants have better external morphological and medicinal effect, which is similar to wild ginseng, than their younger counterparts. This F. Ginseng study improves our understanding of the medicinal properties of F. Ginseng and provides a future direction for engineering new varieties with higher medicinal value.

## Author contributions

LS, DZ: Substantial contributions to the conception or design of the work. RM, XC, BM, GC, MW: the acquisition, analysis and interpretation of data for the work. RM, LS, XC: Drafting the work or revising it critically for important intellectual content. RM, LS: Final approval of the version to be published. All authors agreed to be accountable for all aspects of the work in ensuring that questions related to the accuracy or integrity of any part of the work are appropriately investigated and resolved.

### Conflict of interest statement

The authors declare that the research was conducted in the absence of any commercial or financial relationships that could be construed as a potential conflict of interest.

## Supplementary material

Figure S1 **Comparison of F. Ginseng proteome pattern in different growth years, which were 10 years old (A), 15 years old (B), 20 years old (C), and 25 years old (D) respectively, and W. Ginseng (E)**. The arrows indicate the spot numbers corresponding to the differentially accumulated spots among F. Ginsengs of different ages. Statistics on the indicated spots can complete identification data in Supporting Information Table S2.

Figure S2 **Experimental workflow of proteomics analysis**.

Table S1 **Oligonucleotide sequences of primers used for semiquantitative RT-PCR**.

Table S2 **Identification of differentially expressed proteins from F. Ginseng in different growth years by MALDI-TOF/TOF-MS/MS**. ^a^Clusters, The clusters of abundance of differentially expressed proteins in Figure 4; ^b^Spot no, spot numbers correspond with 2-DE gel as shown in Figure S1D; ^c^Accession number in NCBI database. ^d^Pep. count, Number of matched peptides.

Table S3 **Identification of different expressed proteins from F. Ginseng in different growth years by iTRAQ**. Expression differences were defined as protein abundance ratios greater than 1.4 or less than 0.6. ^a^Clusters, The clusters of abundance of differentially expressed proteins in Figure 5; ^b^Accession number in NCBI database.^c^15/10, the ratios of protein abundances between 15 and 10 years of F. Ginseng; ^d^20/10, the ratios of protein abundances between 20 and 10 years of F. Ginseng; ^e^25/10, the ratios of protein abundances between 25 and 10 years of F. Ginseng.
