# Supplementary material for: Proteomic Analyses Provide Novel Insights into Plant Growth and Ginsenoside Biosynthesis in Forest Cultivated Panax ginseng (F. Ginseng)
Source: Front Plant Sci. 2016 Jan 26;7:1. doi: 10.3389/fpls.2016.00001 (PMC4726751; doi:10.3389/fpls.2016.00001)
Supplement: Figure S1 — Comparison of F. Ginseng proteome pattern in different growth years, which were 10 years old (A), 15 years old (B), 20 years old (C), and 25 years old (D) respectively, and W. Ginseng (E). The arrows indicate the spot numbers corresponding to the differentially accumulated spots among F. Ginsengs of different ages. Statistics on the indicated spots can complete identification data in Supporting Information Table S2. [file Image1.PDF]

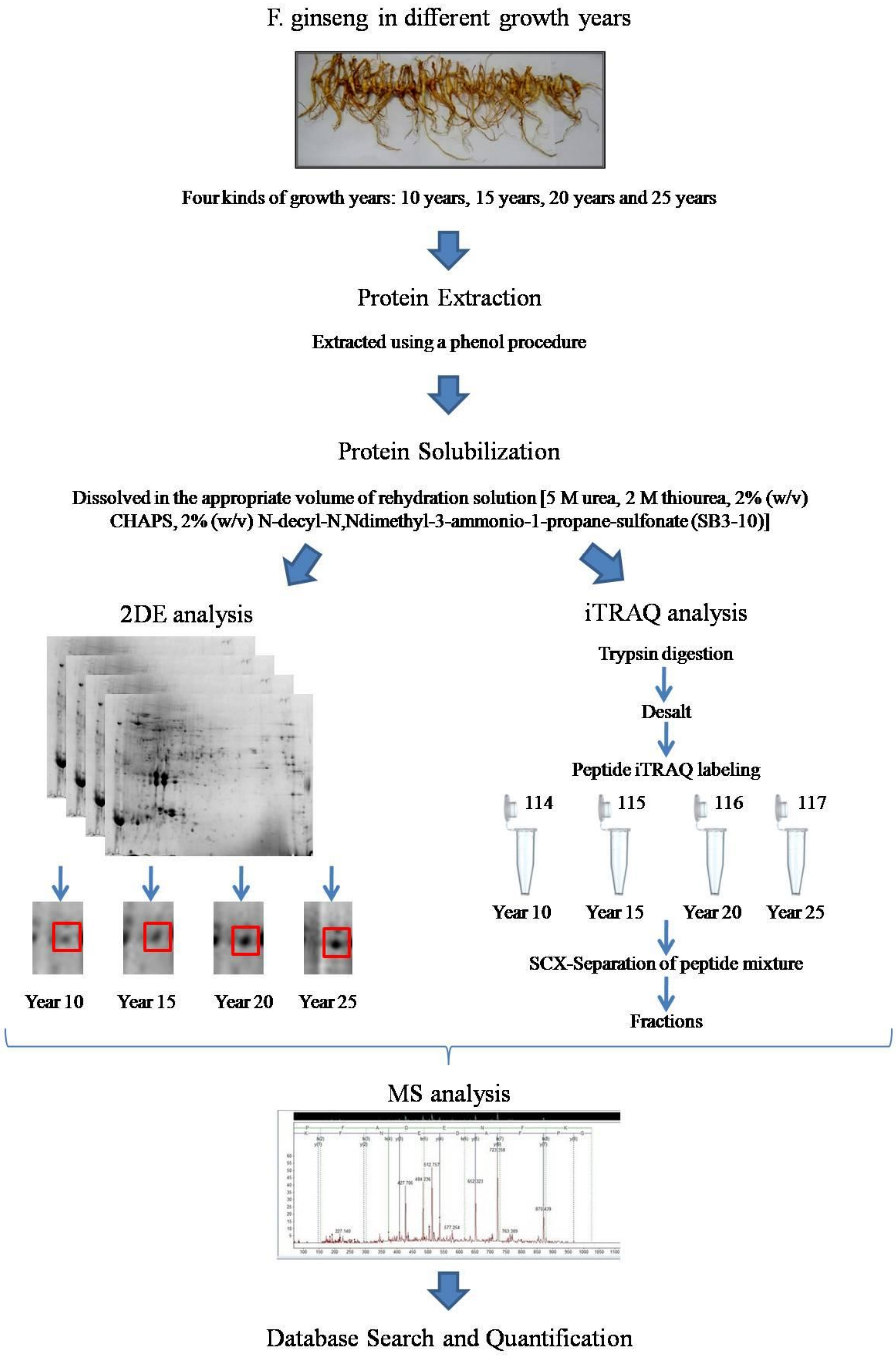
F. ginseng in different growth years
Four kinds of growth years: 10 years, 15 years, 20 years and 25 years
Protein Extraction
Extracted using a phenol procedure
Protein Solubilization
Dissolved in the appropriate volume of rehydration solution [5 M urea, 2 M thiourea, 2% (w/v) CHAPS, 2% (w/v) N-decyl-N,Ndimethyl-3-ammonio-1-propane-sulfonate (SB3-10)]
2DE analysis
Year 10
Year 15
Year 20
Year 25
iTRAQ analysis
Trypsin digestion
Desalt
Peptide iTRAQ labeling
114
115
116
117
Year 10
Year 15
Year 20
Year 25
SCX-Separation of peptide mixture
Fractions
MS analysis
Database Search and Quantification
