# Supplementary figures and images for: Proteomic Analyses Provide Novel Insights into Plant Growth and Ginsenoside Biosynthesis in Forest Cultivated Panax ginseng (F. Ginseng)
Source: Front Plant Sci. 2016 Jan 26;7:1. doi: 10.3389/fpls.2016.00001 (PMC4726751; doi:10.3389/fpls.2016.00001)

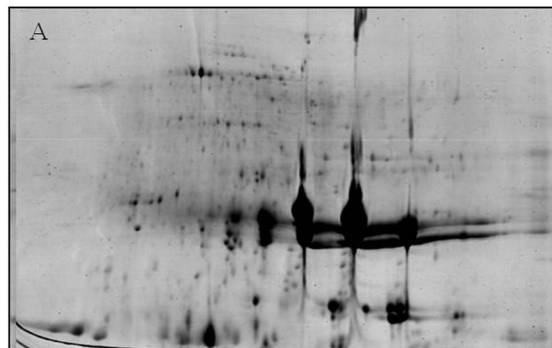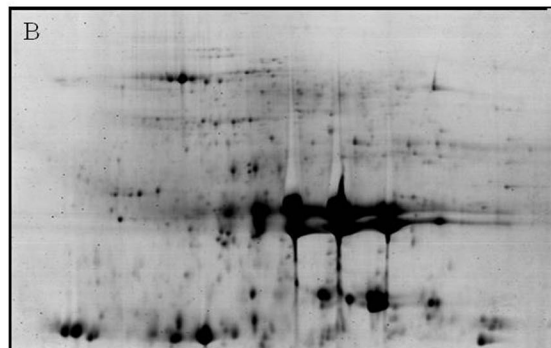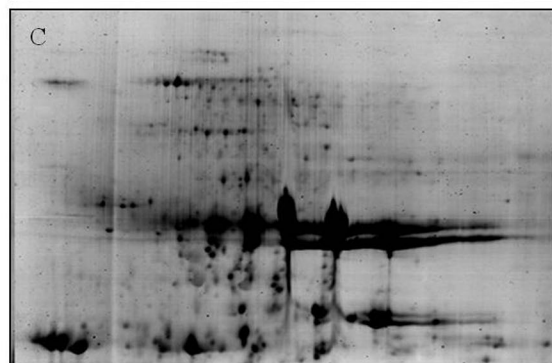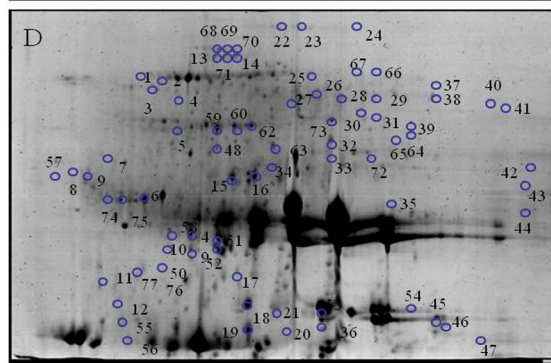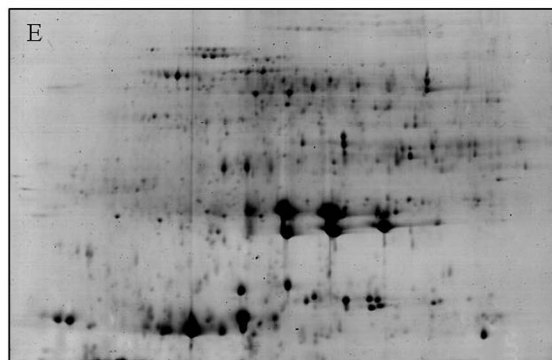

Supplement: Figure S2 — Experimental workflow of proteomics analysis. [file Image2.PDF]
