# Supplementary material for: Proteomic Analyses Provide Novel Insights into Plant Growth and Ginsenoside Biosynthesis in Forest Cultivated Panax ginseng (F. Ginseng)
Source: Front Plant Sci. 2016 Jan 26;7:1. doi: 10.3389/fpls.2016.00001 (PMC4726751; doi:10.3389/fpls.2016.00001)
Supplement: Table S1 — Oligonucleotide sequences of primers used for semiquantitative RT-PCR. [file Table1.PDF]

Table S1. Oligonucleotide sequences of primers used for semiquantitative RT-PCR.

Primer name	Primer sequences (5’-3’)	Reference
PR5F	5'-AAC CGA CTG CAA CTT CGA CT-3'	Yu-Jin 2009
PR5R	5'-GGC ACA TTA AAC CCA TCC AC-3'	Yu-Jin 2009
GrxF	5’-GCA AAG GAG CTG GTT TCA TC-3’	Yu-Jin 2008
GrxR	5’-CAT GGT GCA ATT AAC CCA CA-3’	Yu-Jin 2008
ACTF	5’- CGT GAT CTT ACA GAT AGC TTG ATG A -3’	Yu-Jin 2008
ACTR	5’- AGA GAA GCT AAG ATT GAT CCT CC -3’	Yu-Jin 2008
