# Supplementary material for: Proteomic Analyses Provide Novel Insights into Plant Growth and Ginsenoside Biosynthesis in Forest Cultivated Panax ginseng (F. Ginseng)
Source: Front Plant Sci. 2016 Jan 26;7:1. doi: 10.3389/fpls.2016.00001 (PMC4726751; doi:10.3389/fpls.2016.00001)
Supplement: Table S2 — Identification of differentially expressed proteins from F. Ginseng in different growth years by MALDI-TOF/TOF-MS/MS. aClusters, The clusters of abundance of differentially expressed proteins in Figure 4; bSpot no, spot numbers correspond with 2-DE gel as shown in Figure S1D; cAccession number in NCBI database. dPep. count, Number of matched peptides. [file Table2.PDF]

Table S2.

clusters [a]	Spot no [b]	Accession no.[c]	Protein name	Species	Mr(kDa)/pI	Pep. count [d]	Sequence
Energy metabolism							
A	3	gi|1351030	RuBisCO large subunit-binding protein subunit alpha, chloroplastic	Brassica napus (Rape)	57656.76/4.84	13	K.APGFGER.R<br>K.VVNDGVTIAR.A<br>K.LLVEFENAR.V<br>K.LLVEFENAR.V<br>K.VGAATETELEDR.K<br>R.GYISPQFVTNPEK.L<br>R.GYISPQFVTNPEK.L<br>K.ELSETDSVYDSEK.L<br>R.AIELPDAMENAGAALIR.E<br>R.AIELPDAMENAGAALIR.E<br>R.AIELPDAMENAGAALIR.E + Oxidation (M)<br>K.DSTTLIADAASKDELQAR.I<br>K.DSTTLIADAASKDELQAR.I
A	4	gi|62321641	vacuolar-type H+-ATPase subunit B2	Arabidopsis thalian	54304.74/5.03	15	R.EEVPGRR.G<br>K.TLDQFYSR.D<br>K.TLDQFYSR.D<br>K.FVAQGAYDTR.N<br>K.FVAQGAYDTR.N

							R.KFVAQGAYDTR.N<br>R.KFVAQGAYDTR.N<br>R.QIYPPINVLPSLSR.L<br>R.QIYPPINVLPSLSR.L<br>R.NIFQSLDLAWTLLR.I<br>R.NIFQSLDLAWTLLR.I<br>R.VTLFLNLANDPTIER.I<br>R.VTLFLNLANDPTIER.I<br>R.GYPGYMYTDLATIYER.A<br>R.GYPGYMYTDLATIYER.A
C	15	gi|33329200	putative fructokinase 2	Petunia integrifolia subsp. inflata	34953/5.20	10	R.EFMFYR.N<br>R.EFMFYR.N + Oxidation (M)<br>R.EFMFYR.N + Oxidation (M)<br>R.TALAFVTLR.A<br>R.TALAFVTLR.A<br>R.LPLWPSAEEAR.K<br>R.LPLWPSAEEAR.K<br>R.LPLWPSAEEARK.Q<br>K.EAGALLSYDPNLR.L<br>K.EAGALLSYDPNLR.L
C	16	gi|33329200	putative fructokinase 2	Petunia integrifolia subsp. inflata	34953/5.20	10	R.EFMFYR.N<br>R.EFMFYR.N + Oxidation (M)<br>R.EFMFYR.N + Oxidation (M)<br>R.TALAFVTLR.A<br>R.TALAFVTLR.A<br>R.LPLWPSAEEAR.K

							R.LPLWPSAEEAR.K<br>R.LPLWPSAEEARK.Q<br>K.EAGALLSYDPNLR.L<br>K.EAGALLSYDPNLR.L
A	24	gi|4586021	cytoplasmic aconitate hydratase	Arabidopsis thaliana	108132.71 /6.72	17	R.VHPLTR.A<br>K.LPYSIR.I<br>R.GTFANIR.I<br>R.ILLESAIR.N<br>K.DFNSYGSR.R<br>K.DFNSYGSR.R<br>R.KDFNSYGSR.R<br>K.DFNSYGSRR.G<br>K.QVEIPFKPAR.V<br>K.QVEIPFKPAR.V<br>K.FYSLPALNDPR.V<br>K.FYSLPALNDPR.V<br>R.ANNMFVDYNEPQQDR.V<br>R.ANNMFVDYNEPQQDR.V<br>R.ANNMFVDYNEPQQDR.V + Oxidation (M)<br>K.INPLVPVDLVIDHSVQVDVAR.S<br>K.INPLVPVDLVIDHSVQVDVAR.S
A	25	gi|14423688	Enolase 1	Hevea brasiliensis (Para rubber tree)	47801/5.57	7	R.QIFDSR.G<br>K.YNQLLR.I<br>K.EAMKMGAEVYHHLK.S + 2 Oxidation<br>R.AAVPSGASTGIYEALELR.D<br>R.AAVPSGASTGIYEALELR.D

							R.IEEELGSEAVYAGANFR.K<br>R.IEEELGSEAVYAGANFR.K
A	27	gi|14423688	Enolase 1	Hevea brasiliensis (Para rubber tree)	47800.52/5.57	15	R.QIFDSR.G<br>K.YNQLLR.I<br>R.AGWGVMASHR.S<br>R.AGWGVMASHR.S<br>R.AGWGVMASHR.S + Oxidation (M)<br>R.AGWGVMASHR.S + Oxidation (M)<br>K.VNQIGSVTESIEAVK.M<br>R.AAVPSGASTGIYEALELR.D<br>R.AAVPSGASTGIYEALELR.D<br>R.IEEELGSEAVYAGANFR.K<br>R.IEEELGSEAVYAGANFR.K<br>K.YGQDATNVGDEGGFAPNIQENK.E<br>K.YGQDATNVGDEGGFAPNIQENK.E<br>K.LAMQEFMILPVGASSFKEAMK.M + Oxidation (M)<br>K.KYGQDATNVGDEGGFAPNIQENK.E
A	30	gi|310689613	6-phosphogluconate dehydrogenase	Pinus pinaster	53200.01/6.74	10	K.LIDDVR.R<br>R.AIFLDR.I<br>K.VDETVER.A<br>K.GFPISVYNR.S<br>K.GFPISVYNR.S<br>R.DYFGAHTYER.I<br>R.DYFGAHTYER.I<br>R.ESLPANLVQAQR.D<br>R.ESLPANLVQAQR.D

							K.MVHNGIEYGDMQLIAEAYDVLK.S
C	31	gi|34597330	enolase	Brassica rapa subsp. campestris	47375.69/5.46	12	K.YNQLLR.I<br>R.AGWGVMASHR.S<br>R.AGWGVMASHR.S<br>R.AGWGVMASHR.S + Oxidation (M)<br>R.AGWGVMASHR.S + Oxidation (M)<br>K.VNQIGSVTESIEAVK.M<br>R.IEEELGSEAVYAGANFR.K<br>R.IEEELGSEAVYAGANFR.K<br>K.TYDLNFKEENNNGSQK.I<br>K.IVLPVPAFNVINGGSHAGNK.L<br>K.IVLPVPAFNVINGGSHAGNK.L<br>K.YGQDATNVGDEGGFAPNIQENK.E
C	38	gi|183397343	UDP-glucose pyrophosphorylase	Paulownia sp. ZKC-2008	51679/5.66	7	K.SVIEVR.N<br>K.VANFLSR.F<br>K.KVANFLSR.F<br>R.ANPANPSIDLGPEFK.K<br>R.ANPANPSIDLGPEFK.K<br>K.VQLLEIAQVPDEHVNEFK.S<br>K.VQLLEIAQVPDEHVNEFK.S
A	40	gi|298549053	Isocitrate dehydrogenase [NADP]	Cucumis sativus	46175.78/6.00	16	R.SPNGTIR.N<br>R.HAFGDQYR.A<br>R.HAFGDQYR.A<br>K.WPLYLSTK.N<br>R.NILNGTVFR.E<br>R.NILNGTVFR.E

							K.YFDLGLPHR.D<br>K.YFDLGLPHR.D<br>K.TIEAEAAHGTVTR.H<br>K.FEAAGIWYEHR.L<br>K.FEAAGIWYEHR.L<br>K.SKFEAAGIWYEHR.L<br>K.SKFEAAGIWYEHR.L<br>K.VANPIVEMDGDEMTR.V<br>K.GGETSTNSIASIFAWSR.G<br>K.GGETSTNSIASIFAWSR.G
A	41	gi|298549053	Isocitrate dehydrogenase [NADP]	Cucumis sativus	46175.78/6.00	16	R.SPNGTIR.N<br>R.HAFGDQYR.A<br>R.HAFGDQYR.A<br>K.WPLYLSTK.N<br>R.NILNGTVFR.E<br>R.NILNGTVFR.E<br>K.YFDLGLPHR.D<br>K.YFDLGLPHR.D<br>K.TIEAEAAHGTVTR.H<br>K.FEAAGIWYEHR.L<br>K.FEAAGIWYEHR.L<br>K.SKFEAAGIWYEHR.L<br>K.SKFEAAGIWYEHR.L<br>K.VANPIVEMDGDEMTR.V<br>K.GGETSTNSIASIFAWSR.G<br>K.GGETSTNSIASIFAWSR.G

C	42	gi|306755938	Malate dehydrogenase	Pseudotsuga menziesii	2519.92/4.43	4	K.LFGVTTLDVVR.-<br>K.LFGVTTLDVVR.-<br>DDLFNINAGIVK.L<br>DDLFNINAGIVK.L
C	45	gi|51701931	Ribonuclease-like storage protein	Panax ginseng	27340.10/5.87	12	K.QTDYFR.T<br>R.SDYPWAMFALR.L<br>R.SDYPWAMFALR.L<br>R.SDYPWAMFALR.L + Oxidation (M)<br>R.SDYPWAMFALR.L + Oxidation (M)<br>K.HLNAVPEIDFTK.N<br>K.SLLNTFTIHGLYPYNAK.G<br>K.SLLNTFTIHGLYPYNAK.G<br>K.AFDIVGLLNQEGIYPNNDLYRPK.M<br>K.AFDIVGLLNQEGIYPNNDLYRPK.M<br>R.KAFDIVGLLNQEGIYPNNDLYRPK.M<br>R.KAFDIVGLLNQEGIYPNNDLYRPK.M
A	46	gi|51701931	Ribonuclease-like storage protein	Panax ginseng	27340.10/5.87	5	K.QTDYFR.T<br>R.SDYPWAMFALR.L<br>R.SDYPWAMFALR.L<br>K.HLNAVPEIDFTK.N<br>K.HLNAVPEIDFTK.N
E	52	gi|51701931	Ribonuclease-like storage protein	Panax ginseng	27340.10/5.87	11	K.QTDYFR.T<br>R.SDYPWAMFALR.L<br>R.SDYPWAMFALR.L<br>R.SDYPWAMFALR.L + Oxidation (M)<br>R.SDYPWAMFALR.L + Oxidation (M)

							K.HLNAVPEIDFTK.N<br>K.SLLNTFTIHGLYPYNAK.G<br>K.SLLNTFTIHGLYPYNAK.G<br>K.AFDIVGLLNQEGIYPNNDLYRPK.M<br>K.AFDIVGLLNQEGIYPNNDLYRPK.M<br>R.KAFDIVGLLNQEGIYPNNDLYRPK.M
B	71	gi|3377841	phosphofructokinases	Arabidopsis thaliana	64171.6/5.57	20	TIDGDLK<br>TLVIGCPK<br>GSSLFGFR<br>YYHFVR<br>GGPAGIMKGK<br>TIDGDLKSK<br>IETPEQFK<br>EEWALKNR<br>MIGNVMIDAR<br>MIGNVMIDAR<br>YISPGPIQFK<br>MIGNVMIDAR<br>YYHFVRLMGR<br>IDHTLPLPSVFK<br>EVPTSFGFDTACK<br>MLIQMVETELEK<br>GPGSDARNHTLMLELGAQA<br>GPGSDARNHTLMLELGAQA<br>MDLDGLVVIGGDDSNTNACLLAEHFRAK

							MDLDGLVVIGGDDSNTNACLLAEHFRAK
D	28	gi|34597330	enolase	Brassica rapa subsp. campestris	47346/5.46	21	R.QIFDSR.G<br>K.YNQLLR.I<br>R.AGWGVMASHR.S<br>R.AGWGVMASHR.S<br>R.AGWGVMASHR.S + Oxidation (M)<br>R.AGWGVMASHR.S + Oxidation (M)<br>K.MGVEVYHNLK.S<br>K.MGVEVYHNLK.S + Oxidation (M)<br>K.VNQIGSVTESIEAVK.M<br>K.MGVEVYHNLKSVIK.K<br>K.MGVEVYHNLKSVIK.K<br>R.IEEELGSEAVYAGANFR.K<br>R.IEEELGSEAVYAGANFR.K<br>K.TYDLNFKEENNNGSQK.I<br>K.TYDLNFKEENNNGSQK.I<br>K.IVLPVPAFNVINGGSHAGNK.L<br>K.IVLPVPAFNVINGGSHAGNK.L<br>R.SGETEDTFIADLSVGLSTGQIK.T<br>K.YGQDATNVGDEGGFAPNIQENK.E<br>K.YGQDATNVGDEGGFAPNIQENK.E<br>K.KYGQDATNVGDEGGFAPNIQENK.E
E	29	gi|183397343	UDP-glucose pyrophosphorylase	Paulownia sp. ZKC-2008	51679/5.66	6	K.SVIEVR.N<br>K.VANFLSR.F<br>R.ANPANPSIDLGPEFK.K

							R.ANPANPSIDLGPEFK.K<br>K.VQLLEIAQVPDEHVNEFK.S<br>K.VQLLEIAQVPDEHVNEFK.S
F	37	gi|17402533	UDP-glucose pyrophosphorylase	Nicotiana tabacum	41001/7.12	6	K.SVIEVR.N<br>K.ILNHLIQNK.N<br>K.SIPSIIDLDSLK.V<br>R.SNPSNPAIELGPEFK.K<br>K.VQLLEIAQVPDEHVNEFK.S<br>K.VQLLEIAQVPDEHVNEFK.S
F	51	gi|51701931	Ribonuclease-like storage protein	Panax ginseng	27340.10/5.87	5	K.QTDYFR.T<br>R.SDYPWAMFALR.L<br>R.SDYPWAMFALR.L<br>K.HLNAVPEIDFTK.N<br>K.HLNAVPEIDFTK.N
E	57	gi|226493589	ATP synthase beta chain	Zea mays	58942.8/5.9	20	VVDLLAPYQR<br>VVDLLAPYQR<br>AHGGFSVFAGVGER<br>AHGGFSVFAGVGER<br>VGLTGLTVAEHFR<br>VGLTGLTVAEHFR<br>VLNTGSPITVPVGR<br>VLNTGSPITVPVGR<br>TVLIMELINNVAK<br>FTQANSEVSALLGR<br>FTQANSEVSALLGR<br>GDITTNHFLPIHR

							GAPHRPSPAGYLFNR<br>LVLEVAQHLGENMVR<br>ATLGRIINVIGEPIDEK<br>DAEGQDVLLFIDNIFR<br>EGNDLYREMIESGVIK<br>QISELGIYPAVDPLDSTSR<br>IPSAVGYQPTLATDLGGLQER<br>IPSAVGYQPTLATDLGGLQER
B	58	gi|217940	beta-amylase	Ipomoea batatas	56014.8/5.18	4	R.DGYRPIAR.M<br>R.DGYRPIAR.M<br>K.QYDWSAYR.E<br>K.QYDWSAYR.E
D	65	gi|6706331	cofactor-independent phosphoglyceromutase	Apium graveolens	60896.7/5.26	9	TLRAEPK<br>MYVTMDR<br>TSGEYLTHNGVR<br>TSGEYLTHNGVR<br>EQGIDAQIASGGGR<br>FGHVTFFWNGNR<br>ESFESGTLHLIGLLSDGGVHSR<br>ESFESGTLHLIGLLSDGGVHSR<br>AHGTAVGLPTEDDMGNSEVGHNALGAGR
ROS scavenging and defence							
B	6	gi|316937082	SGF14n	Glycine max	30474/4.85	14	R.YLAEFK.A<br>K.LAEQAER.Y

							K.MKGDYYR.Y<br>K.MKGDYYR.Y + Oxidation (M)<br>K.DSTLIMQLLR.D<br>K.DSTLIMQLLR.D<br>K.DSTLIMQLLR.D + Oxidation (M)<br>K.DSTLIMQLLR.D + Oxidation (M)<br>M.TQPAMATFSKER.E<br>K.LDVELSVEERNLFSVGYK.N<br>K.LDVELSVEERNLFSVGYK.N<br>R.LGLALNFSVFYYEIMNSPER.A<br>R.LGLALNFSVFYYEIMNSPER.A + Oxidation (M)<br>R.LGLALNFSVFYYEIMNSPER.A + Oxidation (M)
B	66	gi|87241037	Heat shock protein Hsp70	Medicago truncatula	71046.1/5.11	26	CLRDAK<br>DISGNPR<br>FELSGIPPAPR<br>VEIIANDQGNR<br>EIAEAYLGSAIK<br>MVNHFVQEFK<br>FEELNMDLFR<br>FEELNMDLFR<br>FEELNMDLFR<br>NALENYAYNMR<br>RFSDASVQSDMK<br>VQQLLQDFFNGK<br>MVNHFVQEFKR<br>TTPSYVAFTDSER

							MREIAEAYLGSAIK<br>ARFEELNMDLFR<br>ARFEELNMDLFR<br>MREIAEAYLGSAIK<br>SVHDVVLVGGSTRIPK<br>ATAGDTHLGGEDFDNR<br>NAVVTVPAYFNDSQR<br>NAVVTVPAYFNDSQR<br>IINEPTAAAIAYGLDKK<br>EQVFSTYSDNQPGVLIQVFEGER<br>EQVFSTYSDNQPGVLIQVFEGER<br>KEQVFSTYSDNQPGVLIQVFEGER
F	12	gi|197725474	major latex-like protein	Panax ginseng	16860/4.86	2	K.DPTSYLDFLLSVTR.D<br>K.DPTSYLDFLLSVTR.D
D	18	gi|296125055	Peroxiredo0xin	Panax ginseng	17397/5.37	13	K.FLADGSAK.Y<br>K.HVPGFIEK.A<br>K.HVPGFIEK.A<br>R.FALLVDDLK.V<br>R.FALLVDDLK.V<br>R.RFALLVDDLK.V<br>R.RFALLVDDLK.V<br>K.YTHALGLELDLSEK.G<br>K.YTHALGLELDLSEK.G<br>K.GVDEILLISVNDPFVMK.A<br>K.GVDEILLISVNDPFVMK.A + Oxidation (M)<br>K.GVDEILLISVNDPFVMK.A + Oxidation (M)

							M.APIAVGDSLPDGTLAFFDAEDQLQQVSVHSLAAGK.K
D	33	gi|116077986	pterocarpan reductase	Lotus japonicus	33990.97/ 5.94	5	K.IGKTVEK.V<br>K.AGHPTFALVR.E<br>K.AGHPTFALVR.E<br>R.FLPSEFGNDVDR.T<br>R.FLPSEFGNDVDR.T
D	67	gi|148910696	Heat shock protein Hsp70	Picea sitchensis	71266.3/5.07	27	CLRDAK<br>FELSGIPPAPR<br>VEIIANDQGNR<br>DAGVISGLNVMR<br>FSDPTVQNDMK<br>FEELNMDLFR<br>FEELNMDLFR<br>FEELNMDLFR<br>NSLENYAYNMR<br>NSLENYAYNMR<br>VQQLLQDFFNGK<br>STIHDVVLVGGSTR<br>DEIEKMVQDAEK<br>TTPSYVAFTDTER<br>TTPSYVAFTDTER<br>ARFEELNMDLFR<br>ARFEELNMDLFR<br>MKEIAEAYLGTTIK<br>NQVAMNPTNTVFDAK<br>ATAGDTHLGGEDFDNR

							NAVVTVPAYFNDSQR<br>NAVVTVPAYFNDSQR<br>IINEPTAAAIAYGLDKK<br>EQVFSTYSDNQPGVLIQVFEGER<br>EQVFSTYSDNQPGVLIQVFEGER<br>KEQVFSTYSDNQPGVLIQVFEGER<br>TLSSTAQTTIEIDSLYEGIDFYSTITR
Transcription and translation							
C	21	gi|62642102	eukaryotic translation initiation factor 5A	Picea abies	16243/5.27	4	K.TFPQQAGTIR.K<br>K.TFPQQAGTIR.K<br>M.SDEDNHFESK.A<br>K.DGFGEGKDLVVTVMSAMGEEQ.-
C	36	gi|20138786	Eukaryotic translation initiation factor 5A;	Senecio vernalis (Spring groundsel)	17255/5.60	4	K.LPTDDALLTQIK.D<br>K.DDLKLPTDDALLTQIK.D<br>K.DDLKLPTDDALLTQIK.D<br>K.KLEDIVPSSHNCDVPHVNR.T
Regulation of cell cycle							
F	7	gi|129692	Cyclin	Daucus carota (Carrot)	29176/4.67	14	R.YMNSFTK.A<br>R.MPSAEFAR.I<br>R.MPSAEFAR.I<br>R.MPSAEFAR.I + Oxidation (M)<br>R.MPSAEFAR.I + Oxidation (M)

							K.IAEMGYIR.F<br>K.IAEMGYIR.F<br>K.IAEMGYIR.F + Oxidation (M)<br>K.IAEMGYIR.F + Oxidation (M)<br>R.SEGFEHYR.C<br>R.SEGFEHYR.C<br>K.LMDIDSEHLGIPEAEYHAIVR.M<br>K.LMDIDSEHLGIPEAEYHAIVR.M<br>K.LMDIDSEHLGIPEAEYHAIVR.M + Oxidation (M)
E	56	gi|149938964	act1	Actinidia deliciosa	41637/5.31	27	VVAPPER<br>RGILTLK<br>VVAPPERK<br>AGFAGDDAPR<br>AGFAGDDAPR<br>IKVVAPPER<br>GYMFTTTAER<br>GYMFTTTAER<br>AVFPSIVGRPR<br>AVFPSIVGRPR<br>DAYVGDEAQSKR<br>GEYDESGPSIVHR<br>GEYDESGPSIVHR<br>IWHHTFYNELR<br>LDLAGRDLTDALMK<br>LDLAGRDLTDALMK<br>LDLAGRDLTDALMK

							GEYDESGPSIVHRK<br>GYMFTTTAEREIVR<br>NYELPDGQVITIGAER<br>NYELPDGQVITIGAER<br>VAPEEHPVLLTEAPLNPK<br>ADAEDIQPLVCDNGTGMVK<br>ADAEDIQPLVCDNGTGMVK<br>DLYGNIVLSGGSTMFPGIADR<br>SSSSVEKNYELPDGQVITGAER<br>TTGIVLDSGDGVSHTVPIYEGYALPHAILR
Protein synthesis and decomposition							
C	60	gi|1303695	BIP	Arabidopsis thaliana	73446.9/5.08	25	EEYDEK<br>IMEYFIK<br>ALSSQHQVR<br>DAGVIAGLNVAR<br>SLTKDCSLLGK<br>FDLTGVPPAPR<br>FDLTGVPPAPR<br>FEELNNDLFR<br>SQIDEIVLVGGSTR<br>SQIDEIVLVGGSTR<br>ITPSWVGFTDSER<br>ARFEELNNDLFR<br>ARFEELNNDLFR

							NGHVEIIANDQGNR<br>VFSPEEISAMILTK<br>DAVVTVPAYFNDAQR<br>LIGEAAKNQAAVNPER<br>IINEPTAAAIAYGLDKK<br>IKDAVVTVPAYFNDAQR<br>IKDAVVTVPAYFNDAQR<br>LGSVIGIDLGTTYSCVGVYK<br>LVPYQIVNKDGKPYIQVK<br>SQVFTTYQDQQTTVSIQVFEGER<br>SQVFTTYQDQQTTVSIQVFEGER<br>KSQVFTTYQDQQTTVSIQVFEGER
C	61	gi|15241844	BIP1	Arabidopsis thaliana	73583.9/5.08	17	NQAAVNPER<br>ALSSQHQVR<br>DAGVIAGLNVAR<br>FEELNNDLFR<br>FEELNNDLFR<br>SQIDEIVLVGGSTR<br>SQIDEIVLVGGSTR<br>ARFEELNNDLFR<br>NGHVEIIANDQGNR<br>DAVVTVPAYFNDAQR<br>LIGEAAKNQAAVNPER<br>IINEPTAAAIAYGLDKK<br>IKDAVVTVPAYFNDAQR<br>IKDAVVTVPAYFNDAQR

							LGSVIGIDLGTTYSCVGVYK<br>VEIESLFDGVDFSEPLTR<br>SQVFTTYQDQQTTVSIQVFEGER
A	43	gi|297744621	Proteasome subunit alpha type	Vitis vinifera	27208.94/6.93	14	R.NSNSMR.E<br>R.NSNSMR.E + Oxidation (M)<br>K.GNAAVGVR.G<br>R.YIAGLQQK.Y<br>R.YIAGLQQK.Y<br>R.ALLEVVESGGK.N<br>R.GTDTIVLGVEK.K<br>R.GTDTIVLGVEK.K<br>R.LTVEDPVTVEYITR.Y<br>R.LTVEDPVTVEYITR.Y<br>R.AITVFSPDGHLFQVEYALEAVR.K<br>R.AITVFSPDGHLFQVEYALEAVR.K<br>R.AITVFSPDGHLFQVEYALEAVRK.G<br>R.AITVFSPDGHLFQVEYALEAVRK.G
A	44	gi|222854239	Proteasome subunit alpha type	Populus trichocarpa	27306.92/5.73	14	R.GSGGGYDR.H<br>R.NEAAEFR.F<br>R.LFQVEYAFK.A<br>R.LFQVEYAFK.A<br>R.HITIFSPEGR.L<br>R.HITIFSPEGR.L<br>K.EQEAINFLEK.K<br>R.YGYEMPVDALAR.W<br>R.YGYEMPVDALAR.W

							R.YGYEMPVDALAR.W + Oxidation (M)<br>R.YGYEMPVDALAR.W + Oxidation (M)<br>R.VLSTEEIDEHLTAISER.D<br>R.VLSTEEIDEHLTAISERD.-<br>R.VLSTEEIDEHLTAISERD.-
F	59	gi|1303695	BIP	Arabidopsis thaliana	73446.9/5.08	19	NQAAVNPER<br>ALSSQHQVR<br>DAGVIAGLNVAR<br>SLTKDCSLLGK<br>FDLTGVPPAPR<br>FDLTGVPPAPR<br>FEELNNDLFR<br>FEELNNDLFR<br>SQIDEIVLVGGSTR<br>SQIDEIVLVGGSTR<br>ARFEELNNDLFR<br>NGHVEIIANDQGNR<br>DAVVTVPAYFNDAQR<br>LIGEAAKNQAAVNPER<br>IINEPTAAAIAYGLDKK<br>IKDAVVTVPAYFNDAQR<br>IKDAVVTVPAYFNDAQR<br>LGSVIGIDLGTTYSCVGVYK<br>SQVFTTYQDQQTTVSIQVFEGER

Signal transduction							
C	47	gi|73808794	cytosolic nucleoside diphosphate kinase	Solanum chacoense	16221.70/6.30	6	R.GLVGEIIGR.F<br>R.GLVGEIIGR.F<br>R.GDFAIDIGR.N<br>R.GDFAIDIGR.N<br>R.NVIHGSDAVESAR.K<br>R.NVIHGSDAVESAR.K
Other							
A	70	gi|297601991	os03g0850300	Oryza sativa Indica Group	26712.3/6.2	9	RRPSVR<br>LYAAIQK<br>KLYAAIQK<br>KLYAAIQK<br>DMGIGAVGSR<br>DMGIGAVGSRR<br>TSSSSHPRPPR<br>HHKTSSSSHPRPPR<br>LGDIGVHSAAAADVPLPQRR
