# Supplementary material for: Proteomic Analyses Provide Novel Insights into Plant Growth and Ginsenoside Biosynthesis in Forest Cultivated Panax ginseng (F. Ginseng)
Source: Front Plant Sci. 2016 Jan 26;7:1. doi: 10.3389/fpls.2016.00001 (PMC4726751; doi:10.3389/fpls.2016.00001)
Supplement: Table S3 — Identification of different expressed proteins from F. Ginseng in different growth years by iTRAQ. Expression differences were defined as protein abundance ratios greater than 1.4 or less than 0.6. aClusters, The clusters of abundance of differentially expressed proteins in Figure 5; bAccession number in NCBI database.c15/10, the ratios of protein abundances between 15 and 10 years of F. Ginseng; d20/10, the ratios of protein abundances between 20 and 10 years of F. Ginseng; e25/10, the ratios of protein abundances between 25 and 10 years of F. Ginseng. [file Table3.PDF]

Table S3.

Clusters [a]	Protein name [Species]	Accession no.[b]	Mr(kDa)	Credibility	15/10[c]	20/10[d]	25/10[e]
A	Protein ycf2 [Panax ginseng]	Q68RU4	276710.59	100.00	1.45	1.47	1.21
A	O-linked n-acetylglucosamine transferase, ogt, putative [Ricinus communis]	B9R8W2	153398.53	99.84	1.40	1.46	1.43
A	SWI/SNF class chromatin remodeling complex protein [Physcomitrella patens subsp. Patens]	A9RSB9	298320.72	95.50	1.36	1.71	1.61
A	60S ribosomal protein L27 [Panax ginseng]	Q9MAV8	19570.49	95.42	1.25	1.27	1.62
A	NAD(P)H-quinone oxidoreductase subunit H, chloroplastic[Sesamum indicum]	G9IBB9	50429.86	95.14	1.44	1.81	1.16
A	Maturase [Tetraplasandra hawaiensis]	AAB66296.1	45063.21	94.03	2.63	2.96	3.30
A	Transcription initiation factor, putative [Ricinus communis]	B9S3E1	112669.92	92.73	1.31	1.48	1.47
A	Calcium-dependent protein kinase 1 (Fragment) [Panax ginseng]	A5HNW6	14846.94	89.07	2.85	4.68	1.08
A	Alpha-copaene synthase [Eleutherococcus trifoliatus]	H9L9E5	73268.68	88.66	1.11	1.75	1.06
A	Photosystem II CP47 chlorophyll apoprotein [Sesamum indicum]	G9IB88	60548.55	80.52	1.74	1.99	1.03
A	Os11g0121600 protein [Oryza sativa subsp. Japonica]	Q0IV06	19078.88	79.98	1.48	1.70	1.42
A	NB-LRR type disease resistance protein Rps1-k-2 [Glycine max]	Q2YE87	158923.34	72.87	1.40	1.45	1.36
A	Histone-lysine N-methyltransferase E(Z) [Medicago truncatula]	G7IRJ2	311991.13	72.58	1.27	1.44	1.13
A	Adenylate isopentenyltransferase [Humulus lupulus]	Q5GHF7	40329.34	71.86	1.27	1.39	1.31
A	Synaptotagmin-1 [Medicago truncatula]	G7I517	67537.30	70.41	4.29	4.73	2.59
A	Calmodulin binding protein, putative [Ricinuscommunis]	B9RER0	55011.54	68.57	2.04	2.31	2.28
A	NBS-LRR protein (Fragment) [Oryza sativa subsp. Japonica]	H9DWU8	36903.28	67.11	1.16	1.54	2.47
A	Dehydration responsive element binding protein [Populus euphratica]	A1XCN0	34461.87	62.41	1.74	2.09	2.46
B	PR10 [Panax ginseng]	D0VNY8	18918.21	100.00	1.31	1.16	1.33
B	PR10-1 (Fragment) [Panax ginseng]	E9M219	18994.45	100.00	1.83	1.19	1.23
B	DNA ligase (Fragment) [Chlorella variabilis]	E1ZSH7	81693.81	99.30	1.31	0.98	1.22
B	Phosphatidylinositol 3-kinase	Q8GUA6	99642.60	99.28	1.47	0.95	1.13

	[Medicago truncatula]						
B	Ribonuclease 1 [Panax ginseng]	P80889	18142.90	99.12	1.45	1.27	1.42
B	Catalytic, putative [Ricinus communis]	B9T126	55999.79	98.34	1.30	1.17	2.56
B	Ycf1 gene product (chloroplast) [Sesamum indicum]	YP_004935725.1	242064.80	98.21	1.66	1.10	1.90
B	Putative elongation factor 1-alpha (Fragment) [Aralia elata]	I7DFU2	37582.53	97.94	1.25	0.96	1.94
B	At5g37010 [Arabidopsis thaliana]	Q9FGV2	75638.99	97.78	1.80	1.51	1.58
B	Os02g0157150 protein [Oryza sativa subsp. Japonica]	A3A3B1	72220.59	97.32	1.70	1.22	1.82
B	15-hydroxyprostaglandin dehydrogenase [Medicago truncatula]	G7JLA7	75485.82	95.97	1.78	1.72	1.05
B	Probably inactive leucine-rich repeat receptor-like protein kinase [Medicago truncatula]	G7JY72	63452.32	95.09	1.61	1.20	1.17
B	30S ribosomal protein S15, chloroplastic [Sesamum indicum]	G9IBC0	12678.28	91.07	1.70	1.15	1.05
B	Dehydrin 8 [Panax ginseng]	Q1HGF0	33119.49	90.39	1.76	1.43	1.03
B	Stress-induced receptor-like kinase [Glycine max]	B2ZHZ5	89050.75	90.04	2.14	1.16	1.11
B	Cytosine-specific methyltransferase [Oryza brachyantha]	J3M520	136306.06	89.50	1.75	1.48	1.26
B	Anthocyanin 3-O-galactosyltransferase [Aralia cordata]	Q76G23	53742.67	87.45	1.73	1.44	1.00
B	Chloroplast zebra-necrosis protein [Oryza sativa subsp. Japonica]	Q8H658	38970.20	87.03	1.96	1.38	1.42
B	Reticuline oxidase-like protein [Medicago truncatula]	G7IMM5	65762.25	86.81	2.19	0.66	1.95
B	APETALA3-like MADS box protein [Crocus sativus]	Q4KPI7	28763.46	86.59	1.64	1.47	1.46
B	NADH dehydrogenase subunit 5 [Eleutherococcus senticosus]	G9IB23	93490.73	86.24	1.01	0.59	1.10
B	WGS project CAID00000000 data, contig chromosome 17 (Fragment) [Ostreococcus tauri]	Q00TR2	51996.70	85.96	2.57	2.50	3.05
B	Os02g0619600 protein [Oryza sativa subsp. Japonica]	Q6K937	81027.63	85.41	1.98	1.76	2.98
B	Betaine aldehyde dehydrogenase [Panax ginseng]	Q6JSK3	61104.57	84.56	1.90	1.13	1.01
B	Cytokinin receptor histidine protein kinase [Petunia hybrida]	H1ABZ2	122717.29	82.69	1.54	1.44	1.47

B	CC-NBS-LRR [Helianthus annuus]	Q69AB7	157349.13	82.13	1.11	0.73	1.51
B	UDP-glucose:sterol 3-O-glucosyltransferase [Panax ginseng]	Q8H9B5	75393.59	81.69	2.39	1.50	1.03
B	WRKY9 [Panax quinquefolius]	K4EN49	43908.48	78.83	1.23	0.54	1.17
B	Class 1 chitinase [Panax ginseng]	C1K2M3	38787.41	78.44	1.39	1.00	1.07
B	Cytochrome P450 mono-oxygenase superfamily (Fragment) [Picea glauca]	E5FQZ0	26054.61	77.97	1.96	0.83	1.26
B	Minus agglutinin [Chlamydomonas incerta]	Q5I2R0	417926.19	75.15	1.51	1.39	1.31
B	DNA repair protein Rad50 [Medicago truncatula]	G7JAR6	198608.61	75.11	1.52	1.16	1.97
B	Phosphoglycerate kinase [Prunus persica]	M5WN82	56406.47	71.13	1.35	1.27	1.26
B	F-box domain containing protein [Oryza sativa subsp. Japonica]	Q75GY3	55432.30	69.93	4.29	2.78	1.60
B	At5g57350/MJB24_16 [Arabidopsis thaliana]	Q93ZM8	112785.66	67.71	2.66	1.20	2.87
B	Disease resistance protein RGA2 [Medicago truncatula]	G7J0J3	23157.05	66.19	1.54	0.62	1.55
B	Calcineurin B-like protein 03 [Sorghum bicolor]	C4P7Y6	28421.06	64.35	2.78	0.95	3.44
C	Wall-associated receptor kinase-like protein [Medicago truncatula]	G7L302	65611.92	97.31	0.27	0.88	1.08
C	ATP synthase subunit alpha, chloroplastic [Panax ginseng]	Q68S21	60978.98	97.08	0.50	0.70	1.10
C	NAD(P)H-quinone oxidoreductase subunit 1, chloroplastic [Eleutherococcus senticosus]	G9IB31	43760.33	97.02	0.42	0.58	1.03
C	Short-chain alcohol dehydrogenase [Panax ginseng]	B8YDG5	33172.03	89.29	0.39	1.27	1.08
C	Maturase, partial (chloroplast) [Cussonia spicata]	AAB64057.1	44993.83	88.52	0.94	1.71	1.30
C	ACT domain containing protein, expressed [Oryza sativa subsp. Japonica]	Q10P43	49594.57	87.09	0.12	1.15	2.29
C	Probable cinnamyl alcohol dehydrogenase 1 [Aralia cordata]	P42495	44867.64	80.74	0.30	0.49	1.06
C	50S ribosomal protein L2, chloroplastic [Eleutherococcus senticosus]	G9IB17	34760.55	77.20	0.51	0.78	1.04
C	WRKY35 [Panax quinquefolius]	K4ENK1	42656.51	77.02	0.40	0.71	1.07
C	Os02g0670700 protein [Oryza sativa subsp. Japonica]	Q6ESP7	82355.78	64.67	0.26	0.65	1.63

C	Non-SMC (Structural maintenance of chromosomes) element1 protein (NSE1) (ISS) [Ostreococcus tauri]	Q015A7	31478.47	62.98	0.99	1.36	1.19
D	PR10-3 (Fragment) [Panax ginseng]	E9M220	19149.40	89.89	0.78	0.61	1.01
D	Chlorophyll a/b binding protein [Panax ginseng]	Q3LFQ5	31793.77	86.80	0.77	0.50	1.01
D	50S ribosomal protein L14, chloroplastic [Panax ginseng]	Q68RX0	14625.08	85.75	0.87	0.38	1.00
D	30S ribosomal protein S18, chloroplastic [Sesamum indicum]	G9IB84	13593.88	85.62	0.70	0.33	1.10
D	UDP-glucosyltransferase [Panax notoginseng]	I7BX75	61325.67	81.98	0.40	0.32	1.02
D	Ribulose bisphosphate carboxylase large chain (Fragment)[Osmoxylon novoguineense]	Q6Y2Y1	54391.20	80.87	0.68	0.33	1.11
D	Beta-amyrin synthase AS2 (Fragment) [Eleutherococcus senticosus]	G1CI86	54221.22	77.73	0.61	0.42	1.08
E	Squalene epoxidase [Panax ginseng]	Q75W20	65550.26	100.00	0.58	0.41	0.35
E	Ycf1 [Sesamum indicum]	G9IBC1	251287.33	100.00	0.66	0.42	0.72
E	Ycf1 protein [Eleutherococcus senticosus]	G9IB34	265961.03	100.00	0.81	0.70	0.60
E	Cytochrome P450 CYP716A53v2 [Panax ginseng]	I7CT85	60323.64	99.53	0.97	0.94	0.51
E	Glutamate receptor [Populus trichocarpa]	B9H5K3	112396.84	99.17	0.48	0.41	0.52
E	Galactose-1-phosphate uridylyltransferase [Ricinus communis]	B9SHG4	42218.02	94.65	0.45	0.45	0.44
E	Cytohesin 1, 2, 3, putative [Ricinus communis]	B9RDE3	212744.13	94.26	0.49	0.47	0.34
E	WRKY3 [Panax quinquefolius]	K4EN16	57836.56	93.09	0.85	0.57	0.88
E	Chromatin assembly factor 1, subunit A, putative [Ricinus communis]	B9RM13	106386.59	86.29	0.26	0.20	0.43
E	Cytoplasmic ribosomal protein S13 [Panax ginseng]	Q9MAV9	20854.22	80.74	0.66	0.28	0.97
E	MLP-like protein 31 [Arabidopsis thaliana]	Q941R6	21719.53	80.00	0.89	0.15	0.34
E	DEAD-box ATP-dependent RNA helicase [Medicago truncatula]	G7I8N0	77473.04	78.60	0.51	0.50	0.55
E	Self-incompatibility ribonuclease (Fragment) [Lycium hirsutum]	F1B0B4	17377.33	75.54	0.77	0.75	0.60
E	DEAD-box ATP-dependent RNA helicase 53 [Arabidopsis thaliana]	Q9LUW5	69209.84	70.93	0.57	0.48	0.56

E	RHO protein GDP dissociation inhibitor [Medicago truncatula]	G7JKI7	28124.51	65.64	0.64	0.54	0.25
E	P-loop containing nucleoside triphosphate hydrolase protein [Coccomyxa subellipsoidea]	I0Z4L4	145332.92	62.90	0.55	0.48	0.60
F	Major latex-like protein [Panax ginseng]	B5THI3	19021.97	100.00	0.95	1.01	0.42
F	Mevalonate diphosphate decarboxylase [Panax ginseng]	D0EAP4	50992.14	100.00	0.48	0.76	0.77
F	Squalene synthase [Panax quinquefolius]	N0BP33	67193.17	100.00	0.50	0.51	0.35
F	Polygalacturonase inhibiting protein [Panax ginseng]	C7FE10	42875.72	99.71	0.91	0.94	0.50
F	WRKY7 [Panax quinquefolius]	K4ENK0	88122.66	99.64	0.27	0.54	0.23
F	Cycloartenol Synthase [Panax ginseng]	O82139	95997.00	99.58	0.56	0.72	0.46
F	Maturase (Fragment) [Hedera helix]	A1XJ19	73987.84	99.36	0.43	0.64	0.58
F	Voltage-gated Ca2+ channel, alpha subunit [Chlamydomonas reinhardtii]	A8IVU6	488313.22	98.87	0.55	0.72	0.62
F	Oxidosqualene Cyclase [Panax ginseng]	O82141	99148.56	98.59	0.34	0.36	0.44
F	PR10-2 [Panax ginseng]	ADW93869.1	18341.80	98.29	0.38	0.69	0.44
F	Ribulose bisphosphate carboxylase small chain [Aralia elata]	I7DCG5	23114.15	97.87	0.51	0.82	0.71
F	DNA-directed RNA polymerase[Sesamum indicum]	G9IB53	89618.41	96.77	0.59	0.74	0.75
F	Cysteine protease ATG4b [rabidopsis thaliana]	F4J9I3	41929.30	93.45	0.82	2.79	0.61
F	ATP synthase subunit b, chloroplastic [Eleutherococcus senticosus]	G9IAW1	22860.21	93.13	0.92	1.10	0.61
F	Expressed protein [Chlorella variabilis]	E1ZI29	65821.44	89.30	0.47	0.49	0.53
F	U-box domain-containing protein [Medicago truncatula]	G7KEX0	91073.49	88.14	0.55	0.59	0.40
F	WD40 repeat-like protein [Coccomyxa subellipsoidea]	I0Z3A3	82598.54	86.96	0.61	0.73	0.68
F	Formin-like protein [Medicago truncatula]	G7K5M3	103362.42	85.17	0.12	0.36	0.21
F	EXECUTER1 protein, chloroplast, putative [Ricinus communis]	B9T3E6	84923.73	83.31	0.52	0.62	0.55
F	Protein translocase subunit SecA [Physcomitrella patens subsp. Patens]	A9U203	121276.45	79.63	0.15	0.59	0.40
F	AT4G23700-like protein (Fragment)	D6PR23	19818.47	78.60	0.47	0.49	0.53

	[Neslia paniculata]							
F	Retrotransposon protein, putative, Ty3-gypsy subclass [Oryza sativa subsp. Japonica]	Q2R064	24912.60	74.17	0.59	0.63	0.58
F	En/Spm-like transposon protein, putative [Arabidopsis thaliana]	Q9C6Z6	48961.16	72.89	0.21	1.19	0.97
F	Disease resistance protein RPM1 [Aegilops tauschii]	M8BV24	58802.65	71.53	0.59	0.59	0.63
F	Epoxide hydrolase [Medicago truncatula]	G7J939	38144.73	68.64	0.53	0.56	0.65
F	Os04g0244500 protein [Oryza sativa subsp. Japonica]	Q0JEM0	43736.29	64.67	0.53	0.59	0.46
G	Cytochrome P450 [Panax notoginseng]	F4YF76	60105.31	100.00	1.09	0.93	0.53
G	Putative membrane protein ycf1 [Panax ginseng]	Q68RU8	269926.81	100.00	1.14	0.85	0.60
G	HTH-type transcriptional regulator ptxR, putative (Fragment) [Ricinus communis]	B9TER2	31016.66	98.95	1.97	1.00	0.98
G	Mitochondrial NADH ubiquinone oxidoreductase 13kD-like subunit [Zea mays]	B6SJ79	12831.88	98.94	1.18	0.32	0.71
G	Maturase K [Panax pseudoginseng]	Q7YIX8	68305.49	98.64	1.00	0.52	0.61
G	Ribosomal protein S4 [Panax ginseng]	Q401B8	35161.97	98.33	1.45	0.47	0.76
G	Cation/H(+) antiporter 27 [Arabidopsis thaliana]	Q9M007	91977.66	98.11	1.09	0.96	0.54
G	E2F transcription factor;1 [Physcomitrella patens subsp. Patens]	G1UDF7	47921.42	96.56	1.61	0.73	0.68
G	DS synthase [Panax notoginseng]	F4YF85	100008.32	94.57	1.00	0.62	0.90
G	Plastid delta4 multifunctional acyl-acyl carrier protein desaturase (Fragment) [Hedera helix]	Q4KN79	47711.31	94.17	1.38	1.32	0.76
G	Glycosyltransferase [Panax notoginseng]	F4YF68	86090.02	93.96	1.01	0.59	0.69
G	Cytochrome P450 (Fragment) [Panax notoginseng]	F4YF83	64672.10	93.68	1.53	0.80	0.47
G	NADH dehydrogenase subunit F (Fragment) [Tetraplasandra oahuensis]	E0XD62	85644.33	93.38	1.60	0.57	0.89
G	E3 ubiquitin-protein ligase UPL2 [Arabidopsis thaliana]	Q8H0T4	425268.22	92.57	2.58	0.84	0.75
G	DNA-directed RNA polymerase subunit alpha [Eleutherococcus senticosus]	G9IB07	45446.79	89.80	1.42	0.74	0.95
G	Ribosomal protein S4 (Fragment)	O63017	25763.61	88.96	1.36	0.56	0.68

	[Pohlia cruda]						
G	Aralin B chain (Fragment) [Aralia elata]	P83573	3264.82	88.37	1.43	1.13	0.98
G	Os05g0584600 protein [Oryza sativa subsp. Japonica]	Q6I591	104024.96	85.00	1.66	0.98	0.50
G	ribulose-1,5-bisphosphate carboxylase/oxygenase large subunit, partial (chloroplast) [Billardiera heterophylla]	AAA98956.1	56102.34	84.51	2.22	0.27	0.95
G	Putative transport protein [Arabidopsis thaliana]	F4HZB2	429171.81	81.16	1.69	1.13	0.45
G	Hypothetical retrotransposon [Ipomoea batatas]	Q5MG99	179705.61	78.47	2.18	0.55	0.14
G	NBS4-OO [Oryza officinalis]	E2CU78	127358.59	78.27	1.07	0.21	0.64
G	Centromere protein, putative [Medicago truncatula]	Q2HVA4	118903.67	77.86	2.28	0.35	0.55
G	30S ribosomal protein S4, chloroplastic [Huperzia lucidula]	Q5SD08	26283.96	72.58	1.52	0.73	0.65
G	Receptor-like protein kinase [Medicago truncatula]	G7IN89	118349.54	70.75	2.70	0.34	0.82
H	DNA-directed RNA polymerase subunit beta" [Panax ginseng]	Q68S16	175106.59	100.00	1.06	1.40	0.62
H	DNA-directed RNA polymerase (Fragment) [Hydrocotyle vulgaris]	E2DGT3	74854.61	99.25	1.48	1.74	0.85
H	Photosystem II CP47 chlorophyll apoprotein (Fragment) [Hedera helix]	E0XDM6	59029.88	97.36	1.12	1.96	0.64
H	ATP binding protein, putative [Ricinus communis]	B9SLE3	72184.90	93.22	1.18	2.67	0.89
H	Farnesyl diphosphate synthase [Eleutherococcus senticosus]	H2EV94	46942.51	78.54	1.52	2.05	0.90
	OO_Ba0013J05-OO_Ba0033A15.25 protein [Oryza officinalis]	D0ABG8	195666.17	64.97	0.79	0.86	0.82
	Os01g0643300 protein [Oryza sativa subsp. Japonica]	Q0JKX2	66168.96	27.83	0.83	0.75	0.72
	Os02g0468400 protein [Oryza sativa subsp. Japonica]	Q6K8I5	52662.17	30.12	0.77	0.65	0.72
	Os06g0680900 protein [Oryza sativa subsp. Japonica]	Q0DA35	54345.60	14.42	2.25	2.18	1.92
	Os07g0649700 protein [Oryza sativa subsp. Japonica]	C7J5B4	179255.23	0.88	0.60	0.66	0.66
	Os10g0339101 protein [Oryza sativa subsp. Japonica]	A3C3Q1	10161.31	39.69	0.94	1.02	1.11
	Os11g0121600 protein [Oryza sativa subsp. Japonica]	Q0IV06	19078.88	79.98	1.48	1.70	1.42

	Os11g0243400 protein [Oryza sativa subsp. Japonica]	Q0ITH7	11249.17	77.18	0.85	0.91	0.77
	Predicted protein (Fragment) [Chlamydomonas reinhardtii]	A8HQN5	169420.25	29.95	0.00	0.00	0.00
	Predicted protein (Fragment) [Chlamydomonas reinhardtii]	A8HM58	174474.39	75.37	0.57	0.58	0.59
	Predicted protein (Fragment) [Chlamydomonas reinhardtii]	A8IUT1	105144.49	69.72	0.54	0.47	0.50
	Predicted protein (Fragment) [Chlamydomonas reinhardtii]	A8JB42	36077.85	10.80	0.89	0.96	0.92
	Predicted protein (Fragment) [Chlamydomonas reinhardtii]	A8I642	195425.53	98.80	0.64	0.83	0.73
	Predicted protein (Fragment) [Chlamydomonas reinhardtii]	A8JER7	50140.53	96.71	1.58	1.47	1.37
	Predicted protein (Fragment) [Chlamydomonas reinhardtii]	A8JGK4	120750.72	82.61	1.25	1.17	1.07
	Predicted protein (Fragment) [Chlamydomonas reinhardtii]	A8I457	78047.03	64.16	0.75	0.84	0.66
	Predicted protein (Fragment) [Chlamydomonas reinhardtii]	A8J7A3	27309.81	25.97	1.02	0.78	1.08
	Predicted protein (Fragment) [Hordeum vulgare var. distichum]	F2ELH1	52132.50	11.46	0.93	0.99	0.84
	Predicted protein (Fragment) [Micromonas sp. (strain RCC299 / NOUM17)]	C1FJC6	21296.85	68.06	0.33	0.30	0.30
	Predicted protein (Fragment) [Ostreococcus lucimarinus (strain CCE9901)]	A4RZC5	98005.77	87.08	1.02	1.12	1.10
	Predicted protein (Fragment) [Physcomitrella patens subsp. Patens]	A9U6Z4	74946.52	14.22	0.74	0.73	0.79
	Predicted protein (Fragment) [Physcomitrella patens subsp. Patens]	A9TH67	21240.41	57.40	1.17	1.08	1.15
	Predicted protein (Fragment) [Physcomitrella patens subsp. Patens]	A9RZR1	43763.29	97.75	1.02	1.01	1.05
	Predicted protein (Fragment)[Populus trichocarpa]	B9P5C1	47214.99	72.05	0.85	0.90	0.79
	Predicted protein [Arabidopsis lyrata subsp. Lyrata]	D7LLD1	93764.93	96.72	1.22	1.32	1.22
	Predicted protein [Arabidopsis lyrata subsp. Lyrata]	D7KIG2	22482.81	39.27	2.51	2.24	2.48
	Predicted protein [Arabidopsis lyrata subsp. Lyrata]	D7KEX0	25663.67	94.98	0.53	0.51	0.55
	Predicted protein [Arabidopsis lyrata subsp. Lyrata]	D7MPB7	46581.82	52.75	1.00	0.93	0.96

	Predicted protein [Chlamydomonas reinhardtii]	A8J4I9	31175.11	89.83	1.14	1.16	1.11
	Predicted protein [Chlamydomonas reinhardtii]	A8IZC8	260516.48	66.55	0.77	0.72	0.71
	Predicted protein [Chlamydomonas reinhardtii]	A8IAV8	22287.63	67.91	0.84	0.79	0.82
	Predicted protein [Chlamydomonas reinhardtii]	A8I9V9	211046.56	41.33	1.82	1.75	1.87
	Predicted protein [Chlamydomonas reinhardtii]	A8I9P9	83712.02	49.72	1.56	1.52	1.31
	Predicted protein [Chlamydomonas reinhardtii]	A8ITN1	180338.09	72.37	0.87	1.00	0.90
	Predicted protein [Chlamydomonas reinhardtii]	A8IYS7	59858.69	90.55	1.18	1.35	1.53
	Predicted protein [Chlamydomonas reinhardtii]	A8J1Y7	140854.45	99.31	1.29	1.45	1.27
	Predicted protein [Chlamydomonas reinhardtii]	A8HXL6	69399.72	48.90	1.04	1.04	1.17
	Predicted protein [Chlamydomonas reinhardtii]	A8IZH4	72803.55	99.13	1.34	1.40	1.25
	Predicted protein [Chlamydomonas reinhardtii]	A8J0H9	26857.32	39.27	0.61	0.54	0.44
	Predicted protein [Chlamydomonas reinhardtii]	A8JAY2	434031.28	99.95	0.71	0.67	0.64
	Predicted protein[Hordeum vulgare var. distichum]	F2EI36	49418.76	98.79	0.49	0.40	0.21
	Predicted protein [Hordeum vulgare var. distichum]	F2EEF9	26155.73	98.28	0.64	0.56	0.57
	Predicted protein [Hordeum vulgare var. distichum]	F2DXP2	42984.78	92.55	0.78	0.78	0.76
	Predicted protein [Hordeum vulgare var. distichum]	F2DKH8	61941.85	92.37	1.42	1.19	1.38
	Predicted protein [Hordeum vulgare var. distichum]	F2DJ79	38044.75	91.64	0.59	0.71	0.60
	Predicted protein [Hordeum vulgare var. distichum]	F2DAB8	97528.25	69.72	0.00	0.00	0.00
	Predicted protein [Hordeum vulgare var. distichum]	F2E7M0	63830.73	68.42	2.04	2.31	2.28
	Predicted protein [Hordeum vulgare var. distichum]	F2EB44	83320.91	36.26	0.95	0.95	0.96
	Predicted protein [Hordeum vulgare var. distichum]	F2DV46	98384.16	0.00	0.74	0.71	0.88
	Predicted protein [Hordeum vulgare var. distichum]	F2DPP2	40256.89	14.22	0.82	0.86	0.96

	Predicted protein [Hordeum vulgare var. distichum]	F2DR49	52450.83	6.86	0.70	0.75	0.79
	Predicted protein [Hordeum vulgare var. distichum]	F2DYX8	103402.05	4.64	1.12	1.50	1.45
	Predicted protein [Micromonas pusilla (strain CCMP1545)]	C1MIQ4	61029.74	25.97	0.74	0.65	0.78
	Predicted protein [Micromonas pusilla (strain CCMP1545)]	C1MNJ5	86089.91	92.80	0.00	0.00	0.00
	Predicted protein [Micromonas pusilla (strain CCMP1545)]	C1MTC3	70286.62	22.13	0.89	0.87	0.86
	Predicted protein [Micromonas pusilla (strain CCMP1545)]	C1MZN5	185762.91	87.19	0.81	0.85	0.74
	Predicted protein [Micromonas pusilla (strain CCMP1545)]	C1N5A2	45272.02	52.32	0.71	0.63	0.71
	Predicted protein [Micromonas pusilla (strain CCMP1545)]	C1N5H9	366780.31	12.85	0.71	0.67	0.65
	Predicted protein [Micromonas pusilla (strain CCMP1545)]	C1MH65	86921.46	87.02	1.51	1.51	1.52
	Predicted protein [Micromonas pusilla (strain CCMP1545)]	C1MJ56	575836.19	94.27	1.25	1.24	1.24
	Predicted protein [Micromonas pusilla (strain CCMP1545)]	C1N0A5	130564.98	97.99	0.76	0.72	0.85
	Predicted protein [Micromonas pusilla (strain CCMP1545)]	C1N327	35103.54	49.84	0.69	0.63	0.70
	Predicted protein [Micromonas pusilla (strain CCMP1545)]	C1N614	58962.35	48.43	0.49	0.51	0.50
	Predicted protein [Micromonas pusilla (strain CCMP1545)]	C1MRV5	14603.28	92.41	1.01	1.05	1.03
	Predicted protein [Micromonas pusilla (strain CCMP1545)]	C1MVI9	63731.62	77.02	0.63	0.51	0.67
	Predicted protein [Micromonas pusilla (strain CCMP1545)]	C1MXA3	177415.30	81.95	1.26	1.16	1.24
	Predicted protein [Micromonas pusilla (strain CCMP1545)]	C1MZ05	66224.61	3.09	1.27	1.28	1.32
	Predicted protein [Micromonas pusilla (strain CCMP1545)]	C1N6M5	19155.40	9.76	1.10	1.17	1.15
	Predicted protein [Micromonas pusilla (strain CCMP1545)]	C1N3Y0	59138.68	0.37	1.26	1.16	1.24
	Predicted protein [Micromonas pusilla (strain CCMP1545)]	C1N9T4	84004.81	95.02	1.27	1.38	1.49
	Predicted protein [Micromonas sp. (strain RCC299 / NOUM17)]	C1E745	223150.81	3.58	0.50	0.55	0.47
	Predicted protein [Micromonas sp. (strain RCC299 / NOUM17)]	C1EA17	82327.93	62.30	0.67	0.57	0.60

	Predicted protein [Micromonas sp. (strain RCC299 / NOUM17)]	C1FFW0	60948.45	32.64	1.01	1.12	1.15
	Predicted protein [Micromonas sp. (strain RCC299 / NOUM17)]	C1FJB1	590948.75	59.38	0.89	1.03	0.86
	Predicted protein [Micromonas sp. (strain RCC299 / NOUM17)]	C1EH81	119772.70	97.39	1.10	1.09	1.02
	Predicted protein [Micromonas sp. (strain RCC299 / NOUM17)]	C1E9B3	21310.22	78.65	0.77	0.77	0.66
	Predicted protein [Micromonas sp. (strain RCC299 / NOUM17)]	C1EE17	22369.99	32.80	1.83	2.04	1.93
	Predicted protein [Micromonas sp. (strain RCC299 / NOUM17)]	C1FDY1	46403.78	5.07	0.60	0.40	0.62
	Predicted protein [Micromonas sp. (strain RCC299 / NOUM17)]	C1E5A4	36557.32	78.30	0.65	0.62	0.62
	Predicted protein [Micromonas sp. (strain RCC299 / NOUM17)]	C1E482	92274.71	98.58	0.73	0.42	0.72
	Predicted protein [Micromonas sp. (strain RCC299 / NOUM17)]	C1FFE0	32266.11	31.55	0.85	0.80	0.85
	Predicted protein [Micromonas sp. (strain RCC299 / NOUM17)]	C1FG07	97802.95	34.18	0.64	0.51	0.64
	Predicted protein [Micromonas sp. (strain RCC299 / NOUM17)]	C1EEW9	33010.21	18.65	0.89	0.85	0.80
	Predicted protein [Micromonas sp. (strain RCC299 / NOUM17)]	C1EG68	131343.52	89.55	1.55	1.79	1.50
	Predicted protein [Micromonas sp. (strain RCC299 / NOUM17)]	C1EHE3	41350.04	10.18	0.83	0.96	0.93
	Predicted protein [Micromonas sp. (strain RCC299 / NOUM17)]	C1FHA0	60633.64	64.65	0.66	0.51	0.69
	Predicted protein [Micromonas sp. (strain RCC299 / NOUM17)]	C1EII5	42263.76	70.39	1.25	1.15	1.35
	Predicted protein [Micromonas sp. (strain RCC299 / NOUM17)]	C1E2E6	269806.53	44.87	0.76	0.67	0.76
	Predicted protein [Micromonas sp. (strain RCC299 / NOUM17)]	C1E343	132999.58	66.34	0.66	0.71	0.74
	Predicted protein [Micromonas sp. (strain RCC299 / NOUM17)]	C1E3E7	142906.97	20.17	0.37	0.32	0.33
	Predicted protein [Ostreococcus lucimarinus (strain CCE9901)]	A4RQJ4	64621.95	4.96	0.71	0.62	0.73
	Predicted protein [Ostreococcus lucimarinus (strain CCE9901)]	A4RWB2	124386.79	51.65	1.28	1.12	1.16
	Predicted protein [Ostreococcus lucimarinus (strain CCE9901)]	A4S469	51531.53	80.53	0.00	0.00	0.00
	Predicted protein [Ostreococcus lucimarinus (strain CCE9901)]	A4RQE0	24619.01	70.73	0.60	0.40	0.62

	Predicted protein [Ostreococcus lucimarinus (strain CCE9901)]	A4RUD6	41046.13	79.75	0.85	0.80	0.84
	Predicted protein [Physcomitrella patens subsp. Patens]	A9SQ50	20133.12	78.90	0.84	0.80	0.81
	Predicted protein [Physcomitrella patens subsp. Patens]	A9SV58	37644.26	0.37	0.85	0.82	0.89
	Predicted protein [Physcomitrella patens subsp. Patens]	A9TS24	22048.35	99.64	1.03	1.21	0.97
	Predicted protein [Physcomitrella patens subsp. Patens]	A9RVJ1	66666.43	79.30	0.46	0.51	0.46
	Predicted protein [Physcomitrella patens subsp. Patens]	A9S5Z5	37226.09	95.60	1.69	1.70	1.68
	Predicted protein [Physcomitrella patens subsp. Patens]	A9T622	39184.47	49.49	0.89	0.84	0.86
	Predicted protein [Physcomitrella patens subsp. Patens]	A9TT99	148189.50	39.13	1.03	1.05	0.94
	Predicted protein [Physcomitrella patens subsp. Patens]	A9TVX8	14841.82	70.93	0.41	0.43	0.35
	Predicted protein [Physcomitrella patens subsp. Patens]	A9S7R6	46141.72	72.87	1.04	0.94	1.06
	Predicted protein [Physcomitrella patens subsp. Patens]	A9SMY7	92065.03	70.61	1.01	1.18	0.97
	Predicted protein [Physcomitrella patens subsp. Patens]	A9SR52	50533.50	70.19	1.21	1.32	1.33
	Predicted protein [Physcomitrella patens subsp. Patens]	A9RL59	72548.86	34.78	1.03	1.20	1.12
	Predicted protein [Physcomitrella patens subsp. Patens]	A9RFN3	38111.00	8.93	0.50	0.56	0.53
	Predicted protein [Physcomitrella patens subsp. Patens]	A9S7B2	29484.34	15.59	0.70	0.70	0.73
	Predicted protein [Physcomitrella patens subsp. Patens]	A9SLN1	14175.30	46.62	0.70	0.75	0.79
	Predicted protein [Physcomitrella patens subsp. Patens]	A9SPH9	96098.57	67.01	0.82	0.81	0.80
	Predicted protein [Physcomitrella patens subsp. Patens]	A9SSY1	31307.31	70.67	0.60	0.40	0.62
	Predicted protein [Physcomitrella patens subsp. Patens]	A9SY06	406725.25	98.83	1.17	1.24	1.09
	Predicted protein [Physcomitrella patens subsp. Patens]	A9TC80	123971.12	33.11	1.51	1.39	1.31
	Predicted protein [Physcomitrella patens subsp. Patens]	A9TFK1	28794.15	28.16	0.96	1.09	0.93
	Predicted protein [Physcomitrella patens subsp. Patens]	A9TIZ3	147193.88	57.11	0.82	0.69	0.76

	Predicted protein [Physcomitrella patens subsp. Patens]	A9TRI9	38199.96	68.51	0.64	0.79	0.74
	Predicted protein [Physcomitrella patens subsp. Patens]	A9TVS1	43327.08	33.87	0.60	0.50	0.60
	Predicted protein [Physcomitrella patens subsp. Patens]	A9TXK3	26634.20	47.23	0.49	0.51	0.50
	Predicted protein [Populus trichocarpa]	B9MUF5	126761.82	47.83	1.39	1.47	1.50
	Predicted protein [Populus trichocarpa]	B9N4L9	22068.65	11.82	1.00	0.89	1.01
	Predicted protein [Populus trichocarpa]	B9NKC4	30362.08	76.10	0.69	0.56	0.67
	Predicted protein [Populus trichocarpa]	B9P7U7	10687.54	78.30	0.92	0.83	0.88
	Predicted protein [Populus trichocarpa]	B9N384	18942.36	74.05	0.81	0.70	0.86
	Predicted protein [Populus trichocarpa]	B9PER2	30649.19	75.99	0.69	0.68	0.77
	Putative uncharacterized protein (Fragment) [Medicago truncatula]	B7FKU6	34789.80	85.57	0.67	0.73	0.72
	Putative uncharacterized protein (Fragment) [Panax quinquefolius]	A9LIX2	41787.36	100.00	0.84	0.84	0.88
	Putative uncharacterized protein (Fragment) [Panax quinquefolius]	A9QMD5	32121.98	90.19	0.18	1.08	1.06
	Putative uncharacterized protein (Fragment) [Ricinus communis]	B9TKY0	37713.68	97.88	1.03	0.99	0.99
	Putative uncharacterized protein (Fragment) [Ricinus communis]	B9TLQ3	26220.33	26.82	2.17	2.09	1.94
	Putative uncharacterized protein (Fragment) [Selaginella moellendorffii]	D8S333	37403.13	66.63	0.77	0.69	0.73
	Putative uncharacterized protein (Fragment) [Selaginella moellendorffii]	D8S5R4	42267.39	91.96	0.57	0.61	0.53
	Putative uncharacterized protein (Fragment) [Selaginella moellendorffii]	D8RJR2	34567.49	64.16	1.27	1.35	1.23
	Putative uncharacterized protein (Fragment) [Selaginella moellendorffii]	D8T9A1	37150.94	72.75	1.43	1.40	1.35
	Putative uncharacterized protein (Fragment) [Selaginella moellendorffii]	D8QWI8	62333.26	62.12	1.40	1.43	1.38
	Putative uncharacterized protein	A5C235	46756.27	41.33	1.13	1.05	1.03

	(Fragment) [Vitis vinifera]						
	Putative uncharacterized protein (Fragment) [Volvox carteri]	D8UE20	124129.57	14.02	0.65	0.55	0.51
	Putative uncharacterized protein (Fragment) [Volvox carteri]	D8TSL2	47513.86	37.43	0.68	0.58	0.72
	Putative uncharacterized protein ACA9A-1 [Selaginella moellendorffii]	D8RQT1	128945.45	42.67	1.22	1.07	1.06
	Putative uncharacterized protein At2g32550/T26B15.11 [Arabidopsis thaliana]	Q8L8C5	36351.11	67.54	0.74	0.60	0.76
	Putative uncharacterized protein At2g38790 [Arabidopsis thaliana]	Q9SII2	25767.19	29.31	0.87	0.97	0.87
	Putative uncharacterized protein AT4g19320 [Arabidopsis thaliana]	O65703	92858.60	34.48	0.00	0.00	0.00
	Putative uncharacterized protein B1077E08.16 [Oryza sativa subsp. Japonica]	Q5Z5M6	14441.29	90.04	0.75	0.72	0.76
	Putative uncharacterized protein B1114B07.29 [Oryza sativa subsp. Japonica]	Q8S1A6	71479.93	89.54	0.57	0.61	0.53
	Putative uncharacterized protein B1157F01.25 [Oryza sativa subsp. Japonica]	Q6YT95	13257.21	38.85	0.29	0.24	0.29
	Putative uncharacterized protein B1460A05.13 [Oryza sativa subsp. Japonica]	Q5VMF6	18480.16	9.35	1.14	1.10	1.15
	Putative uncharacterized protein OJ1115_A05.16 [Oryza sativa subsp. Japonica]	Q6EUN3	21109.78	24.94	0.63	0.63	0.53
	Putative uncharacterized protein OJ1116_A06.32 [Oryza sativa subsp. Japonica]	Q6H706	17490.54	42.14	1.33	1.23	1.29
	Putative uncharacterized protein OJ1163_G08.41 [Oryza sativa subsp. Japonica]	Q7EZS4	7206.81	60.61	0.88	0.92	0.93
	Putative uncharacterized protein OJ1409_C08.15 [Oryza sativa subsp. Japonica]	Q7XIW2	7675.97	37.86	1.02	0.77	0.81
	Putative uncharacterized protein OJ1793_E11.116 [Oryza sativa subsp. Japonica]	Q8H516	35785.43	24.60	0.63	0.63	0.53
	Putative uncharacterized protein [Arabidopsis lyrata subsp. Lyrata]	D7KDZ0	42497.21	82.00	1.40	1.42	1.35
	Putative uncharacterized protein	D7LLV0	102125.12	35.97	0.63	0.63	0.53

	[Arabidopsis lyrata subsp. Lyrata]						
	Putative uncharacterized protein [Arabidopsis lyrata subsp. Lyrata]	D7KPV3	64852.32	74.27	1.86	1.43	1.43
	Putative uncharacterized protein [Arabidopsis lyrata subsp. Lyrata]	D7KUN7	130545.12	92.05	1.01	0.98	0.95
	Putative uncharacterized protein [Arabidopsis lyrata subsp. Lyrata]	D7LAC6	26998.77	84.50	0.68	0.70	0.69
	Putative uncharacterized protein [Arabidopsis lyrata subsp. Lyrata]	D7LEA5	79801.54	97.12	1.13	1.09	1.08
	Putative uncharacterized protein [Arabidopsis lyrata subsp. Lyrata]	D7LTZ3	13092.47	55.29	2.10	2.05	2.23
	Putative uncharacterized protein [Arabidopsis thaliana]	Q0WT58	44335.83	76.97	0.96	1.10	0.99
	Putative uncharacterized protein [Chlorella variabilis]	E1Z3L3	114041.69	89.66	0.38	0.30	0.36
	Putative uncharacterized protein [Chlorella variabilis]	E1ZHC3	302006.34	99.34	1.17	1.25	1.20
	Putative uncharacterized protein [Chlorella variabilis]	E1ZIR4	23813.81	84.57	1.12	0.95	0.95
	Putative uncharacterized protein [Chlorella variabilis]	E1ZK54	265646.16	24.25	0.59	0.72	0.88
	Putative uncharacterized protein [Chlorella variabilis]	E1ZKV2	70791.45	82.65	0.52	0.51	0.56
	Putative uncharacterized protein [Chlorella variabilis]	E1ZLS5	21287.42	55.09	1.33	1.34	1.46
	Putative uncharacterized protein [Chlorella variabilis]	E1Z438	43163.49	39.83	1.17	1.05	1.10
	Putative uncharacterized protein [Chlorella variabilis]	E1ZSR5	60378.57	86.50	1.09	1.23	1.16
	Putative uncharacterized protein [Chlorella variabilis]	E1ZCZ1	28672.54	88.24	1.08	1.36	1.04
	Putative uncharacterized protein [Chlorella variabilis]	E1ZEV1	22700.71	94.02	1.58	1.68	1.62
	Putative uncharacterized protein [Chlorella variabilis]	E1ZSA5	90555.33	12.63	0.76	0.75	0.68
	Putative uncharacterized protein [Chlorella variabilis]	E1ZED6	48712.36	75.32	0.82	0.84	0.63
	Putative uncharacterized protein [Chlorella variabilis]	E1Z8J4	54442.38	30.12	1.58	1.62	1.49
	Putative uncharacterized protein [Chlorella variabilis]	E1ZP78	60660.19	96.10	1.23	1.20	1.19
	Putative uncharacterized protein [Chlorella variabilis]	E1ZP85	48322.38	49.84	0.81	0.79	0.78

	Putative uncharacterized protein [Chlorella variabilis]	E1ZQA8	84716.31	99.78	0.58	0.66	0.69
	Putative uncharacterized protein [Hydrocotyle rotundifolia]	O79353	36786.92	99.86	0.50	1.03	0.81
	Putative uncharacterized protein [Medicago truncatula]	G7IGK8	45540.24	85.23	0.80	0.79	0.91
	Putative uncharacterized protein [Medicago truncatula]	G7JFQ1	15489.22	78.30	1.04	1.01	1.06
	Putative uncharacterized protein [Medicago truncatula]	G7JPL0	20667.46	73.80	0.79	0.91	0.75
	Putative uncharacterized protein [Medicago truncatula]	G7JLE2	37098.62	52.86	0.89	0.96	0.92
	Putative uncharacterized protein [Medicago truncatula]	G7JD15	14224.70	85.57	1.84	1.66	1.70
	Putative uncharacterized protein [Medicago truncatula]	G7K537	29406.96	62.56	0.76	0.65	0.67
	Putative uncharacterized protein [Medicago truncatula]	A2Q4I1	18716.88	1.29	1.30	1.21	1.12
	Putative uncharacterized protein [Medicago truncatula]	B7FJ40	20410.09	76.16	1.07	1.08	1.08
	Putative uncharacterized protein [Micromonas pusilla (strain CCMP1545)]	C1MJ61	112136.65	99.90	1.49	1.35	1.65
	Putative uncharacterized protein[Micromonas sp. (strain RCC299 / NOUM17)]	C1EFQ2	75790.99	73.61	1.39	1.18	1.25
	Putative uncharacterized protein [Micromonas sp. (strain RCC299 / NOUM17)]	C1FJJ6	141337.13	2.64	1.59	1.86	1.51
	Putative uncharacterized protein [Oryza sativa subsp. Indica]	A2WNM3	48836.04	37.57	1.47	1.36	1.27
	Putative uncharacterized protein [Oryza sativa subsp. Indica]	B8ACE6	34861.89	37.43	1.13	0.99	0.91
	Putative uncharacterized protein [Oryza sativa subsp. Indica]	A2X346	45427.95	39.41	2.51	2.24	2.48
	Putative uncharacterized protein [Oryza sativa subsp. Indica]	A2X751	52635.81	35.67	0.83	0.78	0.73
	Putative uncharacterized protein [Oryza sativa subsp. Indica]	A2XDQ4	52198.74	91.54	0.87	0.83	0.85
	Putative uncharacterized protein [Oryza sativa subsp. Indica]	B8AJP4	44503.96	66.63	1.48	1.55	1.31
	Putative uncharacterized protein [Oryza sativa subsp. Indica]	B8ASH1	52763.95	95.45	0.74	0.73	0.75
	Putative uncharacterized protein	B8ATA9	89660.78	63.32	1.22	1.33	1.26

	[Oryza sativa subsp. Indica]						
	Putative uncharacterized protein [Oryza sativa subsp. Indica]	A2XZA0	200974.11	76.55	0.62	0.58	0.62
	Putative uncharacterized protein [Oryza sativa subsp. Indica]	A2Y6G0	13432.15	10.80	0.83	0.96	0.93
	Putative uncharacterized protein [Oryza sativa subsp. Indica]	B8B1Z2	46439.17	53.29	0.66	0.97	0.94
	Putative uncharacterized protein [Oryza sativa subsp. Indica]	A2YEY3	49515.69	30.76	1.64	1.78	1.81
	Putative uncharacterized protein [Oryza sativa subsp. Indica]	B8B8R0	76478.16	7.87	0.86	1.09	1.26
	Putative uncharacterized protein [Oryza sativa subsp. Indica]	A2YYY1	30745.73	35.08	0.71	0.67	0.69
	Putative uncharacterized protein [Oryza sativa subsp. Indica]	A2YZ32	42514.51	75.77	1.68	1.99	1.56
	Putative uncharacterized protein [Oryza sativa subsp. Indica]	B8BEX3	39799.09	64.08	0.90	0.69	0.76
	Putative uncharacterized protein [Oryza sativa subsp. Indica]	A2ZL70	13443.74	69.56	0.80	1.24	1.10
	Putative uncharacterized protein [Oryza sativa subsp. Indica]	B8BMU0	139147.59	43.98	1.22	1.19	1.09
	Putative uncharacterized protein [Oryza sativa subsp. japonica]	Q10HF9	9599.80	48.67	1.01	0.99	0.91
	Putative uncharacterized protein [Oryza sativa subsp. japonica]	Q33AG0	12904.58	88.00	0.78	0.74	0.76
	Putative uncharacterized protein [Oryza sativa subsp. japonica]	Q2QU08	8396.24	22.84	0.64	0.67	0.68
	Putative uncharacterized protein [Oryza sativa subsp. japonica]	A3AA68	18810.54	33.72	1.19	1.11	1.15
	Putative uncharacterized protein [Oryza sativa subsp. japonica]	A3BA72	25044.12	82.00	0.52	0.72	0.65
	Putative uncharacterized protein [Oryza sativa subsp. japonica]	B9G1R3	31767.17	55.60	0.48	0.52	0.49
	Putative uncharacterized protein [Oryza sativa subsp. japonica]	A3C3B6	15922.61	3.53	1.48	1.55	1.31
	Putative uncharacterized protein [Oryza sativa subsp. japonica]	A3CBZ4	37577.08	65.69	1.12	1.44	1.27
	Putative uncharacterized protein [Oryza sativa subsp. japonica]	B9GDF5	47149.99	85.13	0.59	0.71	0.60
	Putative uncharacterized protein [Oryza sativa subsp. japonica]	A3CJ83	84865.37	99.27	1.75	1.87	1.95
	Putative uncharacterized protein [Oryza sativa subsp. japonica]	Q6UU23	25800.26	24.25	0.74	0.71	0.88

	Putative uncharacterized protein [Picea sitchensis]	A9NWM1	81495.68	96.66	0.85	0.65	0.75
	Putative uncharacterized protein [Picea sitchensis]	A9NVS6	46443.22	71.66	0.67	0.56	0.59
	Putative uncharacterized protein [Picea sitchensis]	A9NY73	57308.86	27.49	1.35	1.35	1.52
	Putative uncharacterized protein [Picea sitchensis]	A9NMF7	8354.28	49.72	0.94	1.09	0.86
	Putative uncharacterized protein [Ricinus communis]	B9SD84	10359.28	4.86	0.80	0.77	0.90
	Putative uncharacterized protein [Ricinus communis]	B9RW68	23922.83	75.33	0.73	0.77	0.71
	Putative uncharacterized protein [Ricinus communis]	B9R7X9	35017.68	83.50	0.61	0.47	0.62
	Putative uncharacterized protein [Ricinus communis]	B9R8R2	90915.91	64.57	1.21	1.13	1.04
	Putative uncharacterized protein [Ricinus communis]	B9TJ37	11746.95	82.92	0.91	0.99	0.82
	Putative uncharacterized protein [Ricinus communis]	B9TQ77	10430.20	15.98	0.63	0.55	0.63
	Putative uncharacterized protein [Selaginella moellendorffii]	D8T035	111924.80	15.59	0.84	0.87	0.77
	Putative uncharacterized protein [Selaginella moellendorffii]	D8RW89	51263.90	30.92	1.51	1.39	1.31
	Putative uncharacterized protein [Selaginella moellendorffii]	D8R5G8	21047.96	95.79	0.93	0.94	0.88
	Putative uncharacterized protein [Selaginella moellendorffii]	D8SWD6	243261.91	99.65	0.59	0.50	0.60
	Putative uncharacterized protein [Selaginella moellendorffii]	D8S3I4	108871.70	5.95	0.73	0.57	0.63
	Putative uncharacterized protein [Selaginella moellendorffii]	D8S410	27016.93	65.46	1.43	1.23	1.35
	Putative uncharacterized protein [Selaginella moellendorffii]	D8S4W9	68461.99	46.87	0.57	0.48	0.59
	Putative uncharacterized protein [Selaginella moellendorffii]	D8S912	37074.16	37.89	1.10	1.17	1.15
	Putative uncharacterized protein [Selaginella moellendorffii]	D8SXP2	169984.23	21.04	0.57	0.65	0.54
	Putative uncharacterized protein [Selaginella moellendorffii]	D8T0R3	50211.76	59.69	1.09	1.12	1.07
	Putative uncharacterized protein [Selaginella moellendorffii]	D8T211	55294.64	4.86	0.85	0.76	0.71
	Putative uncharacterized protein [Selaginella moellendorffii]	D8T2J6	59548.21	99.95	0.82	1.00	0.89

	Putative uncharacterized protein [Selaginella moellendorffii]	D8QWA1	23878.99	91.48	1.36	1.44	1.24
	Putative uncharacterized protein [Selaginella moellendorffii]	D8R433	82928.05	21.04	0.86	0.87	0.84
	Putative uncharacterized protein [Selaginella moellendorffii]	D8R5S4	61229.26	8.09	0.97	1.11	0.92
	Putative uncharacterized protein [Selaginella moellendorffii]	D8RP87	107867.17	39.44	1.59	1.36	1.49
	Putative uncharacterized protein [Selaginella moellendorffii]	D8RR30	86813.24	1.97	0.93	0.90	0.87
	Putative uncharacterized protein [Vitis vinifera]	F6HJ96	46924.18	26.99	0.94	0.88	0.88
	Putative uncharacterized protein [Vitis vinifera]	D7U571	67242.48	42.41	1.10	1.17	1.15
	Putative uncharacterized protein [Vitis vinifera]	F6H3I8	59065.47	80.17	0.75	0.78	0.86
	Putative uncharacterized protein [Vitis vinifera]	F6H3W0	79003.05	85.09	0.92	0.92	0.90
	Putative uncharacterized protein [Vitis vinifera]	D7TI71	28464.37	90.68	1.58	1.62	1.50
	Putative uncharacterized protein [Vitis vinifera]	F6I3N6	25016.55	16.17	0.87	0.92	0.88
	Putative uncharacterized protein [Vitis vinifera]	F6GXW6	109219.59	62.30	0.90	0.75	0.89
	Putative uncharacterized protein [Vitis vinifera]	F6HY27	37487.58	63.49	0.83	0.81	0.73
	Putative uncharacterized protein [Vitis vinifera]	D7T2X2	44916.57	80.30	0.82	0.85	0.83
	Putative uncharacterized protein [Vitis vinifera]	F6HUX3	65207.43	72.75	1.27	1.39	1.31
	Putative uncharacterized protein [Vitis vinifera]	D7T7Q7	48793.64	30.12	1.02	0.77	0.81
	Putative uncharacterized protein [Vitis vinifera]	F6H0I7	65614.42	59.97	1.20	1.09	1.22
	Putative uncharacterized protein [Vitis vinifera]	E0CQP4	34053.86	78.40	0.92	0.96	0.89
	Putative uncharacterized protein [Vitis vinifera]	F6GYR0	57450.76	44.10	0.61	0.66	0.54
	Putative uncharacterized protein [Vitis vinifera]	F6H211	233695.53	49.95	1.00	0.96	0.94
	Putative uncharacterized protein [Vitis vinifera]	D7SWZ1	71931.53	19.95	1.58	1.47	1.52
	Putative uncharacterized protein [Vitis vinifera]	A5B5I1	41861.93	57.52	0.76	0.76	0.73

	Putative uncharacterized protein [Vitis vinifera]	A5BMA9	52908.24	81.75	0.93	1.00	0.88
	Putative uncharacterized protein [Vitis vinifera]	A5B097	63247.52	84.21	0.96	0.97	0.96
	Putative uncharacterized protein [Vitis vinifera]	A5BX68	31696.33	99.47	1.19	0.73	0.92
	Putative uncharacterized protein [Vitis vinifera]	A5BF32	67618.68	67.76	1.56	1.45	1.49
	Putative uncharacterized protein [Vitis vinifera]	A5B460	154338.17	25.46	0.86	0.78	0.89
	Putative uncharacterized protein [Vitis vinifera]	A5AH96	71784.73	99.05	0.87	0.80	0.89
	Putative uncharacterized protein [Vitis vinifera]	A5C6D8	259849.53	34.63	1.05	1.00	1.10
	Putative uncharacterized protein [Vitis vinifera]	A5B921	273022.09	97.55	1.16	1.13	1.12
	Putative uncharacterized protein [Vitis vinifera]	A5APM4	71189.02	97.91	0.52	0.33	0.41
	Putative uncharacterized protein [Vitis vinifera]	A5BSU3	15195.70	85.73	1.12	1.02	1.02
	Putative uncharacterized protein [Vitis vinifera]	A5AVY1	38413.15	37.17	1.15	1.05	1.08
	Putative uncharacterized protein [Vitis vinifera]	A5B364	37279.04	91.38	0.69	0.67	0.64
	Putative uncharacterized protein [Vitis vinifera]	A5BL95	183257.03	29.79	0.72	0.82	0.70
	Putative uncharacterized protein [Vitis vinifera]	A5BD78	210701.19	98.66	1.13	1.53	1.20
	Putative uncharacterized protein [Vitis vinifera]	A5BKS7	300045.66	63.66	0.72	0.60	0.67
	Putative uncharacterized protein [Volvox carteri]	D8TGT0	71364.09	83.24	1.02	1.00	1.08
	Putative uncharacterized protein [Volvox carteri]	D8TQ61	209386.56	13.23	1.21	1.42	0.99
	Putative uncharacterized protein [Volvox carteri]	D8TQI6	168751.95	88.98	0.89	0.90	0.94
	Putative uncharacterized protein [Volvox carteri]	D8U0P7	141080.36	40.11	1.80	1.78	1.71
	Putative uncharacterized protein [Volvox carteri]	D8U1S9	159267.77	50.98	1.26	1.16	1.24
	Putative uncharacterized protein [Volvox carteri]	D8UDH7	206769.11	59.88	0.51	0.47	0.46
	Putative uncharacterized protein [Volvox carteri]	D8UJY1	237804.30	99.99	1.43	1.45	1.52

	Putative uncharacterized protein [Volvox carteri]	D8TLM2	92855.96	91.06	1.03	1.07	1.07
	Putative uncharacterized protein [Volvox carteri]	D8TPF4	192332.66	99.64	0.89	0.78	0.85
	Putative uncharacterized protein [Volvox carteri]	D8UDG6	337132.50	0.37	0.91	0.99	0.82
	Putative uncharacterized protein [Volvox carteri]	D8TKM0	304909.72	99.61	1.36	1.25	1.18
	Putative uncharacterized protein [Volvox carteri]	D8TJH8	97147.13	55.60	1.07	1.11	1.10
	Putative uncharacterized protein [Volvox carteri]	D8TSH0	613538.25	99.81	0.59	0.53	0.60
	Putative uncharacterized protein [Volvox carteri]	D8TT61	161567.13	37.14	0.65	0.66	0.58
	Putative uncharacterized protein [Volvox carteri]	D8TWY6	97959.94	84.57	0.70	0.67	0.69
	Putative uncharacterized protein [Volvox carteri]	D8TYW7	28859.65	61.06	0.46	0.35	0.46
	Putative uncharacterized protein [Volvox carteri]	D8U0U5	33854.71	88.46	0.95	1.04	1.04
	Putative uncharacterized protein [Volvox carteri]	D8U6N4	61216.14	99.68	1.02	0.91	0.97
	Putative uncharacterized protein [Volvox carteri]	D8U7F3	48311.75	21.95	1.21	1.07	1.03
	Putative uncharacterized protein [Volvox carteri]	D8U9I4	25735.01	2.86	1.13	1.11	1.16
	Putative uncharacterized protein [Volvox carteri]	D8UAE7	122620.55	94.11	1.11	1.05	1.09
	Putative uncharacterized protein [Volvox carteri]	D8UBH2	87255.41	99.88	0.91	0.89	0.91
	Putative uncharacterized protein [Volvox carteri]	D8UDA4	65960.89	62.64	0.95	0.90	0.89
	Putative uncharacterized protein [Volvox carteri]	D8UDJ6	187123.23	94.24	1.23	1.41	1.18
	Putative uncharacterized protein [Volvox carteri]	D8UFI1	89716.30	48.07	0.78	0.72	0.84
	Putative uncharacterized protein [Zea mays]	B6U2N7	23312.19	97.81	0.71	0.67	0.75
	Putative uncharacterized protein [Zea mays]	B6TGU7	21760.88	21.41	0.43	0.43	0.42
	Putative uncharacterized protein OSJNBa0064I23.24 [Oryza sativa subsp. Japonica]	Q69KN3	32909.00	34.18	1.40	1.33	1.25
	Putative uncharacterized protein	Q6YX27	18872.56	92.07	1.08	0.98	0.94

	OSJNBa0073J19.34 [Oryza sativa subsp. Japonica]						
	Putative uncharacterized protein P0025A05.11 [Oryza sativa subsp. Japonica]	Q94D24	20675.64	78.25	0.75	0.81	0.65
	Putative uncharacterized protein P0416A11.31 [Oryza sativa subsp. Japonica]	Q67WV1	11227.57	84.92	0.79	0.79	0.77
	Putative uncharacterized protein P0441A12.26 [Oryza sativa subsp. Japonica]	Q69QG0	12094.45	8.30	0.70	0.59	0.64
	Putative uncharacterized protein P0462E11.8 [Oryza sativa subsp. Japonica]	Q6ZCP7	18686.83	75.15	0.77	0.70	0.72
	Putative uncharacterized protein P0698A04.21 [Oryza sativa subsp. Japonica]	Q5ZDS7	27460.91	41.60	0.90	0.91	1.21
	Putative uncharacterized protein Sb0013s006040 [Sorghum bicolor]	C6JRX0	124954.73	86.18	1.21	1.13	1.04
	Putative uncharacterized protein Sb01g023040 [Sorghum bicolor]	C5WMC7	122461.85	98.92	0.79	0.80	0.83
	Putative uncharacterized protein Sb01g034480 [Sorghum bicolor]	C5WYR0	80837.00	94.54	1.21	1.32	1.33
	Putative uncharacterized protein Sb01g040900 [Sorghum bicolor]	C5WQW2	59313.42	32.80	1.22	1.19	1.09
	Putative uncharacterized protein Sb01g048360 [Sorghum bicolor]	C5WZW9	34222.33	70.80	1.04	1.04	1.01
	Putative uncharacterized protein Sb02g011350 [Sorghum bicolor]	C5X5U5	74658.35	97.17	0.44	0.50	0.49
	Putative uncharacterized protein Sb02g029230 [Sorghum bicolor]	C5X4P7	31983.79	44.49	0.53	0.51	0.50
	Putative uncharacterized protein Sb02g034740 [Sorghum bicolor]	C5XA14	97402.43	22.49	0.89	0.87	0.86
	Putative uncharacterized protein Sb03g006485 (Fragment) [Sorghum bicolor]	C5XQ47	22316.22	19.95	1.85	1.71	1.64
	Putative uncharacterized protein Sb03g022150 [Sorghum bicolor]	C5XM41	67623.48	58.94	0.90	0.66	0.91
	Putative uncharacterized protein Sb03g040840 [Sorghum bicolor]	C5XQL5	55615.50	69.00	2.04	2.31	2.28
	Putative uncharacterized protein Sb04g002055 (Fragment) [Sorghum bicolor]	C5XT50	50668.12	60.52	1.42	1.62	1.41
	Putative uncharacterized protein	C5XZG8	46034.26	98.94	0.99	1.35	1.35

	Sb04g009390 [Sorghum bicolor]						
	Putative uncharacterized protein Sb05g004800 [Sorghum bicolor]	C5Y628	45671.47	80.17	0.98	1.15	1.16
	Putative uncharacterized protein Sb05g005410 [Sorghum bicolor]	C5Y682	51166.57	88.43	0.66	0.51	0.69
	Putative uncharacterized protein Sb05g006045 (Fragment) [Sorghum bicolor]	C5Y6T0	95332.10	94.02	1.23	1.06	1.14
	Putative uncharacterized protein Sb06g002100 [Sorghum bicolor]	C5YCN8	59707.22	97.32	0.89	0.91	0.89
	Putative uncharacterized protein Sb09g004210 [Sorghum bicolor]	C5Z0I1	168688.80	76.64	1.22	1.21	1.22
	Putative uncharacterized protein Sb10g007720 [Sorghum bicolor]	C5Z6Y5	18597.74	93.49	0.63	0.67	0.70
	Putative uncharacterized protein Sb10g019620 [Sorghum bicolor]	C5Z356	110799.58	89.35	0.72	0.73	0.84
	Putative uncharacterized protein SmCNGC1_2 [Selaginella moellendorffii]	D8SGT0	84523.99	19.95	1.43	1.30	1.19
	Uncharacterized protein (Fragment) [Genlisea aurea]	S8EB22	230957.64	34.48	0.87	0.94	0.86
	Uncharacterized protein (Fragment) [Genlisea aurea]	S8CV80	45525.45	78.99	0.68	0.63	0.70
	Uncharacterized protein (Fragment) [Genlisea aurea]	S8E6V9	25215.45	46.13	1.38	1.54	0.87
	Uncharacterized protein (Fragment) [Genlisea aurea]	S8DXL6	12752.96	57.89	1.04	0.82	1.17
	Uncharacterized protein (Fragment) [Genlisea aurea]	S8CB80	107586.24	18.08	0.68	0.55	0.59
	Uncharacterized protein (Fragment) [Genlisea aurea]	S8DE45	101943.10	17.32	0.74	0.72	0.87
	Uncharacterized protein (Fragment) [Hordeum vulgare var. distichum]	M0VCF6	26565.38	67.54	1.14	1.18	1.09
	Uncharacterized protein (Fragment) [Oryza glaberrima]	I1Q922	44056.09	49.14	0.93	1.06	0.94
	Uncharacterized protein (Fragment) [Oryza glaberrima]	I1PYL4	125917.70	98.91	0.89	0.93	0.94
	Uncharacterized protein (Fragment) [Oryza glaberrima]	I1QKN1	122739.86	98.40	0.57	0.49	0.56
	Uncharacterized protein (Fragment) [Oryza glaberrima]	I1NQF7	36737.94	78.94	0.95	0.96	0.92
	Uncharacterized protein (Fragment) [Oryza glaberrima]	I1R0R7	71105.33	71.86	1.08	1.34	1.04
	Uncharacterized protein (Fragment)	I1QGC2	113541.56	56.98	0.91	1.05	0.92

	[Oryza glaberrima]						
	Uncharacterized protein (Fragment) [Oryza glaberrima]	I1R5A8	29507.12	49.72	0.99	0.84	1.01
	Uncharacterized protein (Fragment) [Oryza glaberrima]	I1R152	71913.82	43.33	0.97	1.24	0.92
	Uncharacterized protein (Fragment) [Physcomitrella patens subsp. Patens]	A9S9W1	93299.38	52.12	0.66	0.95	0.88
	Uncharacterized protein (Fragment) [Pinus radiata]	H9MC26	9338.01	8.09	0.79	0.76	0.75
	Uncharacterized protein (Fragment) [Populus trichocarpa]	U5FUD2	29683.49	69.21	0.61	0.74	0.72
	Uncharacterized protein (Fragment) [Prunus persica]	M5X4W4	30258.44	85.20	0.78	0.77	0.83
	Uncharacterized protein (Fragment) [Prunus persica]	M5XF06	61534.39	39.13	0.00	0.00	0.00
	Uncharacterized protein (Fragment) [Prunus persica]	M5WQH3	31076.10	0.24	1.39	1.26	1.59
	Uncharacterized protein (Fragment) [Setaria italica]	K4AIQ7	167764.81	83.99	1.05	1.05	0.91
	Uncharacterized protein (Fragment) [Setaria italica]	K4ALM6	43790.97	27.83	1.51	1.43	1.64
	Uncharacterized protein (Fragment) [Triticum urartu]	T1NE66	50020.50	78.60	1.06	1.00	1.02
	Uncharacterized protein (Fragment) [Triticum urartu]	T1MRF1	19602.58	96.05	0.69	0.65	0.69
	Uncharacterized protein (Fragment) [Triticum urartu]	T1N901	34899.50	89.93	0.62	0.57	0.63
	Uncharacterized protein (Fragment) [Triticum urartu]	T1MM71	90645.89	74.21	0.97	0.88	0.96
	Uncharacterized protein (Fragment) [Triticum urartu]	T1NSU1	56584.64	47.71	1.46	1.69	1.70
	Uncharacterized protein (Fragment) [Triticum urartu]	T1L9A1	28573.54	5.73	1.21	1.13	1.04
	Uncharacterized protein At4g04980 [Arabidopsis thaliana]	Q1PEB4	92317.36	30.44	0.63	0.61	0.54
	Uncharacterized protein [Aegilops tauschii]	N1QPE5	33212.18	79.28	0.59	0.71	0.60
	Uncharacterized protein [Aegilops tauschii]	M8B0V7	40839.98	71.07	0.79	0.77	0.68
	Uncharacterized protein [Aegilops tauschii]	M8C2C0	83070.30	54.77	1.00	1.21	0.89
	Uncharacterized protein [Aegilops tauschii]	M8BD46	49257.91	85.89	2.11	1.84	2.00

	Uncharacterized protein [Aegilops tauschii]	R7VZ59	66794.20	65.53	1.08	1.10	1.02
	Uncharacterized protein [Aegilops tauschii]	M8BEY0	96025.16	42.93	1.08	1.02	1.10
	Uncharacterized protein [Aegilops tauschii]	M8CA58	28005.81	37.14	0.55	0.47	0.49
	Uncharacterized protein [Aegilops tauschii]	R7WC32	130442.31	82.45	0.54	0.54	0.59
	Uncharacterized protein [Aegilops tauschii]	N1R1I4	32626.14	43.72	1.05	0.91	1.00
	Uncharacterized protein [Amborella trichopoda]	U5D2D5	14251.31	89.90	1.03	1.01	1.08
	Uncharacterized protein [Amborella trichopoda]	U5D656	71123.84	33.11	1.37	1.54	1.42
	Uncharacterized protein [Arabidopsis thaliana]	F4JMA6	107494.45	7.45	0.70	0.54	0.57
	Uncharacterized protein [Bathycoccus prasinos]	K8E9I9	99141.04	16.94	1.04	1.00	1.06
	Uncharacterized protein [Bathycoccus prasinos]	K8F494	177681.66	62.81	0.56	0.51	0.60
	Uncharacterized protein [Bathycoccus prasinos]	K8EGP0	52808.08	12.83	1.05	1.02	1.12
	Uncharacterized protein [Brachypodium distachyon]	I1GQH0	59957.22	64.41	1.19	1.10	1.19
	Uncharacterized protein [Brachypodium distachyon]	I1H118	26488.57	49.14	1.07	1.18	1.10
	Uncharacterized protein [Brachypodium distachyon]	I1H1V0	33553.03	17.32	0.95	0.97	0.88
	Uncharacterized protein [Brachypodium distachyon]	I1H6V3	134518.58	97.87	0.88	0.83	0.83
	Uncharacterized protein [Brachypodium distachyon]	I1H9U2	48796.43	23.02	0.86	0.88	0.95
	Uncharacterized protein [Brachypodium distachyon]	I1HBJ0	31587.01	78.30	1.20	1.08	1.17
	Uncharacterized protein [Brachypodium distachyon]	I1HP30	81367.98	90.59	0.83	0.68	0.74
	Uncharacterized protein [Brachypodium distachyon]	I1HRZ8	85038.59	31.55	1.12	1.00	1.09
	Uncharacterized protein [Brachypodium distachyon]	I1HVF0	23269.26	52.86	0.89	0.75	0.81
	Uncharacterized protein [Brachypodium distachyon]	I1I9M5	84875.33	71.07	0.60	0.56	0.58
	Uncharacterized protein [Brachypodium distachyon]	I1IAL4	88819.16	7.59	0.75	0.71	0.69

	Uncharacterized protein [Brachypodium distachyon]	I1IGR5	35700.11	15.79	0.84	0.74	0.83
	Uncharacterized protein [Brachypodium distachyon]	I1IKN3	50374.93	78.30	1.01	1.17	1.14
	Uncharacterized protein [Brachypodium distachyon]	I1J2W5	47385.92	47.71	0.72	0.67	0.72
	Uncharacterized protein [Brassica rapa subsp. Pekinensis]	M4CG04	23733.97	23.73	0.58	0.56	0.60
	Uncharacterized protein [Brassica rapa subsp. Pekinensis]	M4CIQ7	49076.36	63.49	1.09	1.28	1.06
	Uncharacterized protein [Brassica rapa subsp. Pekinensis]	M4CJL5	27589.63	44.36	0.92	0.81	0.83
	Uncharacterized protein [Brassica rapa subsp. Pekinensis]	M4CN10	51098.29	62.90	0.68	0.58	0.69
	Uncharacterized protein [Brassica rapa subsp. Pekinensis]	M4CNA0	37961.73	6.16	1.33	1.23	1.29
	Uncharacterized protein [Brassica rapa subsp. Pekinensis]	M4D0H5	108877.01	38.85	0.88	0.79	0.91
	Uncharacterized protein [Brassica rapa subsp. Pekinensis]	M4D4W9	82459.44	42.27	0.47	0.59	0.48
	Uncharacterized protein [Brassica rapa subsp. Pekinensis]	M4D4X7	78018.78	8.30	1.33	1.21	1.16
	Uncharacterized protein [Brassica rapa subsp. Pekinensis]	M4DBB4	110767.47	91.10	0.81	0.87	0.78
	Uncharacterized protein [Brassica rapa subsp. Pekinensis]	M4DBD7	29616.87	93.46	1.20	1.39	1.28
	Uncharacterized protein [Brassica rapa subsp. Pekinensis]	M4DDQ4	129791.12	34.63	0.72	0.74	0.72
	Uncharacterized protein [Brassica rapa subsp. Pekinensis]	M4E0A4	129321.59	32.49	0.95	0.95	0.90
	Uncharacterized protein [Brassica rapa subsp. Pekinensis]	M4E5W7	68438.35	11.21	0.91	0.99	0.82
	Uncharacterized protein [Brassica rapa subsp. Pekinensis]	M4EDI7	32342.03	22.66	0.65	0.70	0.80
	Uncharacterized protein [Brassica rapa subsp. Pekinensis]	M4EIH4	50512.53	64.32	0.80	0.64	0.71
	Uncharacterized protein [Brassica rapa subsp. Pekinensis]	M4EKI0	106324.79	8.72	1.51	1.56	1.47
	Uncharacterized protein [Brassica rapa subsp. Pekinensis]	M4ESF6	75437.18	11.21	0.81	0.72	0.79
	Uncharacterized protein [Brassica rapa subsp. Pekinensis]	M4F2D6	42073.85	33.26	0.66	0.73	0.66
	Uncharacterized protein [Brassica rapa subsp. Pekinensis]	M4F3A6	56199.87	68.49	0.86	0.90	0.75

	Uncharacterized protein [Brassica rapa subsp. Pekinensis]	M4F3X4	102850.55	39.13	0.94	0.90	0.87
	Uncharacterized protein [Brassica rapa subsp. Pekinensis]	M4F5J5	62445.64	52.21	1.39	1.56	1.32
	Uncharacterized protein [Capsella rubella]	R0FEY0	64619.50	76.91	0.62	0.56	0.57
	Uncharacterized protein [Capsella rubella]	R0GUT4	54032.71	8.51	0.97	0.82	0.88
	Uncharacterized protein [Capsella rubella]	R0GX19	31485.02	78.30	0.78	0.72	0.76
	Uncharacterized protein [Capsella rubella]	R0H4M9	27357.82	20.13	1.48	1.59	1.33
	Uncharacterized protein [Capsella rubella]	R0GZK0	59467.57	33.41	0.66	0.76	0.67
	Uncharacterized protein [Capsella rubella]	R0F2M3	42470.93	85.16	0.99	0.92	0.94
	Uncharacterized protein [Capsella rubella]	R0FA37	69271.96	61.51	0.89	0.87	0.86
	Uncharacterized protein [Capsella rubella]	R0IB62	97919.39	65.46	1.26	1.49	1.55
	Uncharacterized protein [Capsella rubella]	R0IM72	91118.38	88.51	0.71	0.70	0.75
	Uncharacterized protein [Capsella rubella]	R0G8M4	58641.25	99.91	1.01	1.00	1.01
	Uncharacterized protein [Capsella rubella]	R0HME7	98714.19	85.16	0.50	0.55	0.47
	Uncharacterized protein [Capsella rubella]	R0FTY4	35594.21	83.31	0.56	0.69	0.66
	Uncharacterized protein [Capsella rubella]	R0HUS3	129948.18	63.15	0.73	0.62	0.74
	Uncharacterized protein [Capsella rubella]	R0G1H0	50910.35	99.98	1.12	1.19	1.00
	Uncharacterized protein [Capsella rubella]	R0GAI4	45304.91	80.30	1.07	1.07	1.02
	Uncharacterized protein [Coccomyxa subellipsoidea]	I0YIK5	113766.18	88.85	0.90	0.80	0.90
	Uncharacterized protein [Coccomyxa subellipsoidea]	I0YUB5	147469.47	35.67	0.95	1.01	1.02
	Uncharacterized protein [Coccomyxa subellipsoidea]	I0YM79	32269.66	27.99	0.97	0.94	1.10
	Uncharacterized protein [Genlisea aurea]	S8CSX8	67901.45	61.86	1.28	1.22	1.28
	Uncharacterized protein [Genlisea aurea]	S8CFI1	50042.88	96.41	1.00	0.95	0.98

	Uncharacterized protein [Genlisea aurea]	S8DX56	51173.18	58.85	1.02	1.00	1.09
	Uncharacterized protein [Glycine max]	K7K4I0	189251.81	33.41	0.74	0.61	0.67
	Uncharacterized protein [Glycine max]	I1K7V7	64828.46	38.14	0.96	0.99	1.00
	Uncharacterized protein [Glycine max]	K7MZ42	190129.11	97.93	1.10	1.08	0.97
	Uncharacterized protein [Glycine max]	K7KY44	85231.28	97.34	0.71	0.59	0.69
	Uncharacterized protein [Glycine max]	K7KFV1	104961.24	78.72	1.13	0.95	0.94
	Uncharacterized protein [Glycine max]	I1LRQ4	119669.42	94.99	1.24	1.25	1.36
	Uncharacterized protein [Glycine max]	I1KC63	57158.04	94.11	1.00	1.06	1.01
	Uncharacterized protein [Glycine max]	I1M637	100139.43	68.06	0.56	0.58	0.50
	Uncharacterized protein [Glycine max]	I1MS26	128567.97	67.01	1.03	1.16	1.08
	Uncharacterized protein [Glycine max]	K7MRS0	18993.56	66.93	0.69	0.68	0.77
	Uncharacterized protein [Glycine max]	I1JB40	112946.77	44.62	1.39	1.46	1.30
	Uncharacterized protein [Glycine max]	K7M7V2	77781.00	44.49	1.25	1.07	1.35
	Uncharacterized protein [Glycine max]	K7L4M4	82097.97	28.16	0.87	0.77	0.95
	Uncharacterized protein [Glycine max]	K7MGD2	44425.23	18.65	1.11	1.04	1.10
	Uncharacterized protein [Glycine max]	K7LZ39	164634.89	14.42	0.88	0.97	0.99
	Uncharacterized protein [Glycine max]	I1JHQ5	66356.23	48.70	0.78	0.82	0.75
	Uncharacterized protein [Hordeum vulgare var. distichum]	M0X1P8	44030.95	97.52	1.20	1.21	1.19
	Uncharacterized protein [Hordeum vulgare var. distichum]	M0UFS8	51357.59	95.11	0.99	1.00	0.99
	Uncharacterized protein [Hordeum vulgare var. distichum]	M0WBQ5	22493.28	64.97	0.68	0.58	0.72
	Uncharacterized protein [Hordeum vulgare var. distichum]	M0VZX7	19406.41	49.26	1.26	1.16	1.24
	Uncharacterized protein [Hordeum vulgare var. distichum]	M0VW25	81331.53	46.13	1.21	1.32	1.33

	Uncharacterized protein [Hordeum vulgare var. distichum]	M0Z7U4	42646.69	38.71	0.76	0.77	0.78
	Uncharacterized protein [Hordeum vulgare var. distichum]	M0X066	187078.73	38.32	0.62	0.70	0.62
	Uncharacterized protein [Hordeum vulgare var. distichum]	M0W4P2	142798.63	26.99	0.80	0.76	0.78
	Uncharacterized protein [Hordeum vulgare var. distichum]	M0YXC0	86708.65	21.45	1.68	1.99	1.56
	Uncharacterized protein [Hordeum vulgare var. distichum]	M0X9D8	108596.80	16.37	0.72	0.80	0.73
	Uncharacterized protein [Hordeum vulgare var. distichum]	M0YZF1	11317.19	13.63	0.69	0.70	0.80
	Uncharacterized protein [Hordeum vulgare var. distichum]	M0Y799	34139.43	7.45	1.01	1.05	1.05
	Uncharacterized protein [Hordeum vulgare var. distichum]	M0YHV9	45088.80	2.42	0.77	0.79	0.80
	Uncharacterized protein [Lotus japonicus]	I3SL28	18177.38	9.56	1.10	1.27	1.08
	Uncharacterized protein [Musa acuminata subsp. Malaccensis]	M0TV14	90509.33	60.25	0.99	1.35	1.35
	Uncharacterized protein [Musa acuminata subsp. Malaccensis]	M0S5J0	23649.21	34.48	1.00	1.21	0.89
	Uncharacterized protein [Musa acuminata subsp. Malaccensis]	M0S873	113772.09	1.97	0.99	1.36	1.19
	Uncharacterized protein [Musa acuminata subsp. Malaccensis]	M0TC15	54788.30	95.60	0.81	0.89	0.97
	Uncharacterized protein [Musa acuminata subsp. Malaccensis]	M0SL68	192100.66	78.90	2.04	2.31	2.28
	Uncharacterized protein [Musa acuminata subsp. Malaccensis]	M0SIX6	21598.79	72.18	0.69	0.68	0.77
	Uncharacterized protein [Musa acuminata subsp. Malaccensis]	M0RXL8	6477.26	70.53	1.10	1.07	1.05
	Uncharacterized protein [Musa acuminata subsp. Malaccensis]	M0SQK6	62777.88	70.46	0.57	0.58	0.54
	Uncharacterized protein [Musa acuminata subsp. Malaccensis]	M0RQG3	25788.62	49.49	0.99	0.96	1.01
	Uncharacterized protein [Musa acuminata subsp. Malaccensis]	M0RXF0	35659.45	49.02	1.56	1.52	1.31
	Uncharacterized protein [Musa acuminata subsp. Malaccensis]	M0S6Z1	18735.74	42.54	0.66	0.51	0.69
	Uncharacterized protein [Musa acuminata subsp. Malaccensis]	M0RMG8	52211.68	32.95	0.77	0.71	0.86
	Uncharacterized protein [Musa acuminata subsp. Malaccensis]	M0TXW6	32263.01	26.31	1.13	1.18	1.28

	Uncharacterized protein [Musa acuminata subsp. Malaccensis]	M0U685	64439.47	25.80	3.49	4.69	4.60
	Uncharacterized protein [Musa acuminata subsp. Malaccensis]	M0RL26	51740.47	5.07	0.43	0.43	0.42
	Uncharacterized protein [Oryza brachyantha]	J3KWR5	29864.41	30.12	1.08	1.02	1.10
	Uncharacterized protein [Oryza brachyantha]	J3LKT0	49097.39	72.43	0.47	0.49	0.48
	Uncharacterized protein [Oryza brachyantha]	J3LQU1	119861.82	68.28	0.62	0.58	0.60
	Uncharacterized protein [Oryza brachyantha]	J3LUA9	89728.16	90.06	0.19	0.27	0.21
	Uncharacterized protein [Oryza brachyantha]	J3M3C8	39142.05	77.75	0.87	0.92	0.87
	Uncharacterized protein [Oryza brachyantha]	J3M7X2	44632.08	80.35	1.21	1.32	1.33
	Uncharacterized protein [Oryza brachyantha]	J3MAP3	137993.86	45.75	0.50	0.55	0.47
	Uncharacterized protein [Oryza brachyantha]	J3MD21	208562.36	69.77	1.22	1.19	1.09
	Uncharacterized protein [Oryza brachyantha]	J3MLN9	57984.46	99.61	1.90	2.07	2.02
	Uncharacterized protein [Oryza brachyantha]	J3MPL9	37609.97	68.42	0.64	0.56	0.59
	Uncharacterized protein [Oryza brachyantha]	J3MUE2	97795.07	57.50	0.90	0.91	1.21
	Uncharacterized protein [Oryza brachyantha]	J3N1D0	45954.49	30.12	0.77	0.69	0.67
	Uncharacterized protein [Oryza brachyantha]	J3NC64	160202.91	97.24	0.87	0.88	0.85
	Uncharacterized protein [Oryza glaberrima]	I1PV28	58724.23	88.56	1.01	0.92	0.98
	Uncharacterized protein [Oryza glaberrima]	I1Q2H2	59781.50	87.60	1.33	1.23	1.29
	Uncharacterized protein [Oryza glaberrima]	I1QBK2	34471.50	96.30	0.97	0.94	1.01
	Uncharacterized protein [Oryza glaberrima]	I1NUU3	45831.21	95.25	0.60	0.40	0.62
	Uncharacterized protein [Oryza glaberrima]	I1PU71	15573.34	91.62	0.61	0.60	0.69
	Uncharacterized protein [Oryza glaberrima]	I1PRX2	42175.50	85.13	0.80	1.24	1.10
	Uncharacterized protein [Oryza glaberrima]	I1P1X1	33957.83	84.78	1.01	1.01	0.89

	Uncharacterized protein [Oryza glaberrima]	I1NY08	30307.85	63.49	1.31	1.20	1.31
	Uncharacterized protein [Oryza glaberrima]	I1Q8R8	10900.75	55.19	0.87	0.78	0.91
	Uncharacterized protein [Oryza glaberrima]	I1Q8W1	21198.78	30.28	1.08	0.94	1.06
	Uncharacterized protein [Oryza glaberrima]	I1QU54	46070.21	23.37	0.83	0.77	0.77
	Uncharacterized protein [Oryza glaberrima]	I1PRT4	40009.63	21.95	0.41	0.45	0.43
	Uncharacterized protein [Oryza glaberrima]	I1Q6Q6	12218.42	19.39	0.76	0.66	0.75
	Uncharacterized protein [Oryza glaberrima]	I1Q4R0	137548.31	7.02	0.55	0.66	0.54
	Uncharacterized protein [Oryza glaberrima]	I1PSX8	47234.61	0.83	0.85	0.80	0.72
	Uncharacterized protein [Physcomitrella patens subsp. Patens]	A9S9S2	36031.50	90.93	0.00	0.00	0.00
	Uncharacterized protein [Populus trichocarpa]	B9GQX8	53216.87	50.07	2.57	2.50	3.05
	Uncharacterized protein [Populus trichocarpa]	B9GYL6	40049.94	31.23	1.03	0.85	1.05
	Uncharacterized protein [Populus trichocarpa]	B9H3L1	38573.60	21.41	0.96	0.98	1.10
	Uncharacterized protein [Populus trichocarpa]	B9H6X4	90016.43	98.53	0.50	0.52	0.52
	Uncharacterized protein [Populus trichocarpa]	B9HB68	91860.70	1.29	1.11	1.47	1.18
	Uncharacterized protein [Populus trichocarpa]	B9HLY8	76737.76	57.30	1.00	0.76	0.89
	Uncharacterized protein [Populus trichocarpa]	B9HLY9	162113.63	41.23	0.73	0.81	0.75
	Uncharacterized protein [Populus trichocarpa]	B9HYW2	17039.30	39.13	1.43	1.44	1.34
	Uncharacterized protein [Populus trichocarpa]	B9I2S7	13255.09	19.95	1.58	1.35	1.24
	Uncharacterized protein [Populus trichocarpa]	B9I329	41966.51	42.27	1.48	1.54	1.40
	Uncharacterized protein [Populus trichocarpa]	U5FV98	61467.16	86.44	0.57	0.48	0.56
	Uncharacterized protein [Populus trichocarpa]	B9IHK0	215791.69	15.41	0.91	0.99	0.82
	Uncharacterized protein [Prunus persica]	M5X6U0	247923.56	82.41	0.89	0.87	1.05

	Uncharacterized protein [Prunus persica]	M5VP10	152269.86	19.39	1.29	1.48	1.37
	Uncharacterized protein [Prunus persica]	M5WFP0	124778.46	66.78	0.87	0.77	0.97
	Uncharacterized protein [Prunus persica]	M5XA02	121750.41	94.62	1.65	1.92	1.60
	Uncharacterized protein [Prunus persica]	M5XAN4	93249.20	78.45	0.24	0.22	0.30
	Uncharacterized protein [Prunus persica]	M5WHY1	22341.38	68.64	1.67	1.78	1.56
	Uncharacterized protein [Prunus persica]	M5VMT0	18497.88	61.51	0.95	0.81	0.86
	Uncharacterized protein [Prunus persica]	M5X3C8	17463.31	39.83	1.08	0.84	1.11
	Uncharacterized protein [Prunus persica]	M5WPV4	114200.47	25.12	2.51	2.24	2.48
	Uncharacterized protein [Prunus persica]	M5WXT7	69253.01	11.86	0.63	0.69	0.64
	Uncharacterized protein [Prunus persica]	M5WKT6	51425.79	0.37	0.76	0.73	0.77
	Uncharacterized protein [Prunus persica]	M5W8H1	57151.54	13.83	0.96	0.89	0.94
	Uncharacterized protein [Prunus persica]	M5WQX5	60501.55	67.76	1.17	1.05	1.01
	Uncharacterized protein [Prunus persica]	M5XJY6	122572.63	49.02	1.03	0.94	1.04
	Uncharacterized protein [Setaria italica]	K3XE42	122919.26	25.46	0.55	0.47	0.54
	Uncharacterized protein [Setaria italica]	K3XEY6	83033.96	75.15	0.51	0.45	0.53
	Uncharacterized protein [Setaria italica]	K3XPP6	53864.65	57.40	1.44	1.49	1.38
	Uncharacterized protein [Setaria italica]	K3XRZ6	149966.06	30.92	1.24	1.53	1.59
	Uncharacterized protein [Setaria italica]	K3XTC8	65833.05	99.94	1.00	0.92	0.94
	Uncharacterized protein [Setaria italica]	K3XVX2	74834.95	56.11	0.70	0.74	0.73
	Uncharacterized protein [Setaria italica]	K3XW06	63989.59	93.74	0.73	0.70	0.71
	Uncharacterized protein [Setaria italica]	K3YAZ5	10888.10	41.87	0.93	1.12	0.93
	Uncharacterized protein [Setaria italica]	K3YN92	56027.94	15.59	1.22	1.19	1.09

	Uncharacterized protein [Setaria italica]	K3YPN8	117283.76	43.72	0.72	0.70	0.77
	Uncharacterized protein [Setaria italica]	K3YRS1	63452.62	75.94	1.18	1.31	1.30
	Uncharacterized protein [Setaria italica]	K3Z082	70255.46	19.02	1.15	1.13	1.11
	Uncharacterized protein [Setaria italica]	K3Z584	65376.14	92.78	0.75	0.84	0.76
	Uncharacterized protein [Setaria italica]	K3ZCQ3	21578.38	19.58	0.82	0.88	0.83
	Uncharacterized protein [Setaria italica]	K3ZD60	50952.17	53.29	0.96	1.05	0.91
	Uncharacterized protein [Setaria italica]	K3ZMH9	28977.38	78.10	0.75	0.91	0.75
	Uncharacterized protein [Setaria italica]	K3ZXP9	16786.74	47.47	0.75	0.59	0.67
	Uncharacterized protein [Setaria italica]	K4A219	92117.10	30.12	0.77	0.72	0.73
	Uncharacterized protein [Setaria italica]	K4A4R9	195224.44	26.99	1.37	1.26	1.42
	Uncharacterized protein [Setaria italica]	K4A6J3	87690.68	89.00	0.65	0.60	0.56
	Uncharacterized protein [Solanum lycopersicum]	K4B822	95301.97	74.10	0.52	0.72	0.65
	Uncharacterized protein [Solanum lycopersicum]	K4C0V5	13851.52	0.37	0.97	1.03	1.05
	Uncharacterized protein [Solanum lycopersicum]	K4C150	23041.80	64.57	0.80	0.82	0.79
	Uncharacterized protein [Solanum lycopersicum]	K4CQE0	69888.70	0.00	0.93	0.84	0.88
	Uncharacterized protein [Solanum lycopersicum]	K4CUA6	40111.44	99.95	1.48	1.57	1.53
	Uncharacterized protein [Solanum lycopersicum]	K4CUD2	28459.29	1.97	0.84	0.88	0.94
	Uncharacterized protein [Solanum lycopersicum]	K4CWY8	41302.64	78.40	0.62	0.60	0.53
	Uncharacterized protein [Solanum lycopersicum]	K4D904	12484.95	53.51	1.11	1.23	1.20
	Uncharacterized protein [Solanum lycopersicum]	K4D906	16852.16	52.86	0.67	0.64	0.66
	Uncharacterized protein [Solanum tuberosum]	M0ZN85	44992.63	94.69	0.59	0.71	0.60
	Uncharacterized protein [Solanum tuberosum]	M1ADP8	53819.19	18.08	0.95	0.95	0.90

	Uncharacterized protein [Solanum tuberosum]	M1ADX4	28558.04	37.43	0.89	0.78	0.86
	Uncharacterized protein [Solanum tuberosum]	M1AEK3	57872.68	88.10	1.91	1.89	1.75
	Uncharacterized protein [Solanum tuberosum]	M1AVL1	21091.50	0.37	0.82	0.80	0.79
	Uncharacterized protein [Solanum tuberosum]	M1B6I8	64228.31	90.91	0.00	0.00	0.00
	Uncharacterized protein [Solanum tuberosum]	M1BUJ1	59438.61	54.67	0.77	0.66	0.69
	Uncharacterized protein [Solanum tuberosum]	M1C6W8	80930.64	3.09	1.07	1.41	1.27
	Uncharacterized protein [Solanum tuberosum]	M1D1J4	11375.25	69.49	0.69	0.77	0.80
	Uncharacterized protein [Solanum tuberosum]	M1D6T0	11760.60	57.30	1.15	1.10	1.00
	Uncharacterized protein [Solanum tuberosum]	M1DA41	13622.29	76.21	0.97	0.96	0.94
	Uncharacterized protein [Solanum tuberosum]	M1DK09	45196.45	62.21	1.10	1.20	1.15
	Uncharacterized protein [Solanum tuberosum]	M1ANS2	34431.16	90.42	0.64	0.61	0.63
	Uncharacterized protein [Solanum tuberosum]	M1C829	31625.76	82.49	0.89	0.74	0.82
	Uncharacterized protein [Triticum urartu]	M8A8L3	45835.39	38.29	0.72	0.75	0.80
	Uncharacterized protein [Triticum urartu]	M7ZTN4	60245.25	83.50	0.98	1.01	1.10
	Uncharacterized protein [Triticum urartu]	M7YL19	7749.90	86.25	1.32	1.48	1.53
	Uncharacterized protein [Triticum urartu]	M7ZZH0	42097.23	11.21	0.89	0.96	0.92
	Uncharacterized protein [Triticum urartu]	M7Z8H0	15959.72	3.98	0.73	0.68	0.78
	Uncharacterized protein [Triticum urartu]	M7YJG5	32912.01	52.42	0.54	0.51	0.53
	Uncharacterized protein [Triticum urartu]	M8ABJ8	61337.57	97.10	1.34	1.24	1.25
	Uncharacterized protein [Triticum urartu]	T1M8B5	36465.57	68.42	1.16	1.21	1.08
	Uncharacterized protein [Triticum urartu]	T1LIZ1	140434.58	49.14	1.13	1.29	1.20
	Uncharacterized protein [Zea mays]	K7UXI7	32628.77	33.41	1.09	1.14	1.05

	Uncharacterized protein [Zea mays]	K7UYL7	18006.22	94.77	1.34	1.40	1.35
	Uncharacterized protein [Zea mays]	K7UBP3	11652.63	1.06	0.61	0.51	0.71
	Uncharacterized protein [Zea mays]	K7UF15	16796.69	80.30	1.21	1.06	1.18
	Uncharacterized protein [Zea mays]	C0PKL5	32097.86	96.21	0.71	0.66	0.78
	Uncharacterized protein [Zea mays]	C0P9I7	85309.82	66.47	0.95	1.04	1.04
	Uncharacterized protein [Zea mays]	C0PMM3	41221.54	61.77	1.10	1.06	1.00
	Uncharacterized protein [Zea mays]	C0P5K0	24068.01	39.97	0.68	0.51	0.73
	Uncharacterized protein [Zea mays]	C0PCW2	80024.12	25.97	0.81	0.85	0.88
	Uncharacterized protein [Zea mays]	B4FZR9	36654.90	2.42	1.58	1.88	1.79
	Unnamed protein product [Bathycoccus prasinos]	K8ERC9	68413.23	6.16	0.74	0.71	0.88
